# Relationship between *XPA*, *XPB/ERCC3*, *XPF/ERCC4*, and *XPG/ERCC5* Polymorphisms and the Susceptibility to Head and Neck Carcinoma: A Systematic Review, Meta-Analysis, and Trial Sequential Analysis

**DOI:** 10.3390/medicina60030478

**Published:** 2024-03-14

**Authors:** Mohammad Moslem Imani, Masoumeh Basamtabar, Sattar Akbari, Edris Sadeghi, Masoud Sadeghi

**Affiliations:** 1 Department of Orthodontics, School of Dentistry, Kermanshah University of Medical Sciences, Kermanshah 6713954658, Iran; mmoslem.imani@yahoo.com (M.M.I.);; 2 Medical Biology Research Centre, Kermanshah University of Medical Sciences, Kermanshah 6714415185, Iran; sadeghi_mkn@yahoo.com

**Keywords:** head and neck carcinoma, xeroderma pigmentosum, polymorphism, meta-analysis

## Abstract

*Background and Objectives*: Nucleotide Excision Repair (NER), the most extensively researched DNA repair mechanism, is responsible for repairing a variety of DNA damages, and Xeroderma Pigmentosum (XP) genes participate in NER. Herein, we aimed to update the previous results with a meta-analysis evaluating the association of XPA, XPB/ERCC3, XPF/ERCC4, and XPG/ERCC5 polymorphisms with the susceptibility to HNC. *Materials and Methods*: PubMed/Medline, Web of Science, Scopus, and Cochrane Library databases were searched without any restrictions until 18 November 2023 to find relevant studies. The Review Manager 5.3 (RevMan 5.3) software was utilized to compute the effect sizes, which were expressed as the odds ratio (OR) with a 95% confidence interval (CI). *Results*: Nineteen articles were involved in the systematic review and meta-analysis that included thirty-nine studies involving ten polymorphisms. The results reported that the CC genotype of *rs17655* polymorphism showed a significantly decreased risk of HNC in the recessive model (OR: 0.89; 95%CI: 0.81, 0.99; *p*-value is 0.03). In addition, the CT genotype (OR: 0.65; 95%CI: 0.48, 0.89; *p*-value is 0.008) of the *rs751402* polymorphism was associated with a decreased risk, and the T allele (OR: 1.28; 95%CI: 1.05, 1.57; *p*-value is 0.02), the TT (OR: 1.74; 95%CI: 1.10, 2.74; *p*-value is 0.02), and the TT + CT (OR: 2.22; 95%CI: 1.04, 4.74; *p*-value is 0.04) genotypes were associated with an increased risk of HNC. *Conclusions*: The analysis identified two polymorphisms, *rs17655* and *rs751402*, as being significantly associated with the risk of HNC. The study underscored the influence of various factors, such as the type of cancer, ethnicity, source of control, and sample size on these associations.

## 1. Introduction

According to the most recent GLOBOCAN data (2020), head and neck cancer (HNC) is the seventh most prevalent cancer worldwide, with approximately 890,000 new cases (about 4.5% of all global cancer diagnoses) and 450,000 deaths annually (about 4.6% of all global cancer deaths) [1], and both its incidence and mortality rates are increasing [2,3]. This concerning trend is largely due to avoidable risk factors such as tobacco and alcohol consumption, areca nut ingestion, and sexually transmitted HPV infections [1]. HNC originates from epithelial cells and typically develops in the oral cavity, pharynx, and larynx [4].

While environmental carcinogens and cancer-causing viruses are the primary causative factors, it is clear that genetic predisposition also plays a significant role in modulating the risk of HNC [5,6,7,8,9]. Nucleotide Excision Repair (NER), the most extensively researched DNA repair mechanism, is responsible for repairing a variety of DNA damage, including thymidine dimers, oxidative DNA damage, bulky DNA adducts, and cross-links [10]. NER is a flexible system that detects and repairs DNA damage induced by both internal and external factors, including therapeutic drugs [11]. Mutations in NER factors can impair cellular health and lead to human diseases, such as Xeroderma Pigmentosum (XP) [12].

Xeroderma Pigmentosum (XP) is a rare genetic disorder that is inherited in an autosomal recessive manner. It is characterized by a deficiency in the NER mechanism due to single-nucleotide mutations [13]. XP can be further classified into seven unique subgroups, referred to as complementation groups, ranging from XPA to XPG. These are typically seen as a less severe variant form of the disorder [14]. Each of these complementation groups signifies the existence of a causative mutation in one of the seven XP genes that participate in NER [15].

DNA repair mechanisms are crucial in safeguarding cells from DNA damage and preserving genomic integrity [16]. The NER pathway is the main method for eliminating bulky adducts from DNA, serving as a key component of the cellular defense against a wide range of structurally unrelated DNA lesions [17]. The NER pathway comprises several stages: The initial stage of the NER pathway involves the identification of damage by a protein complex, which includes XPC. The subsequent stage entails the unwinding of the DNA by a complex that includes XPD and the excision of the damaged single-stranded nucleotide fragment by molecules such as XPG, ERCC1, and XPF [18,19,20].

Three meta-analyses reported the association between XP polymorphisms and the risk of HNC risk with different results [21,22,23], as well as original studies [24,25,26,27]. Therefore, we aimed to design a meta-analysis evaluating the association of *XPA*, *XPB/ERCC3*, *XPF/ERCC4*, and *XPG/ERCC5* polymorphisms with the susceptibility to HNC.

## 2. Materials and Methods

### 2.1. Study Design

The meta-analysis was carried out following the guidelines of the Preferred Reporting Items for Systematic Reviews and Meta-Analyses (PRISMA) [28]. The research question, framed in the context of PECO (population, exposure, comparison, and outcome), was as follows: is there a correlation between *XPA*, *XPB*, *XPF*, and *XPG* polymorphisms and the susceptibility to HNC in case–control studies?

### 2.2. Identification of Articles

A researcher (M.S.) conducted a thorough literature review using various databases, including PubMed/Medline, Web of Science, Scopus, and Cochrane Library, without any restrictions until 18 November 2023, to find relevant studies. Another researcher (E.S.) reviewed the titles and abstracts of the retrieved articles and obtained the full texts of those that met the inclusion criteria. The search strategy included (“xerodermapigmentosum” or “xeroderma pigmentosum” or “xeroderma pigmentosa” or “ERCC*” or “excision repair cross-complementing”) and (“oral” or “OSCC” or “oral squamous cell” or “head and neck” or “HNSCC“ or “salivary gland” or “nasopharyngeal” or “nasopharynx” or “tongue” or “nasopharyngeal” or “oropharyngeal” or “oropharynx” or “laryngeal” or “hypopharyngeal” or “oropharyngolaryngeal” or “hypopharynx” or “oral cavity”) and (“cancer*” or “tumor*” or “carcinoma*” or “neoplasm*”) and (“gene*” or “polymorphism*” or “variant*” or “genotype*” or “allele*”). To ensure no significant study was missed, the reference lists of the articles were also examined. The search and selection procedures were further confirmed by another author (M.M.I.).

### 2.3. Selection Criteria

The exclusion criteria include review articles, meta-analyses, and systematic reviews. Articles with incomplete data or those that did not include a control group are also excluded. Studies conducted on animals, conference papers, and comment papers are not considered. Duplicate studies and book chapters are also excluded. Studies where the control group included individuals with any systemic disease or where the cases were under treatment are not considered.

### 2.4. Data Summary

Two authors (E.S. and M.S.) collected the data from the included studies in the meta-analysis independently. Any differences that arose were settled through joint discussion.

### 2.5. Quality Evaluation

The quality of the studies was evaluated by one author (M.S.) using the Newcastle–Ottawa Scale (NOS) tool, a well-established method for assessing the quality of non-randomized studies in meta-analyses [29]. The NOS tool assigns a maximum score of 9 for case–control studies, with a score of 7 or above indicating high quality. The scores were then reviewed for accuracy by another author (E.S.). Any discrepancies between the authors were resolved through discussion, ensuring a thorough and consensus-based evaluation process.

### 2.6. Statistical Analyses

The Review Manager 5.3 (RevMan 5.3) software was utilized to compute the effect sizes, which were expressed as the odds ratio (OR) with a 95% confidence interval (CI). This reflected the prevalence of *XPA*, *XPB*, *XPF*, and *XPG* polymorphisms in patients with HNC and control subjects. The pooled OR’s significance was ascertained using the Z-test, with a two-sided p-value of less than 0.10 indicating significance. A random-effects model [30] was employed if *P*_heterogeneity_ was <0.10 (I^2^ > 50%), signifying significant heterogeneity. If the heterogeneity was not significant, a fixed-effect model [31] was used.

A subgroup analysis was conducted to pinpoint any significant differences in the pooled ORs within these groups. Additionally, a meta-regression analysis was performed using a random-effects model to depict a linear relationship between the auxiliary variables in the study and the effect size. The presence of publication bias was evaluated using a Begg’s funnel plot and Egger’s regression test. The *p*-values from both Egger’s and Begg’s tests were computed, with a two-sided *p*-value less than 0.10, indicating the presence of publication bias.

In terms of sensitivity analysis, two methods were employed to assess the stability and consistency of the pooled ORs: The “one-study-removed” analysis was conducted to ascertain if any single study had a disproportionate impact on the overall estimate. The “cumulative” analysis was conducted to evaluate the impact of each additional study on the overall estimate. All these analyses related to publication bias and sensitivity analyses were performed using the Comprehensive Meta-Analysis version 3.0 (CMA 3.0) software.

The STRING database version 12.0 (https://string-db.org/), used for protein–protein interaction (PPI) network analysis, was utilized to investigate the functional interactions among the genes under studyThe sources of interaction and species were restricted to “Homo sapiens”, and an interation score exceeding 0.900 was used to construct the PPI networks. In these networks, the nodes represent proteins, and the edges represent the interactions between them. This method was employed to explore potential interactions among differentially expressed genes (DEGs) associated with various tissues.

To mitigate the risk of drawing false-positive or negative conclusions from meta-analyses [32], a TSA was performed. This analysis was conducted using the TSA software (version 0.9.5.10 beta) [33]. The TSA allows for the setting of a futility threshold, which can identify a non-effect outcome before the necessary information size is reached. The required information size (RIS) was determined with an alpha risk of 5%, a beta risk of 20%, and a two-sided boundary type. The heterogeneity (D^2^) was evaluated for the prevalence of the *XPA* and *XPG* polymorphisms in HNC patients and controls. If the Z-curve intersected the RIS line, it suggested that the studies included an adequate number of cases and that the conclusions were trustworthy. If not, it indicated that the available information was inadequate and more data were needed.

## 3. Results

### 3.1. Study Selection

**Figure 1:**
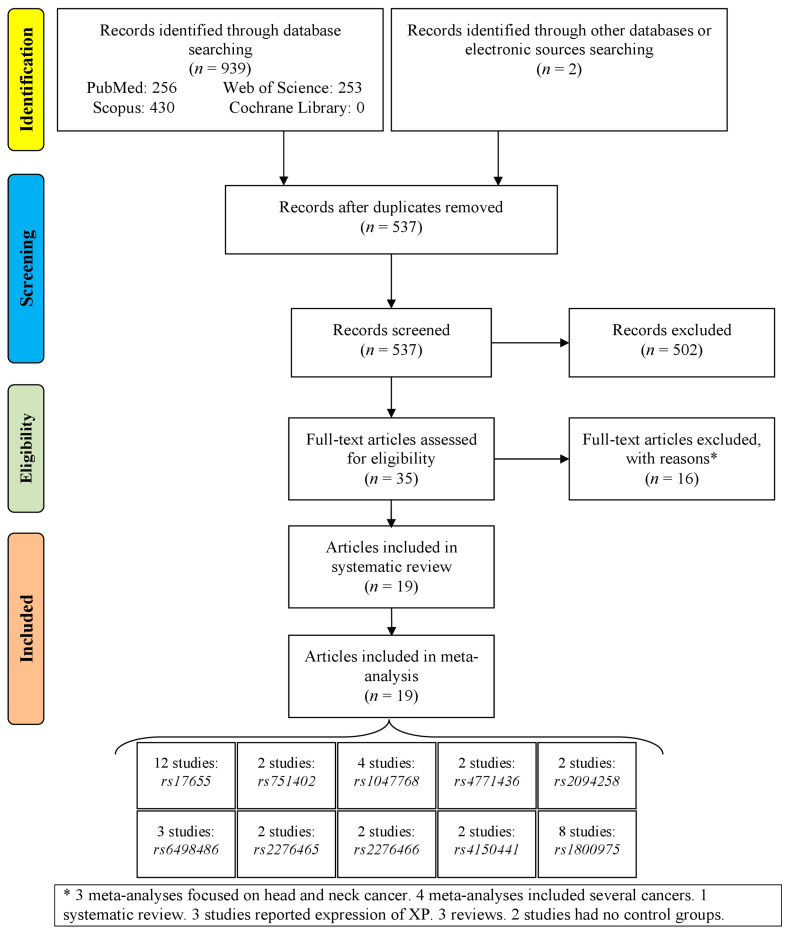
Flowchart of study selection.

A total of 941 records were identified among the databases and the electronic sources (Figure 1). After removing irrelevant records, reading the titles/abstracts, and then excluding full-text articles with reasons, 19 articles were involved in the systematic review and meta-analysis. These articles included 39 studies involving 10 polymorphisms.

### 3.2. Characteristics of the Articles

Nineteen articles [24,25,26,27,34,35,36,37,38,39,40,41,42,43,44,45,46,47,48] were entered into the meta-analysis (Table 1). The studies were published from 2006 to 2019. Twelve studies [24,25,27,34,35,37,39,40,41,43,45,47] reported the *rs17655* polymorphism, two [44,46] reported the *rs751402* polymorphism, four [24,34,39,47] reported the *rs1047768* polymorphism, two [39,47] reported the *rs4771436* polymorphism, two [39,42] reported the *rs2094258* polymorphism, three [24,27,44] reported the *rs6498486* polymorphism, two [24,27] reported the *rs2276465* and *rs4150441* polymorphisms, two [24,48] reported the *rs2276466* polymorphism, and eight [24,26,27,34,35,36,38,41] reported the *rs1800975* polymorphism.

### 3.3. Pooled Analysis

A summary of forest plot analyses is reported for the association of each polymorphism in five genetic models with the risk of HNC (Table 2). The forest plots are included in Supplementary File S1. The results reported that the CC genotype of *rs17655* polymorphism showed a significantly decreased risk of HNC in the recessive model (OR: 0.89; 95%CI: 0.81, 0.99; *p*-value is 0.03). In addition, the CT genotype (OR: 0.65; 95%CI: 0.48, 0.89; *p*-value is 0.008) of the *rs751402* polymorphism was associated with a decreased risk, and the T allele (OR: 1.28; 95%CI: 1.05, 1.57; *p*-value is 0.02), the TT (OR: 1.74; 95%CI: 1.10, 2.74; *p*-value is 0.02), and the TT + CT (OR: 2.22; 95%CI: 1.04, 4.74; *p*-value is 0.04) genotypes were associated with an increased risk of HNC. Therefore, among ten polymorphisms, just the *rs17655* and *rs751402* polymorphisms were associated with the HNC risk.

### 3.4. Subgroup Analysis

A subgroup analysis was performed on the pooled analyses of two polymorphisms (*rs17655* and *rs1800975*) with sufficient studies (Table 3). With regards to the *rs17655* polymorphism, the results suggested that in the Asian population, individuals with the C allele or the CC genotype have a decreased risk of HNC. In larger studies (sample size ≥ 400), the CC + CT genotype had an increased risk of HNC. In laryngeal cancer cases, the CC genotype had a decreased risk of HNC. In hospital-based controls, the CC genotype and, in population-based controls, the CC + CT genotype had a decreased risk of HNC. Therefore, the findings suggested that the cancer subtype and the characteristics of the study population (such as ethnicity, control source, and sample size) can influence the association of the *rs17655* polymorphism with the risk of HNC.

With regards to *rs1800975* polymorphism, the results suggested that in the Caucasian population, individuals with the A allele and the A allele and AA and AA + GA genotypes have a decreased risk of HNC. In hospital-based controls and individuals with oral cancer, the AA genotype had a decreased risk of HNC. Therefore, the findings suggested that the cancer subtype and the characteristics of the study population (such as ethnicity and control source) can affect the association of *rs1800975* polymorphism with the risk of HNC.

### 3.5. Meta-Regression Analysis

Table 4 reports a random-effect meta-regression analysis for two polymorphisms with sufficient studies (*rs17655* and *rs1800975*). The results showed that the publication year, sample size, and quality score were not confounding factors for these polymorphisms.

### 3.6. Sensitivity Analysis

Both “cumulative” and “one-study-removed” analyses showed the stability of the pooled results for the *rs17655*, *rs1047768*, *rs6498486*, and *rs1800975* polymorphisms. To remove the studies with a deviation of HWE in controls showed that in contrast to the initial pooled analysis, the CC genotype of the *rs17655* polymorphism did not associate with the risk of HNC, but this removal did not change the initial pooled analysis of the *rs1800975* polymorphism (Table 5). Therefore, a deviation of HWE in controls can impact the association of *rs17655* polymorphism with the risk of HNC but not for the *rs1800975* polymorphism.

### 3.7. TSA

In other cases, the Z-curve not crossing the RIS line in the TSA for the *rs17655* and *rs1800975* polymorphisms associated with HNC risk in five genetic models suggests that the current evidence is not sufficient to conclusively determine the association. More trials may be needed to reach a definitive conclusion. Supplementary File S2 shows the TSA plots.

### 3.8. Publication Bias

Supplementary File S3 shows the funnel plots for the *rs17655* and *rs1800975* polymorphisms in five genetic models. Begg’s test showed publication bias for the *rs1800975* polymorphism in the allelic (*p* = 0.083) and the recessive (*p* = 0.083) models.

### 3.9. STRING Results

**Figure 2:**
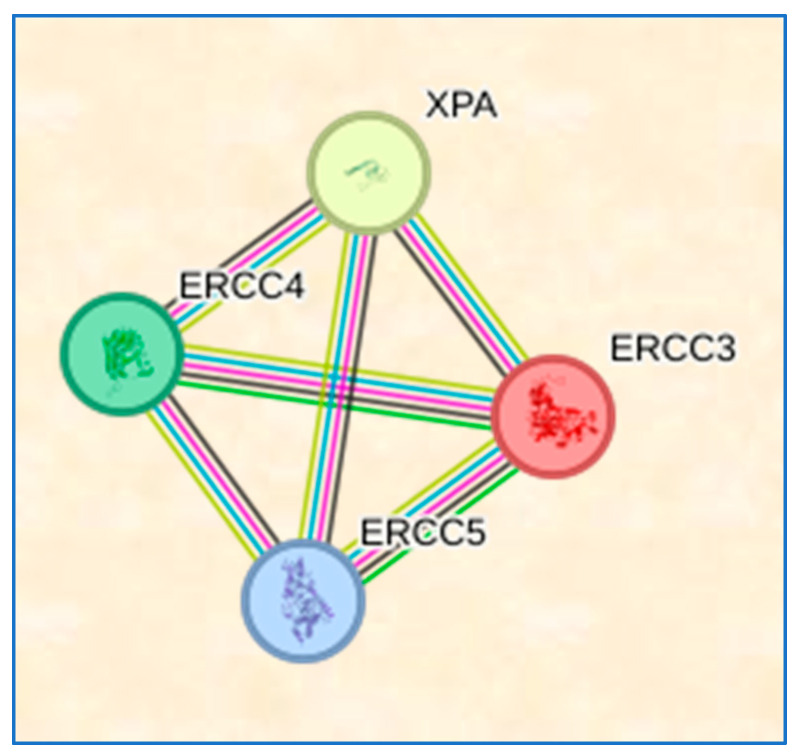
Protein–protein interaction (PPI) network of the examined genes. The nodes indicate proteins, and the edges indicate the number of interactions. Different colors represent different levels of evidence of a connection between proteins. This analysis had an average confidence score of more than 0.900. The PPI enrichment had a *p*-value less than 5.49 × 10^−11^, a total of 4 nodes and 6 edges, and an average node degree of 3.

Figure 2 shows the PPI network graph and heatmap for the XPA, XPB/ERCC3, XPF/ERCC4, and XPG/ERCC5 from the STRING database. Among the interactions, there are curated and experimental interactions between all XPs together.

## 4. Discussion

The analysis revealed that only two polymorphisms, *rs17655* and *rs751402*, were associated with HNC risk. The influence of factors such as the type of cancer, ethnicity, source of control, and sample size on these associations was highlighted in the subgroup analysis. The association of *rs17655* with HNC risk was affected by deviations from HWE in controls. Further studies may be required for a conclusive result. There was a moderate publication bias for *rs1800975* polymorphism. Interactions between all XPs were demonstrated in the PPI network.

NER is a well-researched pathway in the human body that repairs various forms of damage to double-helix DNA. This process involves four steps: identifying the lesion, marking and unwinding the damaged DNA segment, excising the oligonucleotide, and ligating new strands [42,49,50,51]. Variations in the core NER genes can alter the NER capability by affecting the expression and functionality of key proteins [42,52,53]. Several factors, including post-translational modifications and interactions with other proteins, regulate the proteins’ ability to engage in the NER pathway [54]. In our meta-analysis, the PPI demonstrated that XPA, XPB, XPF, and XPG have robust interactions.

Certain XP proteins are involved in pathways that include repairing oxidative damage, removing DNA cross-links, and transcription [55,56]. The study of XP genetics primarily centered on changes in expression levels and polymorphisms as they pertain to a range of physiological responses. These include susceptibility to specific types of cancer, reactions to DNA-damaging chemotherapy drugs, and aging [54]. The binding of XPA to damaged DNA is significantly enhanced by its interaction with other components of the NER [57,58,59]. DNA damage and repair can influence several cellular processes, such as replication and transcription, mutagenesis, and apoptosis. Therefore, they may play crucial roles in an organism’s development and pathology, including cancer [60].

A meta-analysis [61] indicated that XPA is a minor risk factor for the development of cancer. Another meta-analysis [62] evaluated XPG polymorphisms and found that *rs1047768* polymorphism was linked to an increased risk of lung cancer, *rs2227869* polymorphism was associated with a decreased risk of cancer in population-based studies, and *rs751402* and *rs873601* polymorphisms were connected to the risk of gastric cancer. A meta-analysis by Jiang et al. [21] suggested that the *rs17655* polymorphism was a risk factor for HNC susceptibility, particularly in laryngeal cancer and in the Asian population. However, another meta-analysis [22] suggested that the *rs17655* polymorphism may not be associated with the genetic susceptibility of HNC overall but might contribute to HNC susceptibility in the European population. The meta-analysis by Wu et al. [23] indicated that the XPA *rs1800975* polymorphism may not be associated with overall HNC susceptibility but with oral carcinoma susceptibility. The differences between results for subtypes of HNC can show that cancers originating from different sites in the HNC may have different tumor biology [63].

Our meta-analysis revealed that some XP polymorphisms are associated with HNC risk, and a subgroup analysis reported that factors such as the type of cancer, ethnicity, source of control, and sample size have an impact on this association.

This meta-analysis had many limitations: (1) The limited number of published studies on eight polymorphisms prevented us from conducting subgroup analysis or meta-regression analysis. (2) High heterogeneity was observed among several analyses, possibly due to the small number of studies. (3) There was a lack of adequate participants in the analyses based on TSA. (4) Many studies deviated from HWE in controls. On the other hand, the study had three strengths: (1) Most of the studies scored high in terms of quality. (2) Most analyses showed no publication bias. (3) The results were stable.

## 5. Conclusions

The analysis identified two polymorphisms, *rs17655* and *rs751402*, as being significantly associated with the risk of HNC. Specifically, the CC genotype of *rs17655* and the CT genotype of *rs751402* were linked to a decreased risk of HNC, while the T allele and TT and TT + CT genotypes of *rs751402* were connected to an increased risk. The study underscored the influence of various factors, such as the type of cancer, ethnicity, source of control, and sample size on these associations. However, the association of *rs17655* with HNC risk was found to be influenced by deviations from HWE in controls.

The findings could potentially guide the development of personalized treatment strategies for HNC based on a patient’s genetic profile. However, due to the moderate publication bias for the *rs1800975* polymorphism and the need for further validation of the results, additional studies are necessary for a more definitive conclusion. The observed interactions between all XPs in the PPI network also suggest a complex interplay of genetic factors in HNC, highlighting the need for a comprehensive understanding of these interactions in future research. This could pave the way for more effective prevention, early detection, and treatment strategies for HNC.

## Figures and Tables

**Table 1:** Characteristics of the articles.

The Study, Publication Year	Country	Ethnicity	Number of Cases/Controls	Control Source	Polymorphism: HWE *p*-Value in Controls	Genotyping Method	Tumor Site	Quality Score
Abbasi, 2009 [34]	Germany	Caucasian	248/647	PB	*rs17655*: <0.0001 *rs1047768*: 0.7616 *rs1800975*: 0.7373	PCR-RFLP	LC	8
An, 2007 [35]	USA	Caucasian	829/854	HB	*rs1800975*: 0.0010 *rs17655*: 0.4245	PCR	HNC	8
Avci, 2018 [25]	Turkey	Caucasian	111/148	PB	*rs17655*: 0.1716	PCR	OC	9
Bau, 2007 [36]	Taiwan	Asian	154/105	PB	*rs1800975*: 0.8954	PCR	OC	8
Cui, 2006 [37]	USA	Mixed	443/911	PB	*rs17655*: <0.0001	PCR	NPC	9
Hall, 2007 [26]	France	Caucasian	597/770	HB	*rs1800975*: 0.2248	PCR	HNC	8
Jelonek, 2010 [38]	Poland	Caucasians	66/113	PB	*rs1800975*: 0.0551	PCR	HNC	6
Li, 2014 [24]	China	Asian	211/210	HB	*rs17655*: 0.0003 *rs1047768*: 0.3326 *rs2276465*: 0.0023 *rs2276466*: 0.0970 *rs6498486*: 0.1517 *rs4150441*: 0.0034 *rs1800975*: <0.0001	PCR	LC	7
Lu, 2014 [27]	China	Asian	176/176	HB	*rs17655*: 0.0007 *rs2276465*: 0.0071 *rs6498486*: 0.1479 *rs4150441*: 0.0127 *rs1800975*: <0.0001	PCR	LC	7
Ma, 2012 [39]	USA	Mixed	1059/1059	PB	*rs17655*: 0.1749 *rs1047768*: 0.6694 *rs2094258*: 0.0920 *rs4771436*: 0.9424	PCR-RFLP	HNC	9
Nigam, 2019 [40]	India	Asian	67/288	PB	*rs17655*: 0.7511	PCR-RFLP	OC	8
Sugimura, 2006 [41]	Japan	Asian	122/241	HB	*rs17655*: <0.0001 *rs1800975*: 0.0496	PCR	OC	7
Sun, 2015 [42]	China	Asian	271/271	HB	*rs2094258*: 0.8255	PCR-RFLP	LC	8
Wen, 2006 [43]	China	Asian	175/525	HB	*rs17655*: 0.0026	PCR-RFLP	NPC	9
Xue, 2013 [44]	China	Asian	142/275	HB	*rs751402*: 0.3033 *rs6498486*: 0.4273	PCR-RFLP	OC	8
Yu, 2012 [48]	USA	Mixed	1040/1046	HB	*rs2276466*: 0.0636	PCR-RFLP	HNC	8
Yuan, 2012 [45]	China	Asian	394/884	HB	*rs17655*: <0.0001	PCR	HNC	8
Zavras, 2012 [46]	Taiwan	Asian	239/336	HB	*rs751402*: 0.3984	TaqMan and PCR	OC	6
Zhu, 2018 [47]	China	Asian	199/190	HB	*rs17655*: 0.6655 *rs1047768*: 0.3839 *rs4771436*: 0.5694	PCR	LC	8

HNC: head and neck cancer. OC: oral cancer. LC: laryngeal cancer. NPC: Nasopharyngeal cancer. HB: hospital-based. PB: population-based. HWE: Hardy–Weinberg equilibrium. PCR: Polymerase chain reaction. RFLP: Restriction fragment length polymorphism.

**Table 2:** Summary of forest plot analyses.

Polymorphism (N)	Genetic Model	OR	95%CI		Z-Value	*p*-Value	I^2^	*P*_heterogeneity_
			Min.	Max.				
*rs17655* (12)	C vs. G	0.95	0.86	1.05	1.03	0.30	56%	0.009
	CC vs. GG	0.86	0.75	1.00	1.98	0.05	34%	0.12
	GC vs. GG	1.26	0.94	1.71	1.53	0.13	85%	<0.00001
	CC + GC vs. GG	1.47	0.98	2.19	1.88	0.06	93%	<0.00001
	CC vs. GG + GC	0.89	0.81	0.99	2.14	**0.03**	31%	0.14
*rs751402* (2)	T vs. C	1.28	1.05	1.57	2.38	**0.02**	0%	0.55
	TT vs. CC	1.74	1.10	2.74	2.39	**0.02**	0%	0.75
	CT vs. CC	0.65	0.48	0.89	2.65	**0.008**	5%	0.31
	TT + CT vs. CC	2.22	1.04	4.74	2.07	**0.04**	87%	0.005
	TT vs. CC + CT	2.48	0.78	7.93	1.53	0.12	85%	0.01
*rs1047768* (4)	T vs. C	0.92	0.74	1.13	0.81	0.42	72%	0.01
	TT vs. CC	0.91	0.64	1.31	0.50	0.62	60%	0.06
	CT vs. CC	1.05	0.90	1.22	0.66	0.51	0%	0.96
	TT + CT vs. CC	1.06	0.92	1.22	0.74	0.46	0%	0.96
	TT vs. CC + CT	1.03	0.88	1.21	0.37	0.71	0%	0.99
*rs4771436* (2)	G vs. T	1.02	0.89	1.16	0.25	0.81	0%	0.68
	GG vs. TT	1.03	0.73	1.44	0.15	0.88	0%	0.61
	TG vs. TT	1.02	0.86	1.20	0.23	0.81	0%	0.92
	GG + TG vs. TT	3.08	0.33	28.49	0.99	0.32	98%	<0.00001
	GG vs. TT + TG	1.02	0.73	1.42	0.11	0.91	0%	0.62
*rs2094258* (2)	A vs. G	1.05	0.92	1.20	0.71	0.48	16%	0.28
	AA vs. GG	1.09	0.75	1.57	0.44	0.66	33%	0.22
	GA vs. GG	1.06	0.89	1.25	0.65	0.51	0%	0.64
	AA + GA vs. GG	1.06	0.9	1.24	0.69	0.49	0%	0.42
	AA vs. GG + GA	1.06	0.74	1.53	0.33	0.74	22%	0.26
*rs6498486* (3)	C vs. A	1.16	0.96	1.41	1.56	0.12	0%	0.93
	CC vs. AA	1.36	0.89	2.09	1.41	0.16	0%	0.96
	AC vs. AA	1.13	0.87	1.46	0.92	0.36	0%	0.99
	CC + AC vs. AA	1.17	0.92	1.50	1.28	0.20	0%	0.97
	CC vs. AA + AC	1.29	0.86	1.95	1.22	0.22	0%	0.96
*rs2276465* (2)	C vs. A	1.00	0.67	1.47	0.02	0.98	71%	0.07
	CC vs. AA	1.39	0.94	2.06	1.64	0.10	0%	0.97
	AC vs. AA	1.16	0.85	1.59	0.93	0.35	0%	0.96
	CC vs. AC + AA	0.74	0.28	1.96	0.60	0.55	92%	0.0003
	CC vs. AA + AC	0.93	0.48	1.80	0.22	0.83	69%	0.07
*rs2276466* (2)	G vs. C	0.96	0.85	1.09	0.58	0.56	0%	0.39
	GG vs. CC	0.30	0.02	4.54	0.87	0.39	98%	<0.00001
	CG vs. CC	1.08	0.92	1.28	0.94	0.35	0%	0.87
	GG + CG vs. CC	1.03	0.88	1.20	0.32	0.75	0%	0.77
	GG vs. CC + CG	0.84	0.50	1.39	0.69	0.49	50%	0.16
*rs4150441* (2)	G vs. A	1.09	0.88	1.34	0.80	0.43	0%	0.85
	GG vs. AA	1.20	0.80	1.80	0.90	0.37	0%	0.87
	AG vs. AA	1.12	0.82	1.53	0.70	0.48	0%	0.93
	GG + AG vs. AA	1.26	0.95	1.67	1.59	0.11	0%	0.47
	GG vs. AA + AG	1.08	0.74	1.57	0.38	0.70	0%	0.88
*rs1800975* (8)	A vs. G	0.78	0.49	1.23	1.07	0.28	96%	<0.00001
	AA vs. GG	0.91	0.77	1.07	1.11	0.27	17%	0.29
	GA vs. GG	1.00	0.82	1.23	0.02	0.99	55%	0.03
	AA + GA vs. GG	0.94	0.85	1.06	1.01	0.31	43%	0.09
	AA vs. GG + GA	0.66	0.35	1.26	1.26	0.21	94%	<0.00001

Bold data mean statistically significant (*p* < 0.05). OR: odds ratio. CI: confidence interval. N: Number of Studies.

**Table 3:** Subgroup analysis of two polymorphisms with sufficient studies.

Polymorphism (N)	Subgroup (N)	Variables	Allelic	Homozygous	Heterozygous	Dominant	Recessive
*rs17655* (12)	Ethnicity						
	Asian (7)	OR (95%CI)	0.87 (0.79, 0.97)	0.81 (0.67, 0.97)	1.05 (0.67, 1.65)	1.22 (0.66, 2.66)	0.86 (0.68, 1.09)
		*p*-value	**0.009**	**0.03**	0.83	0.52	0.22
		I^2^	67%	50%	85%	93%	52%
	Caucasian (2)	OR (95%CI)	1.01 (0.82, 1.24)	0.68 (0.37, 1.26)	1.98 (0.97, 4.06)	1.47 (0.98, 2.19)	0.64 (0.35, 1.17)
		*p*-value	0.23	0.22	0.06	0.06	0.15
		I^2^	0%	0%	81%	93%	0%
	Mixed (3)	OR (95%CI)	1.05 (0.96, 1.15)	1.01 (0.79, 1.28)	1.39 (0.85, 2.28)	1.76 (0.70, 4.42)	0.93 (0.81, 1.07)
		*p*-value	0.30	0.95	0.19	0.23	0.31
		I^2^	19%	0%	82%	96%	0%
	Sample size						
	≥400 (6)	OR (95%CI)	0.99 (0.92, 1.06)	0.89 (0.75, 1.05)	1.36 (0.90, 2.60)	1.74 (1.03, 2.93)	0.91 (0.82, 1.02)
		*p*-value	0.82	0.15	0.15	**0.04**	0.11
		I^2^	53%	28%	90%	95%	36%
	<400 (6)	OR (95%CI)	0.89 (0.77, 1.03)	0.80 (0.60, 1.07)	1.12 (0.74, 1.69)	1.14 (0.60, 2.18)	0.82 (0.64, 1.04)
		*p*-value	0.11	0.13	0.60	0.69	0.10
		I^2^	61%	50%	65%	88%	32%
	Control source						
	HB (7)	OR (95%CI)	0.90 (0.77, 1.05)	0.86 (0.64, 1.15)	1.13 (0.73, 1.73)	0.32 (0.74, 2.36)	0.90 (0.73, 1.10)
		*p*-value	0.18	0.31	0.59	**<0.0001**	0.29
		I^2^	67%	60%	84%	93%	56%
	PB (5)	OR (95%CI)	1.03 (0.93, 1.13)	0.86 (0.67, 1.10)	1.47 (0.89, 2.43)	1.69 (0.87, 3.28)	0.83 (0.69, 0.99)
		*p*-value	0.56	0.22	0.13	0.12	**0.04**
		I^2^	1%	0%	89%	95%	0%
	Cancer subtype						
	OC (3)	OR (95%CI)	1.01 (0.82, 1.25)	0.82 (0.59, 1.15)	1.37 (0.71, 2.66)	1.62 (0.65, 4.07)	1.02 (0.71, 1.48)
		*p*-value	0.91	0.25	0.35	0.30	0.90
		I^2^	10%	0%	75%	89%	0%
	LC (4)	OR (95%CI)	0.80 (0.62, 1.04)	0.60 (0.45, 0.76)	1.11 (0.54, 2.29)	0.90 (0.43, 1.89)	0.64 (0.51, 0.82)
		*p*-value	0.10	**0.0004**	0.77	0.78	**0.0003**
		I^2^	73%	20%	90%	93%	0%
	NPC (2)	OR (95%CI)	0.99 (0.94, 1.04)	0.98 (0.71, 1.35)	1.21 (0.35, 4.19)	2.67 (1.08, 6.63)	0.93 (0.77, 1.12)
		*p*-value	0.68	0.90	0.77	**0.03**	0.43
		I^2^	84%	0%	94%	92%	3%
*rs1800975* (8)	Ethnicity						
	Asian (4)	OR (95%CI)	0.99 (0.85, 1.15)	0.97 (0.73, 1.29)	1.22 (0.94, 2.95)	1.08 (0.86, 1.35)	0.89 (0.69, 1.14)
		*p*-value	0.88	0.86	0.13	0.53	0.35
		I^2^	33%	24%	0%	0%	48%
	Caucasian (3)	OR (95%CI)	0.53 (0.18, 1.59)	0.83 (0.52, 1.32)	0.80 (0.58, 1.10)	0.81 (0.69, 0.96)	0.26 (0.21, 0.33)
		*p*-value	**<0.0001**	0.43	0.17	**0.02**	**<0.0001**
		I^2^	98%	52%	60%	66%	96%
	Mixed (1)	OR (95%CI)	0.99 (0.86, 1.14)	0.91 (0.68, 1.22)	1.10 (0.90, 1.35)	1.05 (0.87, 1.27)	0.87 (0.66, 1.14)
		*p*-value	0.87	0.53	0.36	0.62	0.31
		I^2^	-	-	-	-	-
	Sample size						
	≥400 (4)	OR (95%CI)	0.72 (0.33, 1.57)	0.95 (0.79, 1.15)	0.97 (0.76, 1.24)	0.97 (0.78, 1.21)	0.63 (0.22, 1.82)
		*p*-value	0.41	0.62	0.81	0.81	0.39
		I^2^	98%	0%	98%	64%	97%
	<400 (4)	OR (95%CI)	0.87 (0.74, 1.03)	0.79 (0.57, 1.10)	1.08 (0.81, 1.44)	0.90 (0.67, 1.22)	0.76 (0.57, 1.01)
		*p*-value	0.12	0.17	0.59	0.50	0.06
		I^2^	44%	45%	48%	23%	43%
	Control source						
	HB (5)	OR (95%CI)	0.73 (0.38, 1.43)	0.91 (0.76, 1.09)	1.04 (0.79, 1.38)	0.96 (0.79, 1.17)	0.44 (0.37, 0.52)
		*p*-value	0.36	0.30	0.75	0.67	**<0.0001**
		I^2^	98%	11%	69%	51%	95%
	PB (3)	OR (95%CI)	0.91 (0.68, 1.20)	0.92 (0.63, 1.35)	0.97 (0.75, 1.26)	0.89 (0.59, 1.35)	0.83 (0.64, 1.08)
		*p*-value	0.50	0.68	0.83	0.59	0.17
		I^2^	54%	49%	32%	51%	31%
	Cancer subtype						
	OC (2)	OR (95%CI)	084 (0.67, 1.06)	0.70 (0.44, 1.13)	1.41 (0.93, 2.15)	0.96(0.66, 1.41)	0.66 (0.45, 0.96)
		*p*-value	0.14	0.15	0.11	0.84	**0.03**
		I^2^	4%	8%	0%	0%	36%
	LC (3)	OR (95%CI)	1.09 (0.94, 1.27)	1.16 (0.87, 1.55)	1.09 (0.87, 1.36)	1.11 (0.90, 1.36)	1.12 (0.85, 1.46)
		*p*-value	0.24	0.31	0.47	0.32	0.42
		I^2^	0%	0%	0%	0%	0%

Bold data mean statistically significant (*p* < 0.05). OR: odds ratio. CI: confidence interval. N: Number of Studies. HNC: head and neck cancer. OC: oral cancer. LC: laryngeal cancer. NPC: Nasopharyngeal cancer. HB: hospital-based. PB: population-based.

**Table 4:** Random-effect meta-regression analysis.

Polymorphism (N)	Variable	Model	Coefficient	Standard Error	95% Lower	95% Upper	Z-Value	*p*-Value
*rs17655* (12)	Publication year	C vs. G	−0.0005	0.0003	−0.0011	0.0001	−1.57	0.1171
		CC vs. GG	−0.0005	0.0006	−0.0016	0.0006	−0.93	0.3518
		GC vs. GG	0.0003	0.0010	−0.0017	0.0023	0.28	0.7816
		CC + GC vs. GG	−0.0007	0.0014	−0.0033	0.0020	−0.50	0.6152
		CC vs. GG + GC	−0.0005	0.0004	−0.0014	0.0003	−1.18	0.2369
	Sample size	C vs. G	0.0001	0.0001	−0.0001	0.0002	0.66	0.5112
		CC vs. GG	0.0001	0.0002	−0.0002	0.0005	0.66	0.5084
		GC vs. GG	0.0002	0.0003	−0.0004	0.0009	0.72	0.4708
		CC + GC vs. GG	0.0002	0.0004	−0.0007	0.0011	0.40	0.6867
		CC vs. GG + GC	< 0.0001	0.0001	−0.0003	0.0003	0.22	0.8232
	Quality score	C vs. G	0.1013	0.0790	−0.0535	0.2561	1.28	0.1995
		CC vs. GG	0.0961	0.1522	−0.2022	0.3944	0.63	0.5278
		GC vs. GG	−0.0671	0.2663	−0.5890	0.4548	−0.25	0.8010
		CC + GC vs. GG	0.1956	0.3539	−0.4971	0.8902	0.56	0.5786
		CC vs. GG + GC	0.1062	0.1169	−0.1229	0.3352	0.91	0.3636
*rs1800975* (8)	Publication year	A vs. G	−0.0006	0.0018	−0.0014	0.0030	−0.32	0.7519
		AA vs. GG	−0.0011	0.0010	−0.0031	0.0010	−1.04	0.2948
		GA vs. GG	−0.0007	0.0009	−0.0025	0.0012	−0.71	0.4793
		AA + GA vs. GG	−0.0008	0.0008	−0.0024	0.0007	−1.03	0.3045
		AA vs. GG + GA	−0.0010	0.0025	−0.0059	0.0039	−0.40	0.6859
	Sample size	A vs. G	−0.0005	0.0007	−0.0018	0.0009	−0.67	0.5021
		AA vs. GG	−0.0002	0.0003	−0.0008	0.0004	−0.70	0.4845
		GA vs. GG	−0.0003	0.0003	−0.0009	0.0004	−0.83	0.4070
		AA + GA vs. GG	−0.0002	0.0003	−0.0007	0.0003	−0.78	0.4328
		AA vs. GG + GA	−0.0006	0.0009	−0.0024	0.0012	−0.66	0.5120
	Quality score	A vs. G	0.1647	0.5359	−0.8830	1.2178	0.31	0.7548
		AA vs. GG	0.2972	0.2988	−0.2883	0.8828	0.99	0.3198
		GA vs. GG	0.2057	0.2717	−0.3268	0.7381	0.76	0.4490
		AA + GA vs. GG	0.2352	0.2339	−0.2232	0.6936	1.01	0.3145
		AA vs. GG + GA	0.2765	0.7316	−1.1574	1.7104	0.38	0.7055

N: Number of studies.

**Table 5:** Pooled analysis for the polymorphisms to remove the studies with a deviation of Hardy–Weinberg equilibrium in controls.

Polymorphism (Number of Studies without a Deviation)	Genetic Model	OR	95%CI		Z-Value	*p*-Value	I^2^	*P*_heterogeneity_
			Min.	Max.				
*rs17655* (5)	C vs. G	0.99	0.90	1.09	0.25	0.81	0%	0.85
	CC vs. GG	0.95	0.75	1.21	0.42	0.68	0%	0.69
	GC vs. GG	1.03	0.89	1.20	0.42	0.68	0%	0.59
	CC + GC vs. GG	1.00	0.87	1.15	0.01	0.99	30%	0.22
	CC vs. GG + GC	0.96	0.82	1.12	0.54	0.59	0%	0.89
*rs1800975* (4)	A vs. G	0.62	0.26	1.49	1.08	0.28	98%	<0.00001
	AA vs. GG	0.87	0.68	1.12	1.08	0.28	29%	0.24
	GA vs. GG	0.84	0.63	1.12	1.20	0.23	51%	0.11
	AA + GA vs. GG	0.84	0.63	1.11	1.22	0.22	55%	0.08
	AA vs. GG + GA	0.49	0.15	1.63	1.16	0.25	96%	<0.00001

OR: odds ratio. CI: confidence interval.

## Data Availability

All data obtained were included in this article.

## Supplementary Materials

The following supporting information can be downloaded at: https://www.mdpi.com/article/10.3390/medicina60030478/s1, Supplementary File S1—Figures S1–S50: Forest plot analyses; Supplementary File S2—Figures S1–S10: Trial sequential analyses; Supplementary File S3—Figures S1–S20: Funnel plots.

## References

[1] Barsouk A., Aluru J.S., Rawla P., Saginala K., Barsouk A. (2023). Epidemiology, Risk Factors, and Prevention of Head and Neck Squamous Cell Carcinoma. Med. Sci..

[2] Dhull A.K., Atri R., Dhankhar R., Chauhan A.K., Kaushal V. (2018). Major risk factors in head and neck cancer: A retrospective analysis of 12-year experiences. World J. Oncol..

[3] Yang T.-H., Xirasagar S., Cheng Y.-F., Chen C.-S., Chang W.-P., Lin H.-C. (2023). Trends in the incidence of head and neck cancer: A nationwide population-based study. Oral Oncol..

[4] Machiels J.-P., Leemans C.R., Golusinski W., Grau C., Licitra L., Gregoire V. (2020). Squamous cell carcinoma of the oral cavity, larynx, oropharynx and hypopharynx: EHNS–ESMO–ESTRO Clinical Practice Guidelines for diagnosis, treatment and follow-up. Ann. Oncol..

[5] Lacko M., Braakhuis B.J., Sturgis E.M., Boedeker C.C., Suárez C., Rinaldo A., Ferlito A., Takes R.P. (2014). Genetic susceptibility to head and neck squamous cell carcinoma. Int. J. Radiat. Oncol. Biol. Phys..

[6] Mohammadi H., Momeni Roochi M., Rezaei F., Garajei A., Heidar H., Ghaderi B., Sadeghi M. (2022). Association between the CYP1A1 MspI polymorphism and risk of head and neck cancer: A meta-analysis. Sci. Rep..

[7] Mohammadi H., Roochi M.M., Sadeghi M., Garajei A., Heidar H., Ghaderi B., Tadakamadla J., Meybodi A.A., Dallband M., Mostafavi S. (2021). Association of N-acetyltransferases 1 and 2 Polymorphisms with Susceptibility to Head and Neck Cancers-A Meta-Analysis, Meta-Regression, and Trial Sequential Analysis. Medicina.

[8] Mozaffari H.R., Rostamnia M., Sharifi R., Safaei M., Zavattaro E., Tadakamadla S.K., Imani M.M., Sadeghi M., Golshah A., Moradpoor H. (2021). A PRISMA-compliant meta-analysis on association between X-ray repair cross complementing (XRCC1, XRCC2, and XRCC3) polymorphisms and oral cancer susceptibility. Gene.

[9] Rezaei F., Mohammadi H., Heydari M., Sadeghi M., Mozaffari H.R., Khavid A., Godiny M., Brand S., Dürsteler K.M., Beatrix Brühl A. (2021). Association between IL-8 (-251T/A) and IL-6 (-174G/C) Polymorphisms and Oral Cancer Susceptibility: A Systematic Review and Meta-Analysis. Medicina.

[10] Lee T.-H., Kang T.-H. (2019). DNA oxidation and excision repair pathways. Int. J. Mol. Sci..

[11] Huang R., Zhou P.-K. (2021). DNA damage repair: Historical perspectives, mechanistic pathways and clinical translation for targeted cancer therapy. Signal Transduct. Target. Ther..

[12] Kokic G., Chernev A., Tegunov D., Dienemann C., Urlaub H., Cramer P. (2019). Structural basis of TFIIH activation for nucleotide excision repair. Nat. Commun..

[13] Okamura K., Toyoda M., Hata K., Nakabayashi K., Umezawa A. (2015). Whole-exome sequencing of fibroblast and its iPS cell lines derived from a patient diagnosed with xeroderma pigmentosum. Genom. Data.

[14] Zebian A., Shaito A., Mazurier F., Rezvani H.R., Zibara K. (2019). XPC beyond nucleotide excision repair and skin cancers. Mutat. Res. Rev. Mutat. Res..

[15] Bootsma D., Hoeijmakers J.H. (1991). The genetic basis of xeroderma pigmentosum. Ann. Genet..

[16] Nagaria P., Robert C., Rassool F.V. (2013). DNA double-strand break response in stem cells: Mechanisms to maintain genomic integrity. Biochim. Biophys. Acta (BBA)-Gen. Subj..

[17] Kumar N., Moreno N.C., Feltes B.C., Menck C.F., Houten B.V. (2020). Cooperation and interplay between base and nucleotide excision repair pathways: From DNA lesions to proteins. Genet. Mol. Biol..

[18] Tse D., Zhai R., Zhou W., Heist R.S., Asomaning K., Su L., Lynch T.J., Wain J.C., Christiani D.C., Liu G. (2008). Polymorphisms of the NER pathway genes, ERCC1 and XPD are associated with esophageal adenocarcinoma risk. Cancer Causes Control.

[19] Machado C.R., Vieira-da-Rocha J.P., Mendes I.C., Rajão M.A., Marcello L., Bitar M., Drummond M.G., Grynberg P., Oliveira D.A., Marques C. (2014). Nucleotide excision repair in T rypanosoma brucei: Specialization of transcription-coupled repair due to multigenic transcription. Mol. Microbiol..

[20] McCullough L.E., Santella R.M., Cleveland R.J., Millikan R.C., Olshan A.F., North K.E., Bradshaw P.T., Eng S.M., Terry M.B., Shen J. (2014). Polymorphisms in DNA repair genes, recreational physical activity and breast cancer risk. Int. J. Cancer.

[21] Jiang H.-Y., Zeng Y., Xu W.-D., Liu C., Wang Y.-J., Wang Y.-D. (2015). Genetic association between the XPG Asp1104His polymorphism and head and neck cancer susceptibility: Evidence based on a meta-analysis. Asian Pac. J. Cancer Prev..

[22] Li T., Ling H., Lu Y., Wu X., Cai M., Su B., Zou Y. (2018). Meta-analysis of the relationship between excision repair cross-complementing Group 5 rs17655 gene polymorphism and head and neck cancer susceptibility. J. Cancer Res. Ther..

[23] Wu L., Gao X., Ye D., Ding Y., Yang X., Liu W. (2015). Association of the XPA A23G polymorphism with the risk of head and neck carcinomas: Evidence from 5491 subjects. Mol. Clin. Oncol..

[24] Li X., Xu J., Yang X., Wu Y., Cheng B., Chen D., Bai B. (2014). Association of single nucleotide polymorphisms of nucleotide excision repair genes with laryngeal cancer risk and interaction with cigarette smoking and alcohol drinking. Tumor Biol..

[25] Avci H., Iplik E.S., Aydemir L., Acar S., Kiyak E., Ünür M., Cakmakoglu B. (2018). Are XPD and XPG gene variants related to the mechanism of oral squamous cell carcinoma?. Cell. Mol. Biol..

[26] Hall J., Hashibe M., Boffetta P., Gaborieau V., Moullan N., Chabrier A., Zaridze D., Shangina O., Szeszenia-Dabrowska N., Mates D. (2007). The association of sequence variants in DNA repair and cell cycle genes with cancers of the upper aerodigestive tract. Carcinogenesis.

[27] Lu B., Li J., Gao Q., Yu W., Yang Q., Li X. (2014). Laryngeal cancer risk and common single nucleotide polymorphisms in nucleotide excision repair pathway genes ERCC1, ERCC2, ERCC3, ERCC4, ERCC5 and XPA. Gene.

[28] Page M.J., McKenzie J.E., Bossuyt P.M., Boutron I., Hoffmann T.C., Mulrow C.D., Shamseer L., Tetzlaff J.M., Akl E.A., Brennan S.E. (2021). The PRISMA 2020 statement: An updated guideline for reporting systematic reviews. Int. J. Surg..

[29] Peterson J., Welch V., Losos M., Tugwell P. (2011). The Newcastle-Ottawa scale (NOS) for assessing the quality of nonrandomised studies in meta-analyses. Ott. Ott. Hosp. Res. Inst..

[30] DerSimonian R., Laird N. (2015). Meta-analysis in clinical trials revisited. Contemp. Clin. Trials.

[31] Mantel N., Haenszel W. (1959). Statistical aspects of the analysis of data from retrospective studies of disease. J. Natl. Cancer Inst..

[32] Imberger G., Thorlund K., Gluud C., Wetterslev J. (2016). False-positive findings in Cochrane meta-analyses with and without application of trial sequential analysis: An empirical review. BMJ Open.

[33] Wetterslev J., Jakobsen J.C., Gluud C. (2017). Trial sequential analysis in systematic reviews with meta-analysis. BMC Med. Res. Methodol..

[34] Abbasi R., Ramroth H., Becher H., Dietz A., Schmezer P., Popanda O. (2009). Laryngeal cancer risk associated with smoking and alcohol consumption is modified by genetic polymorphisms in ERCC5, ERCC6 and RAD23B but not by polymorphisms in five other nucleotide excision repair genes. Int. J. Cancer.

[35] An J., Liu Z., Hu Z., Li G., Wang L.-E., Sturgis E.M., El-Naggar A.K., Spitz M.R., Wei Q. (2007). Potentially functional single nucleotide polymorphisms in the core nucleotide excision repair genes and risk of squamous cell carcinoma of the head and neck. Cancer Epidemiol. Biomark. Prev..

[36] Bau D., Tsai M., Huang C., Lee C., Tseng H., Lo Y., Tsai Y., Tsai F. (2007). Relationship between polymorphisms of nucleotide excision repair genes and oral cancer risk in Taiwan: Evidence for modification of smoking habit. Chin. J. Physiol..

[37] Cui Y., Morgenstern H., Greenland S., Tashkin D.P., Mao J., Cao W., Cozen W., Mack T.M., Zhang Z.F. (2006). Polymorphism of Xeroderma Pigmentosum group G and the risk of lung cancer and squamous cell carcinomas of the oropharynx, larynx and esophagus. Int. J. Cancer.

[38] Jelonek K., Gdowicz-Kłosok A., Pietrowska M., Borkowska M., Korfanty J., Rzeszowska-Wolny J., Widłak P. (2010). Association between single-nucleotide polymorphisms of selected genes involved in the response to DNA damage and risk of colon, head and neck, and breast cancers in a Polish population. J. Appl. Genet..

[39] Ma H., Yu H., Liu Z., Wang L.-E., Sturgis E.M., Wei Q. (2012). Polymorphisms of XPG/ERCC5 and risk of squamous cell carcinoma of the head and neck. Pharmacogenet. Genom..

[40] Nigam K., Yadav S.K., Samadi F.M., Bhatt M.L., Gupta S., Sanyal S. (2019). Risk modulation of oral pre cancer and cancer with polymorphisms in XPD and XPG genes in North Indian population. Asian Pac. J. Cancer Prev. APJCP.

[41] Sugimura T., Kumimoto H., Tohnai I., Fukui T., Matsuo K., Tsurusako S., Mitsudo K., Ueda M., Tajima K., Ishizaki K. (2006). Gene–environment interaction involved in oral carcinogenesis: Molecular epidemiological study for metabolic and DNA repair gene polymorphisms. J. Oral Pathol. Med..

[42] Sun Y., Tan L., Li H., Qin X., Liu J. (2015). Association of NER pathway gene polymorphisms with susceptibility to laryngeal cancer in a Chinese population. Int. J. Clin. Exp. Pathol..

[43] Wen S.X., Tang P.Z., Zhang X.M., Zhao D., Guo Y.L., Tan W., Lin D.X. (2006). Association between genetic polymorphism in xeroderma pigmentosum G gene and risks of laryngeal and hypopharyngeal carcinomas. Zhongguo Yi Xue Ke Xue Yuan Xue Bao. Acta Acad. Med. Sin..

[44] Xue M., Qiu-xu W., Mo-ye C., Gang Q., Yu-peng W. (2013). DNA repair gene polymorphisms in ERCC4 rs6498486 and ERCC5 rs751402 and risk of salivary gland tumors. Shanghai J. Stomatol..

[45] Yuan H., Li H., Ma H., Niu Y., Wu Y., Zhang S., Hu Z., Shen H., Chen N. (2012). Genetic polymorphisms in key DNA repair genes and risk of head and neck cancer in a Chinese population. Exp. Ther. Med..

[46] Zavras A.I., Yoon A.J., Chen M.-K., Lin C.-W., Yang S.-F. (2012). Association between polymorphisms of DNA repair gene ERCC5 and oral squamous cell carcinoma. Oral Surg. Oral Med. Oral Pathol. Oral Radiol..

[47] Zhu Y., Guo L., Wang S., Yu Q., Lu J. (2018). Association of Smoking and XPG, CYP1A1, OGG1, ERCC5, ERCC1, MMP2, and MMP9 Gene Polymorphisms with the early detection and occurrence of Laryngeal Squamous Carcinoma. J. Cancer.

[48] Yu H., Liu Z., Huang Y.J., Yin M., Wang L.E., Wei Q. (2012). Association between single nucleotide polymorphisms in ERCC4 and risk of squamous cell carcinoma of the head and neck. PLoS ONE.

[49] Kamileri I., Karakasilioti I., Garinis G.A. (2012). Nucleotide excision repair: New tricks with old bricks. Trends Genet..

[50] Wood R.D., Mitchell M., Lindahl T. (2005). Human DNA repair genes, 2005. Mutat. Res./Fundam. Mol. Mech. Mutagen..

[51] Petruseva I., Evdokimov A., Lavrik O. (2014). Molecular mechanism of global genome nucleotide excision repair. Acta Nat..

[52] Liu J., He C., Xing C., Yuan Y. (2014). Nucleotide excision repair related gene polymorphisms and genetic susceptibility, chemotherapeutic sensitivity and prognosis of gastric cancer. Mutat. Res./Fundam. Mol. Mech. Mutagen..

[53] Xue M.-H., Li G.-Y., Wu X.-J., Zhang C.-X., Zhang C.-F., Zhu K.-X. (2015). Genetic variability of genes in NER pathway influences the treatment outcome of gastric cancer. Int. J. Clin. Exp. Pathol..

[54] Shuck S.C., Short E.A., Turchi J.J. (2008). Eukaryotic nucleotide excision repair: From understanding mechanisms to influencing biology. Cell Res..

[55] d’Errico M., Parlanti E., Teson M., de Jesus B.M.B., Degan P., Calcagnile A., Jaruga P., Bjørås M., Crescenzi M., Pedrini A.M. (2006). New functions of XPC in the protection of human skin cells from oxidative damage. EMBO J..

[56] Niedernhofer L.J., Odijk H., Budzowska M., Van Drunen E., Maas A., Theil A.F., De Wit J., Jaspers N., Beverloo H.B., Hoeijmakers J.H. (2004). The structure-specific endonuclease Ercc1-Xpf is required to resolve DNA interstrand cross-link-induced double-strand breaks. Mol. Cell. Biol..

[57] Nagai A., Saijo M., Kuraoka I., Matsuda T., Kodo N., Nakatsu Y., Mimaki T., Mino M., Biggerstaff M., Wood R.D. (1995). Enhancement of damage-specific DNA binding of XPA by interaction with the ERCC1 DNA repair protein. Biochem. Biophys. Res. Commun..

[58] Nocentini S., Coin F., Saijo M., Tanaka K., Egly J.-M. (1997). DNA damage recognition by XPA protein promotes efficient recruitment of transcription factor II H. J. Biol. Chem..

[59] Patrick S.M., Turchi J.J. (2002). Xeroderma pigmentosum complementation group A protein (XPA) modulates RPA-DNA interactions via enhanced complex stability and inhibition of strand separation activity. J. Biol. Chem..

[60] Tudek B., Winczura A., Janik J., Siomek A., Foksinski M., Oliński R. (2010). Involvement of oxidatively damaged DNA and repair in cancer development and aging. Am. J. Transl. Res..

[61] Liu J., Zhang Z., Cao X.-L., Lei D.-P., Wang Z.-Q., Jin T., Pan X.-L. (2012). XPA A23G polymorphism and susceptibility to cancer: A meta-analysis. Mol. Biol. Rep..

[62] Huang J., Liu X., Tang L.-L., Long J.-T., Zhu J., Hua R.-X., Li J. (2017). XPG gene polymorphisms and cancer susceptibility: Evidence from 47 studies. Oncotarget.

[63] Rodrigo J.P., Suárez C., González M.V., Lazo P.S., Ramos S., Coto E., Alvarez I., García L.A., Martínez J.A. (2001). Variability of genetic alterations in different sites of head and neck cancer. Laryngoscope.

