# Supplementary material for: Relationship between XPA, XPB/ERCC3, XPF/ERCC4, and XPG/ERCC5 Polymorphisms and the Susceptibility to Head and Neck Carcinoma: A Systematic Review, Meta-Analysis, and Trial Sequential Analysis
Source: Medicina (Kaunas). 2024 Mar 14;60(3):478. doi: 10.3390/medicina60030478 (PMC10972270; doi:10.3390/medicina60030478)
Supplement: Supplementary file 1 [file medicina-60-00478-s001.zip › Supplementary File S1.pdf]

Study or Subgroup	Case Events	Case Total	Control Events	Control Total	Weight	Odds Ratio M-H, Random, 95% CI
Abbasi, 2009	119	496	304	1294	8.5%	1.03 [0.81, 1.31]
An,2007	1300	1658	1327	1708	11.5%	1.04 [0.89, 1.23]
Avci,2018	58	222	79	296	4.7%	0.97 [0.65, 1.44]
Cui,2006	622	886	1035	1552	10.8%	1.18 [0.98, 1.41]
Li,2014	207	422	249	420	7.5%	0.66 [0.50, 0.87]
Lu, 2014	175	352	218	352	6.8%	0.61 [0.45, 0.82]
Ma,2012	463	2118	474	2132	12.2%	0.98 [0.85, 1.13]
Nigam,2019	41	134	203	576	4.6%	0.81 [0.54, 1.21]
Sugimura,2006	145	244	266	482	6.4%	1.19 [0.87, 1.63]
Wen,2006	159	350	496	1050	8.5%	0.93 [0.73, 1.19]
Yuan,2012	381	788	867	1768	11.3%	0.97 [0.82, 1.15]
Zhu,2018	192	398	186	380	7.3%	0.97 [0.73, 1.29]
**Total (95% CI)**		**8068**		**12010**	**100.0%**	**0.95 [0.86, 1.05]**
Total events	3862		5704			

Heterogeneity: Tau² = 0.02; Chi² = 24.93, df = 11 (P = 0.009); I² = 56%
Test for overall effect: Z = 1.03 (P = 0.30)

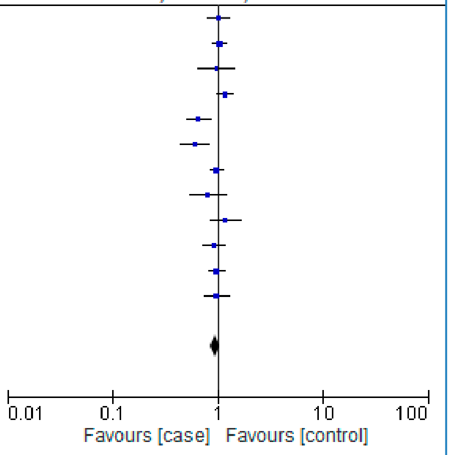

Figure S1: Forest plot of association between *rs17655* polymorphism and the risk of head and neck cancer in allelic model

Study or Subgroup	Case Events	Case Total	Control Events	Control Total	Weight	Odds Ratio M-H, Fixed, 95% CI
Abbasi, 2009	8	145	37	417	4.5%	0.60 [0.27, 1.32]
An,2007	507	543	519	565	8.4%	1.25 [0.79, 1.96]
Avci,2018	7	67	11	91	2.1%	0.85 [0.31, 2.32]
Cui,2006	214	249	474	554	10.3%	1.03 [0.67, 1.58]
Li,2014	64	132	88	137	11.1%	0.52 [0.32, 0.85]
Lu, 2014	53	107	78	114	9.5%	0.45 [0.26, 0.78]
Ma,2012	52	700	62	716	14.2%	0.85 [0.58, 1.24]
Nigam,2019	7	40	37	159	3.1%	0.70 [0.29, 1.71]
Sugimura,2006	43	63	77	129	4.0%	1.45 [0.77, 2.74]
Wen,2006	39	94	100	229	8.5%	0.91 [0.56, 1.49]
Yuan,2012	95	203	217	451	17.9%	0.95 [0.68, 1.32]
Zhu,2018	45	97	46	96	6.2%	0.94 [0.53, 1.66]
**Total (95% CI)**		**2440**		**3658**	**100.0%**	**0.86 [0.75, 1.00]**
Total events	1134		1746			

Heterogeneity: Chi² = 16.61, df = 11 (P = 0.12); I² = 34%
Test for overall effect: Z = 1.98 (P = 0.05)

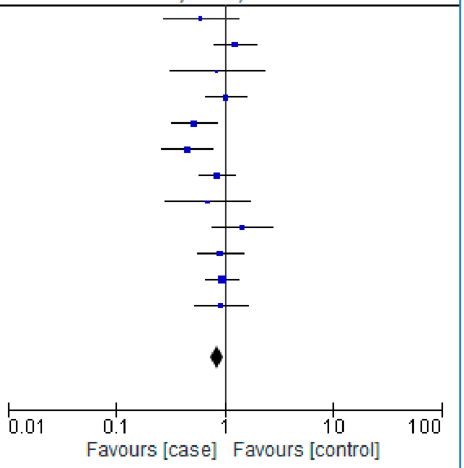

Figure S2: Forest plot of association between *rs17655* polymorphism and the risk of head and neck cancer in homozygous model

Study or Subgroup	Case Events	Case Total	Control Events	Control Total	Weight	Odds Ratio M-H, Random, 95% CI
Abbasi, 2009	103	240	103	483	9.1%	2.77 [1.98, 3.88]
An,2007	286	322	286	332	8.2%	1.28 [0.80, 2.04]
Avci,2018	44	104	44	124	7.8%	1.33 [0.78, 2.28]
Cui,2006	194	229	194	274	8.4%	2.29 [1.47, 3.56]
Li,2014	79	147	79	128	8.1%	0.72 [0.45, 1.17]
Lu, 2014	69	123	62	98	7.7%	0.74 [0.43, 1.28]
Ma,2012	359	1007	359	1013	9.9%	1.01 [0.84, 1.21]
Nigam,2019	27	60	129	251	7.5%	0.77 [0.44, 1.36]
Sugimura,2006	59	79	59	111	7.1%	2.60 [1.39, 4.88]
Wen,2006	81	136	296	425	8.7%	0.64 [0.43, 0.96]
Yuan,2012	191	299	191	425	9.3%	2.17 [1.60, 2.94]
Zhu,2018	102	154	102	152	8.2%	0.96 [0.60, 1.55]
**Total (95% CI)**		**2900**		**3816**	**100.0%**	**1.26 [0.94, 1.71]**
Total events	1594		1904			

Heterogeneity: Tau² = 0.23; Chi² = 75.03, df = 11 (P < 0.00001); I² = 85%
Test for overall effect: Z = 1.53 (P = 0.13)

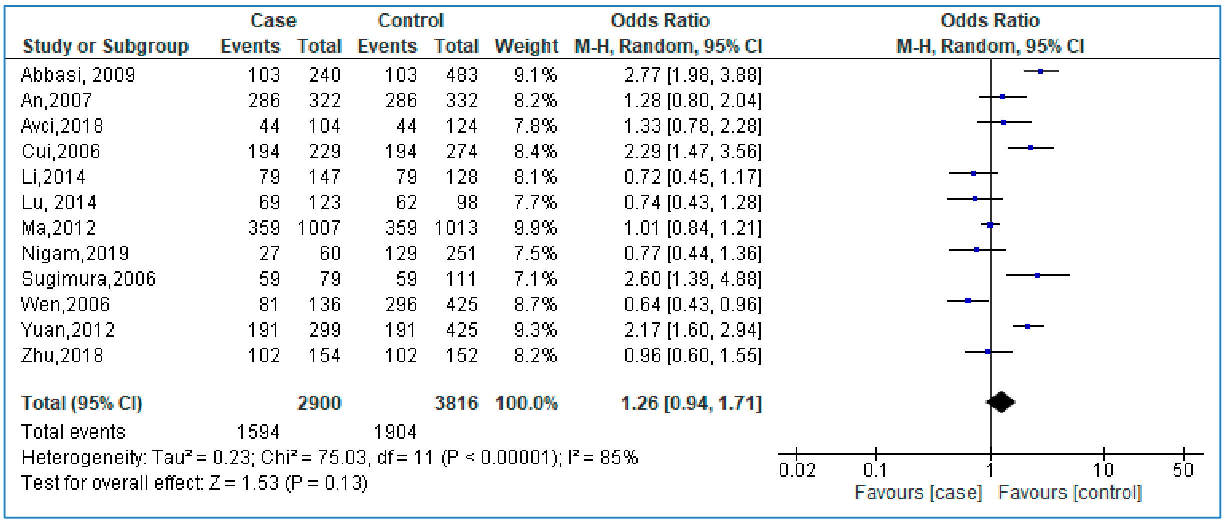

Figure S3: Forest plot of association between *rs17655* polymorphism and the risk of head and neck cancer in heterozygous model

Study or Subgroup	Case Events	Case Total	Control Events	Control Total	Weight	Odds Ratio M-H, Random, 95% CI
Abbasi, 2009	111	248	140	647	8.7%	2.93 [2.15, 4.01]
An,2007	793	829	805	854	8.2%	1.34 [0.86, 2.08]
Avci,2018	51	111	55	148	8.0%	1.44 [0.87, 2.37]
Cui,2006	408	443	668	911	8.5%	4.24 [2.91, 6.17]
Li,2014	143	211	167	210	8.2%	0.54 [0.35, 0.84]
Lu, 2014	122	176	140	176	8.1%	0.58 [0.36, 0.95]
Ma,2012	411	1059	421	1066	9.0%	0.97 [0.82, 1.16]
Nigam,2019	34	67	166	288	7.9%	0.76 [0.44, 1.29]
Sugimura,2006	102	122	136	241	7.8%	3.94 [2.29, 6.78]
Wen,2006	120	175	296	525	8.5%	1.69 [1.17, 2.43]
Yuan,2012	286	394	408	884	8.8%	3.09 [2.39, 4.00]
Zhu,2018	147	199	148	190	8.2%	0.80 [0.50, 1.28]
**Total (95% CI)**		**4034**		**6140**	**100.0%**	**1.47 [0.98, 2.19]**
Total events	2728		3550			

Heterogeneity: Tau² = 0.45; Chi² = 162.55, df = 11 (P < 0.00001); I² = 93%
Test for overall effect: Z = 1.88 (P = 0.06)

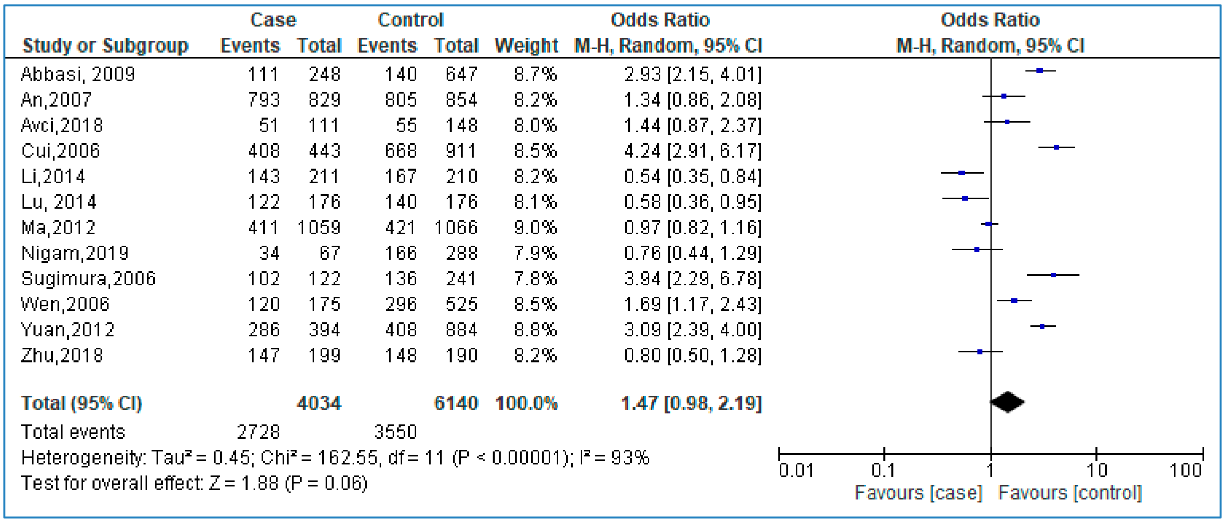

Figure S4: Forest plot of association between *rs17655* polymorphism and the risk of head and neck cancer in dominant model

Study or Subgroup	Case Events	Case Total	Control Events	Control Total	Weight	Odds Ratio M-H, Fixed, 95% CI
Abbasi, 2009	8	248	37	647	2.5%	0.55 [0.25, 1.20]
An,2007	507	829	519	854	25.3%	1.02 [0.84, 1.24]
Avci,2018	7	111	11	148	1.1%	0.84 [0.31, 2.24]
Cui,2006	214	443	474	911	20.4%	0.86 [0.69, 1.08]
Li,2014	64	211	88	210	7.8%	0.60 [0.40, 0.90]
Lu, 2014	53	176	78	176	6.9%	0.54 [0.35, 0.84]
Ma,2012	52	1059	62	1066	7.5%	0.84 [0.57, 1.22]
Nigam,2019	7	67	37	288	1.6%	0.79 [0.34, 1.86]
Sugimura,2006	43	122	77	241	4.3%	1.16 [0.73, 1.84]
Wen,2006	39	175	100	525	4.9%	1.22 [0.80, 1.85]
Yuan,2012	95	394	217	884	12.9%	0.98 [0.74, 1.29]
Zhu,2018	45	199	46	190	4.6%	0.91 [0.57, 1.46]
**Total (95% CI)**		**4034**		**6140**	**100.0%**	**0.89 [0.81, 0.99]**
Total events	1134		1746			

Heterogeneity: Chi² = 15.91, df = 11 (P = 0.14); I² = 31%
Test for overall effect: Z = 2.14 (P = 0.03)

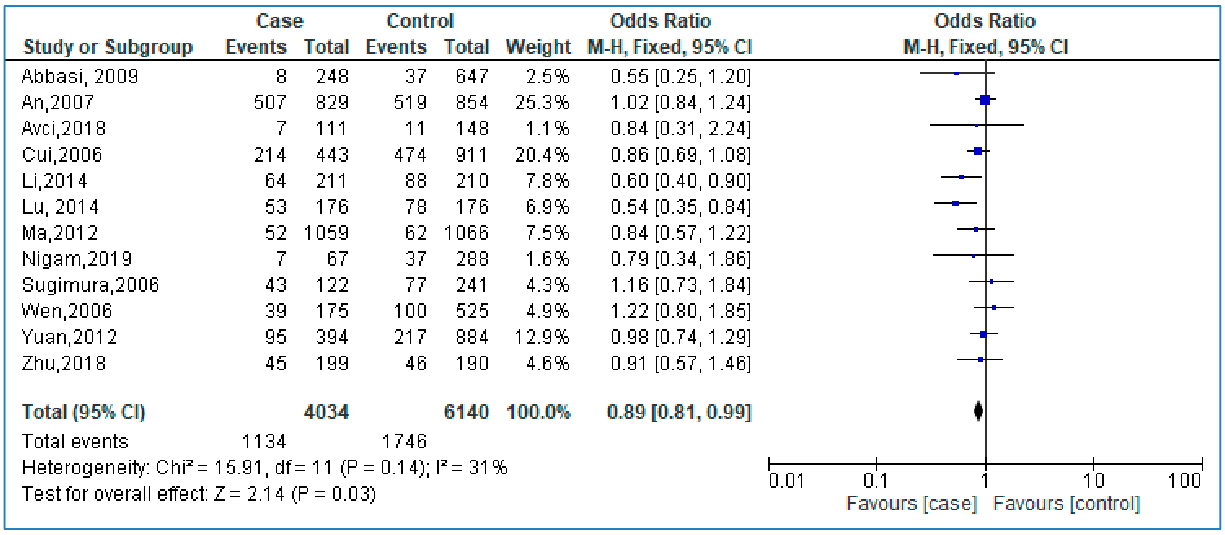

Figure S5: Forest plot of association between *rs17655* polymorphism and the risk of head and neck cancer in recessive model

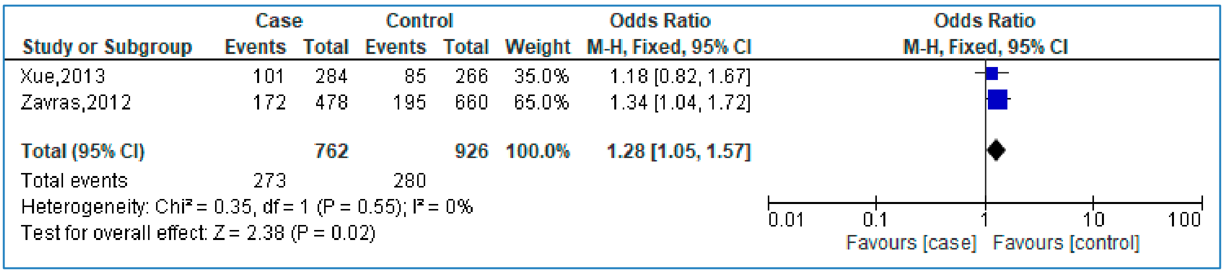

Figure S6: Forest plot of association between *rs751402* polymorphism and the risk of head and neck cancer in allelic model

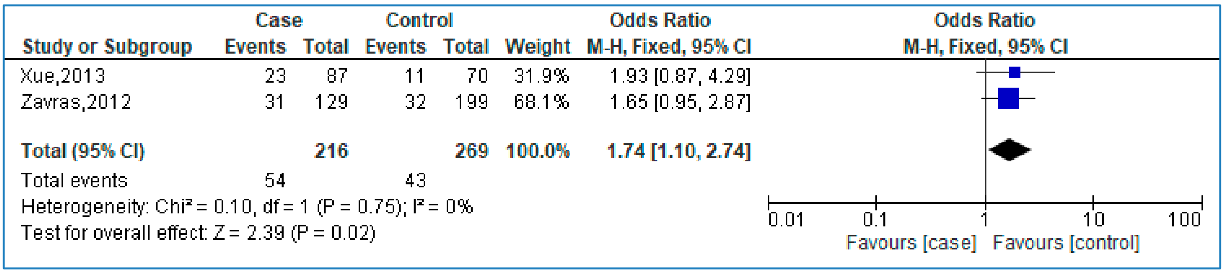

Figure S7: Forest plot of association between *rs751402* polymorphism and the risk of head and neck cancer in homozygous model

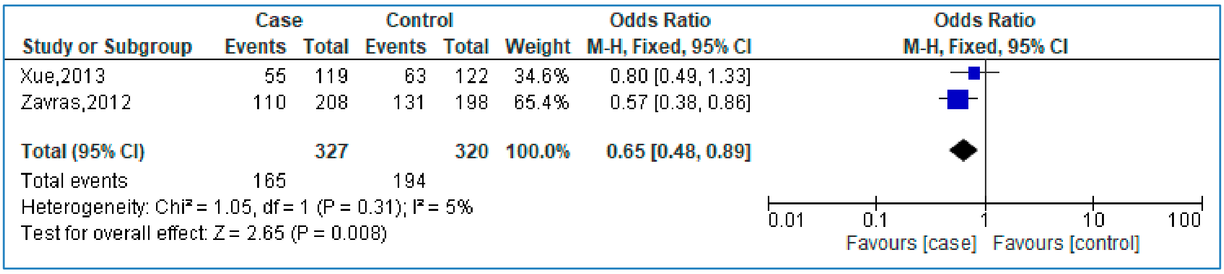

Figure S8: Forest plot of association between *rs751402* polymorphism and the risk of head and neck cancer in heterozygous model

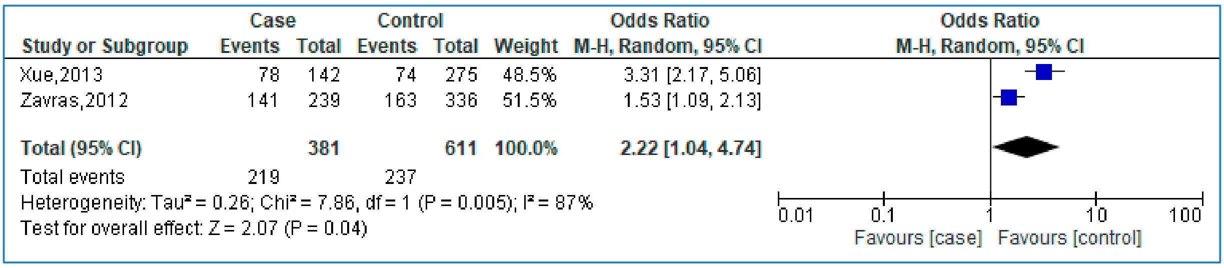

Figure S9: Forest plot of association between *rs751402* polymorphism and the risk of head and neck cancer in dominant model

Study or Subgroup	Case Events	Case Total	Control Events	Control Total	Weight	Odds Ratio M-H, Random, 95% CI
Xue,2013	23	142	11	275	47.3%	4.64 [2.19, 9.82]
Zavras,2012	31	239	32	336	52.7%	1.42 [0.84, 2.39]
**Total (95% CI)**		**381**		**611**	**100.0%**	**2.48 [0.78, 7.93]**
Total events	54		43			

Heterogeneity: Tau² = 0.60; Chi² = 6.46, df = 1 (P = 0.01); I² = 85%

Test for overall effect: Z = 1.53 (P = 0.12)

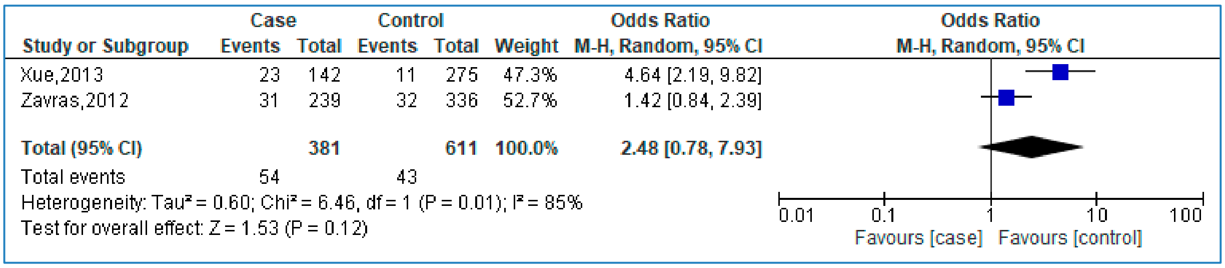

Figure S10: Forest plot of association between *rs751402* polymorphism and the risk of head and neck cancer in recessive model

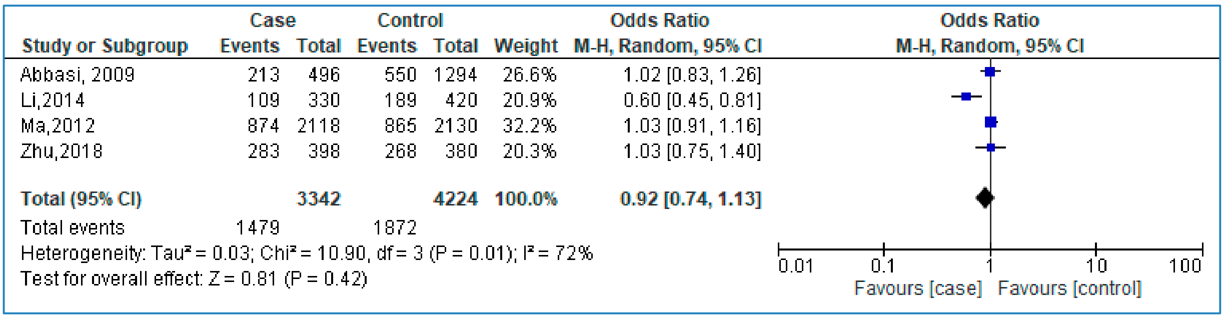

Figure S11: Forest plot of association between *rs1047768* polymorphism and the risk of head and neck cancer in allelic model

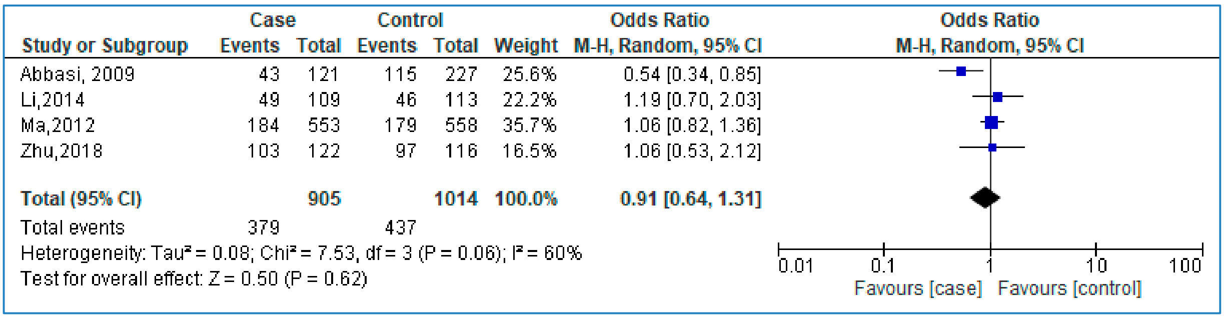

Figure S12: Forest plot of association between *rs1047768* polymorphism and the risk of head and neck cancer in homozygous model

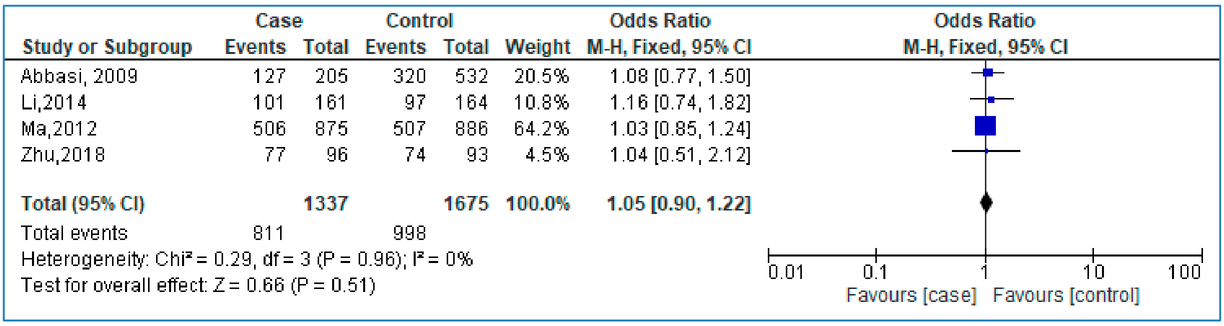

Figure S13: Forest plot of association between *rs1047768* polymorphism and the risk of head and neck cancer in heterozygous model

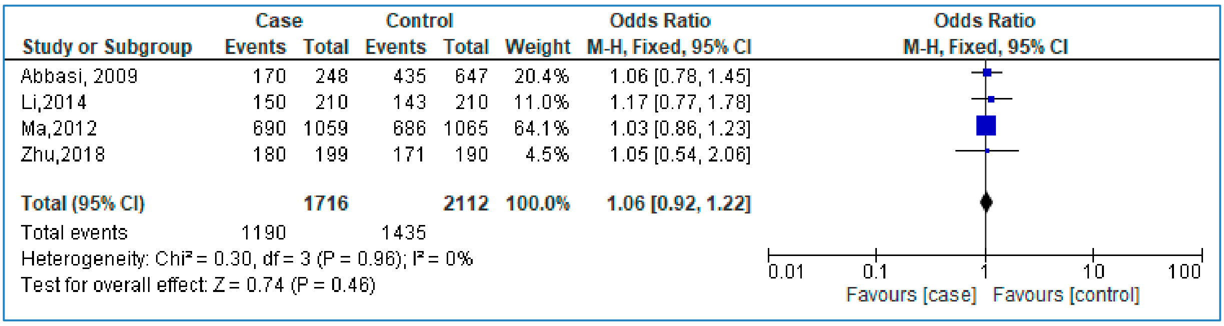

Figure S14: Forest plot of association between *rs1047768* polymorphism and the risk of head and neck cancer in dominant model

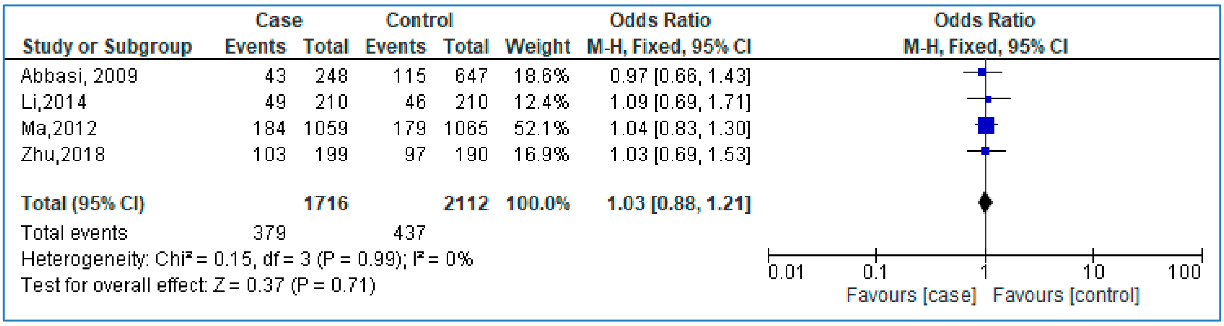

Figure S15: Forest plot of association between *rs1047768* polymorphism and the risk of head and neck cancer in recessive model

Study or Subgroup	Case Events	Case Total	Control Events	Control Total	Weight	Odds Ratio M-H, Fixed, 95% CI
Ma,2012	480	2114	482	2128	82.0%	1.00 [0.87, 1.16]
Zhu,2018	130	398	118	380	18.0%	1.08 [0.80, 1.46]
**Total (95% CI)**		**2512**		**2508**	**100.0%**	**1.02 [0.89, 1.16]**
Total events	610		600			

Heterogeneity: Chi² = 0.17, df = 1 (P = 0.68); I² = 0%
Test for overall effect: Z = 0.25 (P = 0.81)

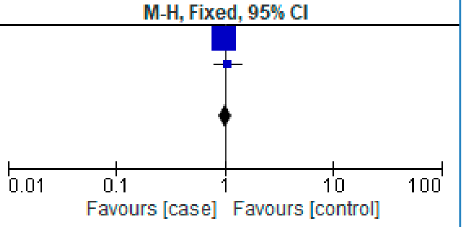

Figure S16: Forest plot of association between *rs4771436* polymorphism and the risk of head and neck cancer in allelic model

Study or Subgroup	Case Events	Case Total	Control Events	Control Total	Weight	Odds Ratio M-H, Fixed, 95% CI
Ma,2012	53	683	55	692	75.6%	0.97 [0.66, 1.44]
Zhu,2018	24	117	20	112	24.4%	1.19 [0.61, 2.30]
**Total (95% CI)**		**800**		**804**	**100.0%**	**1.03 [0.73, 1.44]**
Total events	77		75			

Heterogeneity: Chi² = 0.25, df = 1 (P = 0.61); I² = 0%
Test for overall effect: Z = 0.15 (P = 0.88)

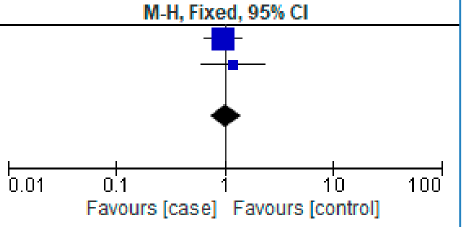

Figure S17: Forest plot of association between *rs4771436* polymorphism and the risk of head and neck cancer in homozygous model

Study or Subgroup	Case Events	Case Total	Control Events	Control Total	Weight	Odds Ratio M-H, Fixed, 95% CI
Ma,2012	374	1004	372	1009	84.7%	1.02 [0.85, 1.22]
Zhu,2018	82	175	78	170	15.3%	1.04 [0.68, 1.59]
**Total (95% CI)**		**1179**		**1179**	**100.0%**	**1.02 [0.86, 1.20]**
Total events	456		450			

Heterogeneity: Chi² = 0.01, df = 1 (P = 0.92); I² = 0%
Test for overall effect: Z = 0.23 (P = 0.81)

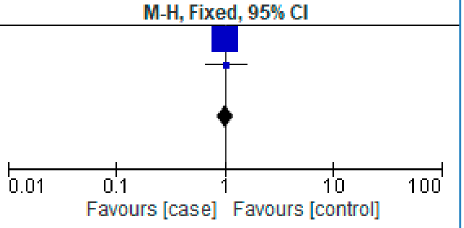

Figure S18: Forest plot of association between *rs4771436* polymorphism and the risk of head and neck cancer in heterozygous model

Study or Subgroup	Case Events	Case Total	Control Events	Control Total	Weight	Odds Ratio M-H, Random, 95% CI
Ma,2012	427	1057	427	1064	50.7%	1.01 [0.85, 1.20]
Zhu,2018	106	199	20	190	49.3%	9.69 [5.64, 16.63]
**Total (95% CI)**		**1256**		**1254**	**100.0%**	**3.08 [0.33, 28.49]**
Total events	533		447			

Heterogeneity: Tau² = 2.53; Chi² = 61.37, df = 1 (P < 0.00001); I² = 98%
Test for overall effect: Z = 0.99 (P = 0.32)

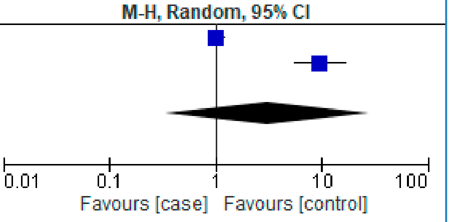

Figure S19: Forest plot of association between *rs4771436* polymorphism and the risk of head and neck cancer in dominant model

Study or Subgroup	Case Events	Case Total	Control Events	Control Total	Weight	Odds Ratio M-H, Fixed, 95% CI
Ma,2012	53	1057	55	1064	74.3%	0.97 [0.66, 1.43]
Zhu,2018	24	199	20	190	25.7%	1.17 [0.62, 2.19]
**Total (95% CI)**		**1256**		**1254**	**100.0%**	**1.02 [0.73, 1.42]**
Total events	77		75			

Heterogeneity: Chi² = 0.24, df = 1 (P = 0.62); I² = 0%
Test for overall effect: Z = 0.11 (P = 0.91)

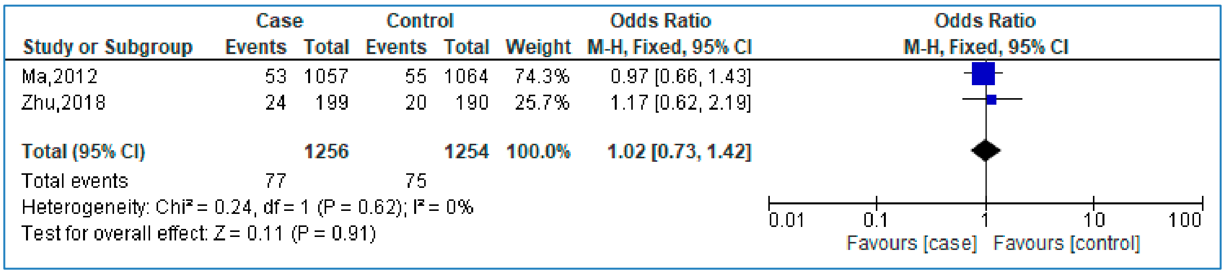

Figure S20: Forest plot of association between *rs4771436* polymorphism and the risk of head and neck cancer in recessive model

Study or Subgroup	Case Events	Case Total	Control Events	Control Total	Weight	Odds Ratio M-H, Fixed, 95% CI
Ma,2012	369	2076	373	2106	75.7%	1.00 [0.86, 1.18]
Sun, 2015	156	542	137	542	24.3%	1.19 [0.91, 1.56]
**Total (95% CI)**		**2618**		**2648**	**100.0%**	**1.05 [0.92, 1.20]**
Total events	525		510			

Heterogeneity: Chi² = 1.19, df = 1 (P = 0.28); I² = 16%
Test for overall effect: Z = 0.71 (P = 0.48)

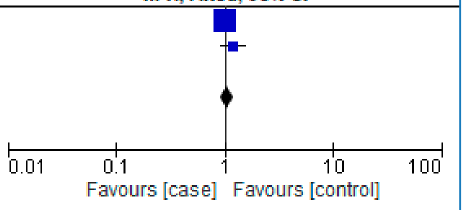

Figure S21: Forest plot of association between *rs2094258* polymorphism and the risk of head and neck cancer in allelic model

Study or Subgroup	Case Events	Case Total	Control Events	Control Total	Weight	Odds Ratio M-H, Fixed, 95% CI
Ma,2012	37	743	41	762	71.9%	0.92 [0.58, 1.45]
Sun, 2015	25	165	18	170	28.1%	1.51 [0.79, 2.88]
**Total (95% CI)**		**908**		**932**	**100.0%**	**1.09 [0.75, 1.57]**
Total events	62		59			

Heterogeneity: Chi² = 1.48, df = 1 (P = 0.22); I² = 33%
Test for overall effect: Z = 0.44 (P = 0.66)

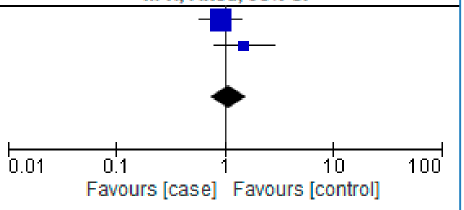

Figure S22: Forest plot of association between *rs2094258* polymorphism and the risk of head and neck cancer in homozygous model

Study or Subgroup	Case Events	Case Total	Control Events	Control Total	Weight	Odds Ratio M-H, Fixed, 95% CI
Ma,2012	295	1001	291	1012	78.3%	1.04 [0.85, 1.25]
Sun, 2015	106	246	101	253	21.7%	1.14 [0.80, 1.63]
**Total (95% CI)**		**1247**		**1265**	**100.0%**	**1.06 [0.89, 1.25]**
Total events	401		392			

Heterogeneity: Chi² = 0.22, df = 1 (P = 0.64); I² = 0%
Test for overall effect: Z = 0.65 (P = 0.51)

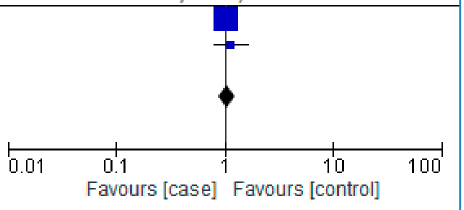

Figure S23: Forest plot of association between *rs2094258* polymorphism and the risk of head and neck cancer in heterozygous model

Study or Subgroup	Case Events	Case Total	Control Events	Control Total	Weight	Odds Ratio M-H, Fixed, 95% CI
Ma,2012	332	1038	332	1053	78.5%	1.02 [0.85, 1.23]
Sun, 2015	131	271	119	271	21.5%	1.20 [0.85, 1.68]
**Total (95% CI)**		**1309**		**1324**	**100.0%**	**1.06 [0.90, 1.24]**
Total events	463		451			

Heterogeneity: Chi² = 0.64, df = 1 (P = 0.42); I² = 0%
Test for overall effect: Z = 0.69 (P = 0.49)

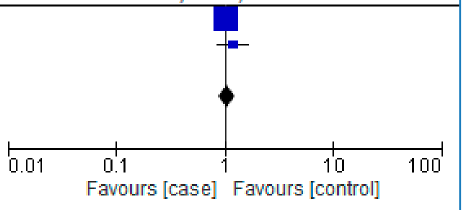

Figure S24: Forest plot of association between *rs2094258* polymorphism and the risk of head and neck cancer in dominant model

Study or Subgroup	Case Events	Case Total	Control Events	Control Total	Weight	Odds Ratio M-H, Fixed, 95% CI
Ma,2012	37	1038	41	1053	70.6%	0.91 [0.58, 1.44]
Sun, 2015	25	271	18	271	29.4%	1.43 [0.76, 2.68]
**Total (95% CI)**		**1309**		**1324**	**100.0%**	**1.06 [0.74, 1.53]**
Total events	62		59			

Heterogeneity: Chi² = 1.28, df = 1 (P = 0.26); I² = 22%

Test for overall effect: Z = 0.33 (P = 0.74)

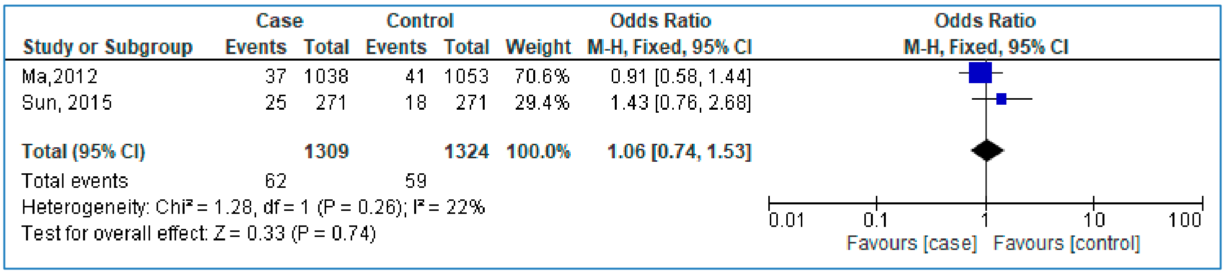

Figure S25: Forest plot of association between *rs2094258* polymorphism and the risk of head and neck cancer in recessive model

Study or Subgroup	Case Events	Case Total	Control Events	Control Total	Weight	Odds Ratio M-H, Fixed, 95% CI
Li,2014	139	420	122	420	41.6%	1.21 [0.90, 1.62]
Lu,2014	117	352	106	352	36.1%	1.16 [0.84, 1.59]
Xue, 2013	62	284	54	266	22.2%	1.10 [0.73, 1.65]
**Total (95% CI)**		**1056**		**1038**	**100.0%**	**1.16 [0.96, 1.41]**
Total events	318		282			
Heterogeneity: Chi² = 0.15, df = 2 (P = 0.93); I² = 0%						
Test for overall effect: Z = 1.56 (P = 0.12)						

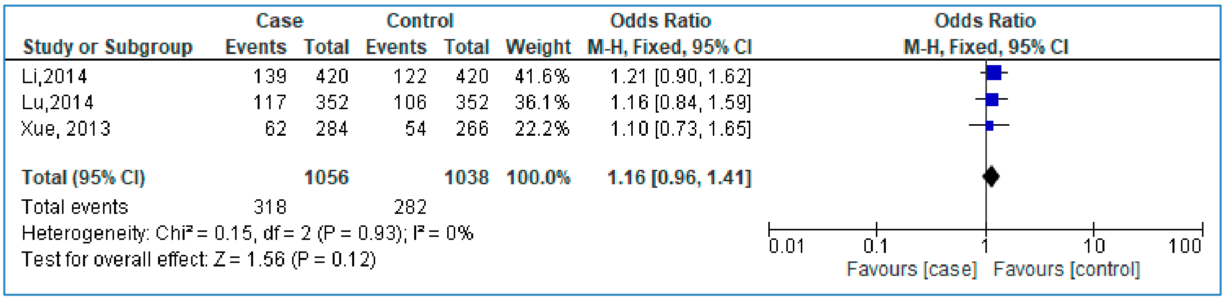

Figure S26: Forest plot of association between *rs6498486* polymorphism and the risk of head and neck cancer in allelic model

Study or Subgroup	Case Events	Case Total	Control Events	Control Total	Weight	Odds Ratio M-H, Fixed, 95% CI
Li,2014	29	129	22	132	46.8%	1.45 [0.78, 2.69]
Lu,2014	24	107	20	110	42.5%	1.30 [0.67, 2.53]
Xue, 2013	5	90	4	87	10.7%	1.22 [0.32, 4.70]
**Total (95% CI)**		**326**		**329**	**100.0%**	**1.36 [0.89, 2.09]**
Total events	58		46			
Heterogeneity: Chi² = 0.08, df = 2 (P = 0.96); I² = 0%						
Test for overall effect: Z = 1.41 (P = 0.16)						

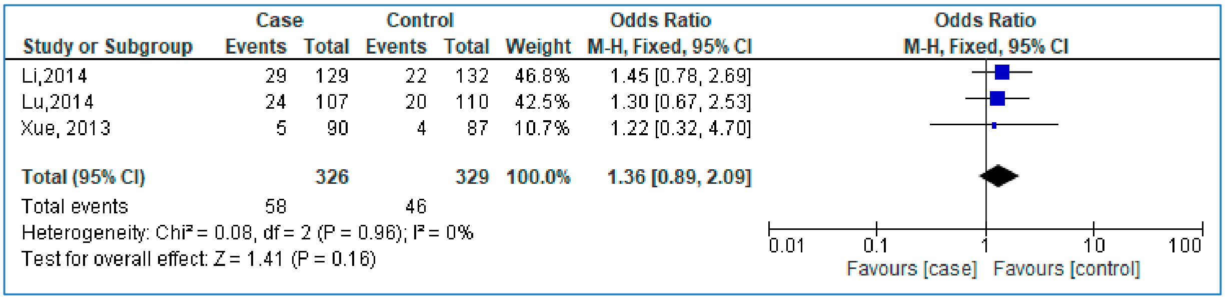

Figure S27: Forest plot of association between *rs6498486* polymorphism and the risk of head and neck cancer in homozygous model

Study or Subgroup	Case Events	Case Total	Control Events	Control Total	Weight	Odds Ratio M-H, Fixed, 95% CI
Li,2014	81	181	78	188	39.4%	1.14 [0.76, 1.73]
Lu,2014	69	152	66	156	33.2%	1.13 [0.72, 1.78]
Xue, 2013	52	137	46	129	27.4%	1.10 [0.67, 1.82]
**Total (95% CI)**		**470**		**473**	**100.0%**	**1.13 [0.87, 1.46]**
Total events	202		190			
Heterogeneity: Chi² = 0.01, df = 2 (P = 0.99); I² = 0%						
Test for overall effect: Z = 0.92 (P = 0.36)						

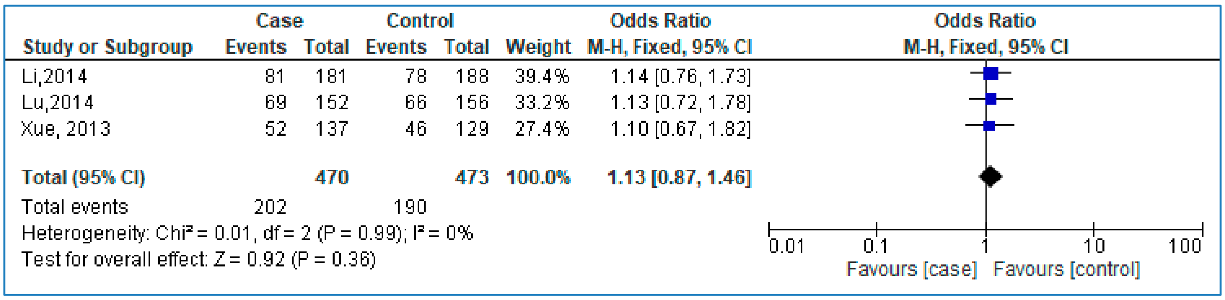

Figure S28: Forest plot of association between *rs6498486* polymorphism and the risk of head and neck cancer in heterozygous model

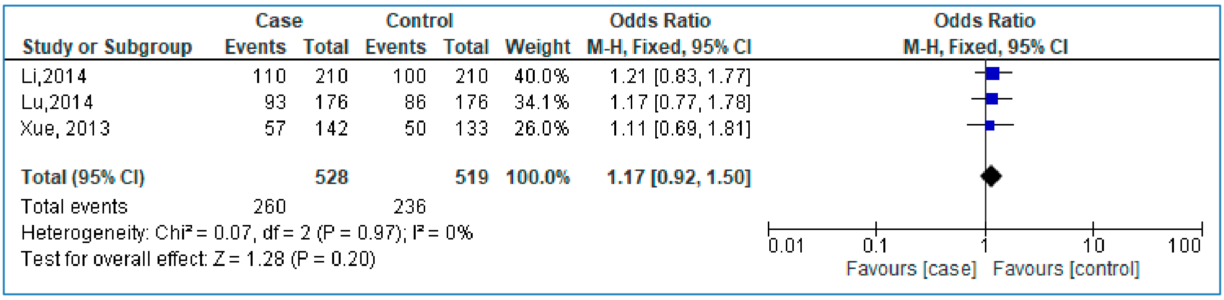

Figure S29: Forest plot of association between *rs6498486* polymorphism and the risk of head and neck cancer in dominant model

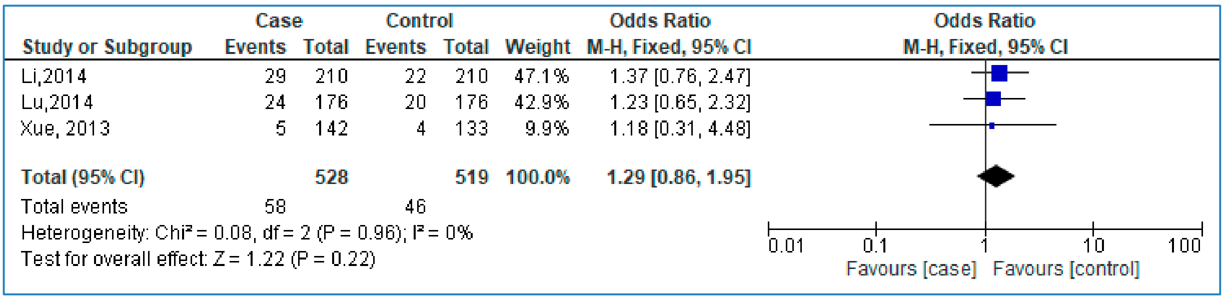

Figure S30: Forest plot of association between *rs6498486* polymorphism and the risk of head and neck cancer in recessive model

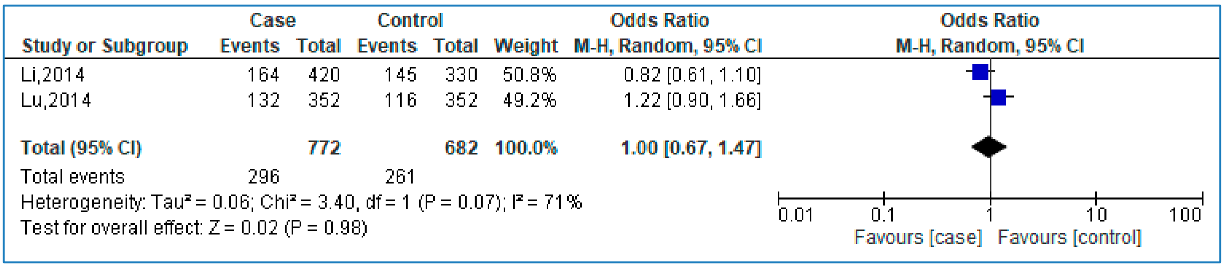

Figure S31: Forest plot of association between *rs2276465* polymorphism and the risk of head and neck cancer in allelic model

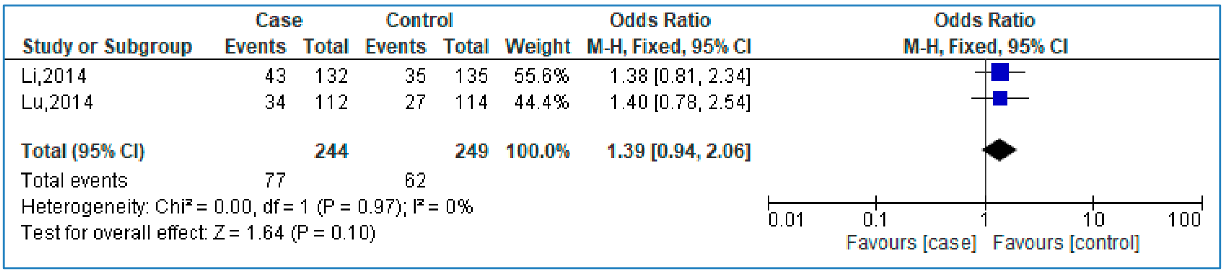

Figure S32: Forest plot of association between *rs2276465* polymorphism and the risk of head and neck cancer in homozygous model

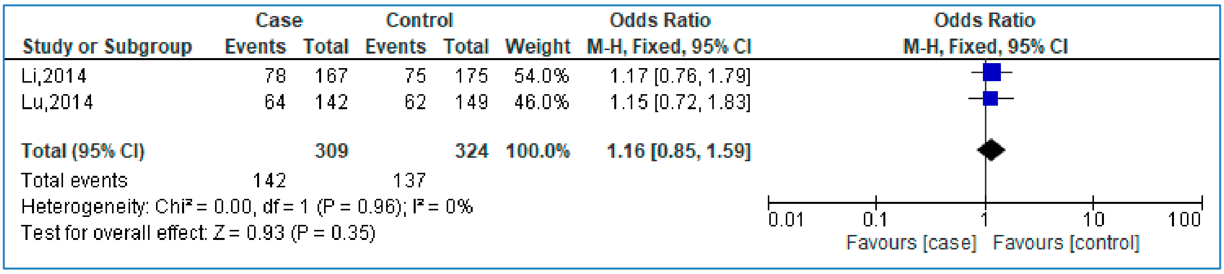

Figure S33: Forest plot of association between *rs2276465* polymorphism and the risk of head and neck cancer in heterozygous model

Study or Subgroup	Case Events	Case Total	Control Events	Control Total	Weight	Odds Ratio M-H, Random, 95% CI
Li,2014	121	363	110	210	50.7%	0.45 [0.32, 0.64]
Lu,2014	98	176	89	176	49.3%	1.23 [0.81, 1.87]
**Total (95% CI)**		**539**		**386**	**100.0%**	**0.74 [0.28, 1.96]**
Total events	219		199			

Heterogeneity: Tau² = 0.46; Chi² = 12.79, df = 1 (P = 0.0003); I² = 92%
Test for overall effect: Z = 0.60 (P = 0.55)

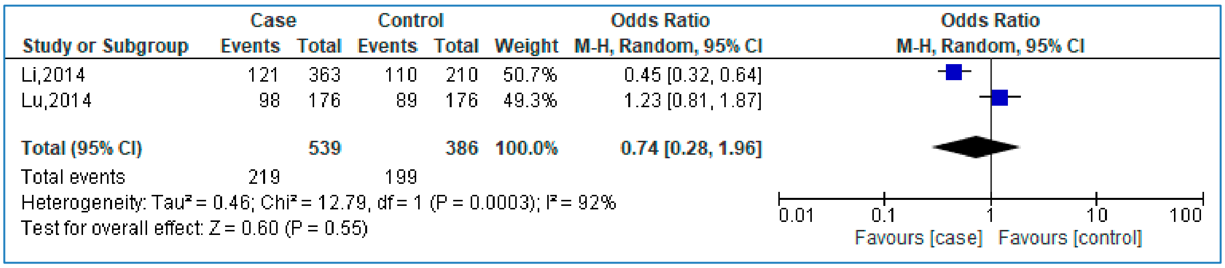

Figure S34: Forest plot of association between *rs2276465* polymorphism and the risk of head and neck cancer in dominant model

Study or Subgroup	Case Events	Case Total	Control Events	Control Total	Weight	Odds Ratio M-H, Random, 95% CI
Li,2014	43	363	35	210	52.1%	0.67 [0.41, 1.09]
Lu,2014	34	176	27	176	47.9%	1.32 [0.76, 2.30]
**Total (95% CI)**		**539**		**386**	**100.0%**	**0.93 [0.48, 1.80]**
Total events	77		62			

Heterogeneity: Tau² = 0.16; Chi² = 3.25, df = 1 (P = 0.07); I² = 69%
Test for overall effect: Z = 0.22 (P = 0.83)

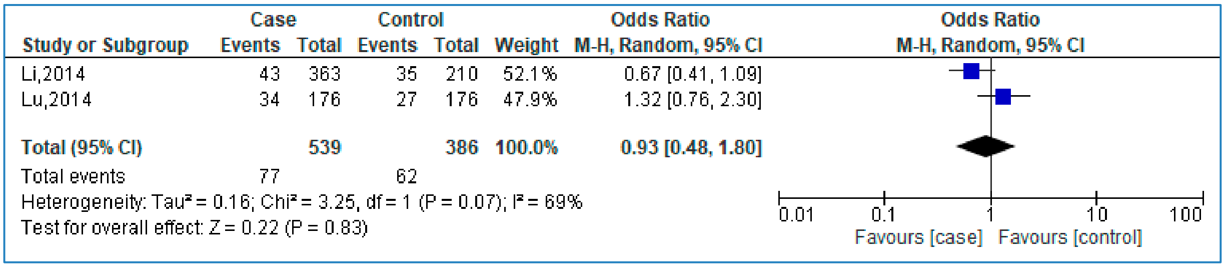

Figure S35: Forest plot of association between *rs2276465* polymorphism and the risk of head and neck cancer in recessive model

Study or Subgroup	Case Events	Case Total	Control Events	Control Total	Weight	Odds Ratio M-H, Fixed, 95% CI
Li,2014	106	420	99	420	14.3%	1.09 [0.80, 1.50]
Yu,2012	593	2080	622	2092	85.7%	0.94 [0.82, 1.08]
**Total (95% CI)**		**2500**		**2512**	**100.0%**	**0.96 [0.85, 1.09]**
Total events	699		721			

Heterogeneity: Chi² = 0.73, df = 1 (P = 0.39); I² = 0%
Test for overall effect: Z = 0.58 (P = 0.56)

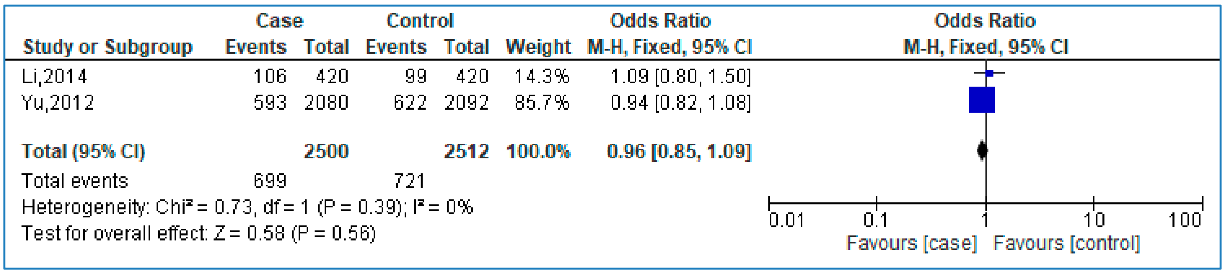

Figure S36: Forest plot of association between *rs2276466* polymorphism and the risk of head and neck cancer in allelic model

Study or Subgroup	Case Events	Case Total	Control Events	Control Total	Weight	Odds Ratio M-H, Random, 95% CI
Li,2014	19	142	16	143	49.3%	1.23 [0.60, 2.49]
Yu,2012	75	597	412	634	50.7%	0.08 [0.06, 0.10]
**Total (95% CI)**		**739**		**777**	**100.0%**	**0.30 [0.02, 4.54]**
Total events	94		428			

Heterogeneity: Tau² = 3.75; Chi² = 49.89, df = 1 (P < 0.00001); I² = 98%
Test for overall effect: Z = 0.87 (P = 0.39)

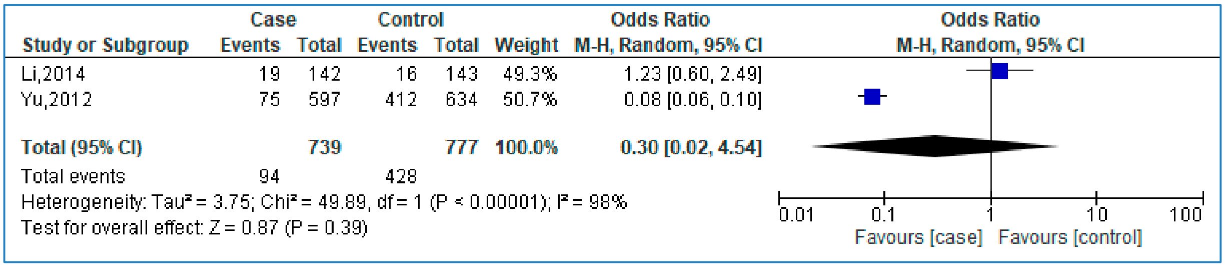

Figure S37: Forest plot of association between *rs2276466* polymorphism and the risk of head and neck cancer in homozygous model

Study or Subgroup	Case Events	Case Total	Control Events	Control Total	Weight	Odds Ratio M-H, Fixed, 95% CI
Li,2014	68	191	67	194	15.9%	1.05 [0.69, 1.59]
Yu,2012	443	965	412	941	84.1%	1.09 [0.91, 1.31]
**Total (95% CI)**		**1156**		**1135**	**100.0%**	**1.08 [0.92, 1.28]**
Total events	511		479			

Heterogeneity: Chi² = 0.03, df = 1 (P = 0.87); I² = 0%
Test for overall effect: Z = 0.94 (P = 0.35)

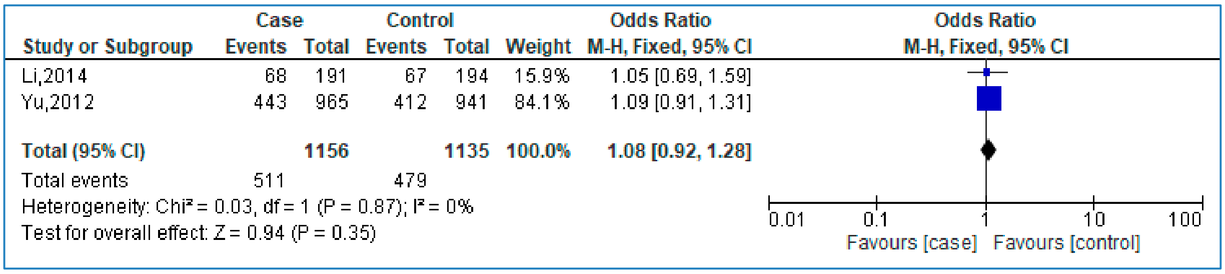

Figure S38: Forest plot of association between *rs2276466* polymorphism and the risk of head and neck cancer in heterozygous model

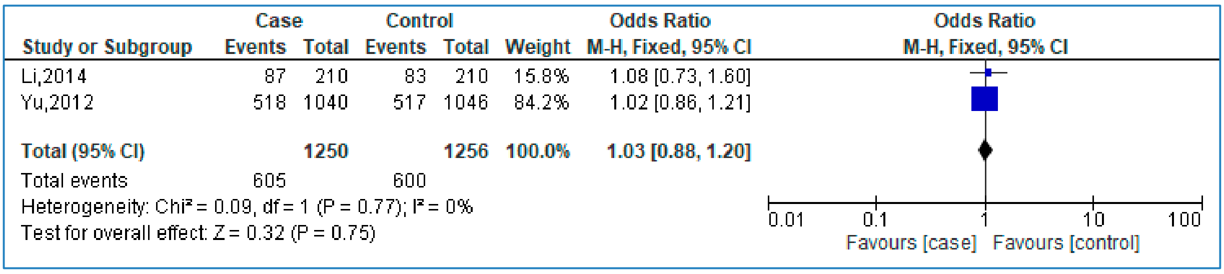

Figure S39: Forest plot of association between *rs2276466* polymorphism and the risk of head and neck cancer in dominant model

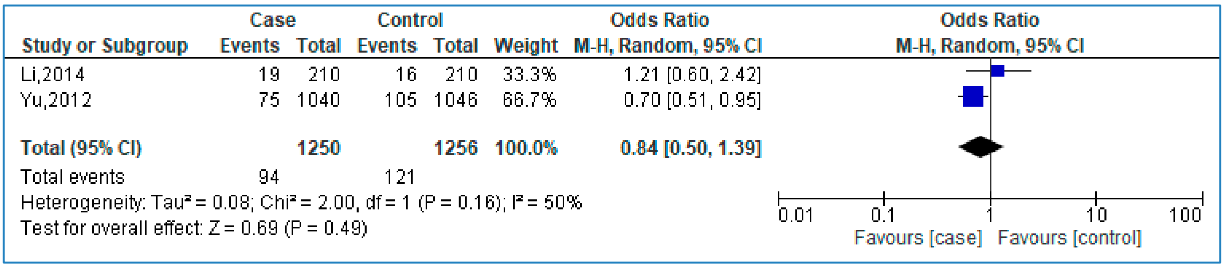

Figure S40: Forest plot of association between *rs2276466* polymorphism and the risk of head and neck cancer in recessive model

Study or Subgroup	Case Events	Case Total	Control Events	Control Total	Weight	Odds Ratio M-H, Fixed, 95% CI
Li,2014	156	420	146	420	54.0%	1.11 [0.84, 1.47]
Lu,2014	128	352	123	352	46.0%	1.06 [0.78, 1.45]
**Total (95% CI)**		**772**		**772**	**100.0%**	**1.09 [0.88, 1.34]**
Total events	284		269			

Heterogeneity: Chi² = 0.04, df = 1 (P = 0.85); I² = 0%
Test for overall effect: Z = 0.80 (P = 0.43)

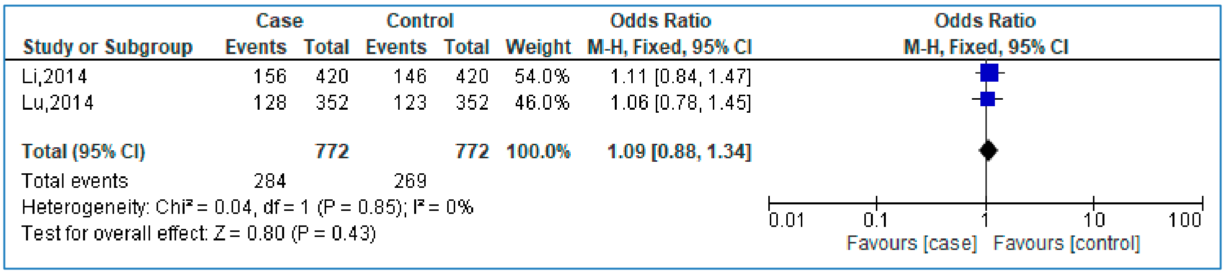

Figure S41: Forest plot of association between *rs4150441* polymorphism and the risk of head and neck cancer in allelic model

Study or Subgroup	Case Events	Case Total	Control Events	Control Total	Weight	Odds Ratio M-H, Fixed, 95% CI
Li,2014	38	130	35	134	56.4%	1.17 [0.68, 2.00]
Lu,2014	30	98	29	111	43.6%	1.25 [0.68, 2.28]
**Total (95% CI)**		**228**		**245**	**100.0%**	**1.20 [0.80, 1.80]**
Total events	68		64			

Heterogeneity: Chi² = 0.03, df = 1 (P = 0.87); I² = 0%
Test for overall effect: Z = 0.90 (P = 0.37)

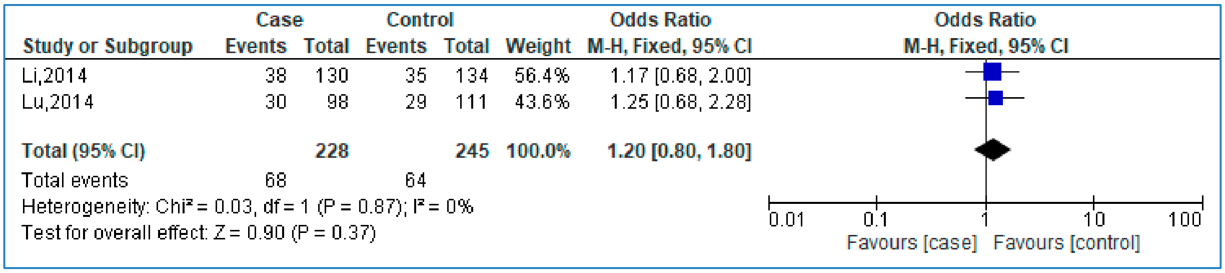

Figure S42: Forest plot of association between *rs4150441* polymorphism and the risk of head and neck cancer in homozygous model

Study or Subgroup	Case Events	Case Total	Control Events	Control Total	Weight	Odds Ratio M-H, Fixed, 95% CI
Li,2014	80	172	76	175	53.8%	1.13 [0.74, 1.73]
Lu,2014	68	146	65	147	46.2%	1.10 [0.69, 1.74]
**Total (95% CI)**		**318**		**322**	**100.0%**	**1.12 [0.82, 1.53]**
Total events	148		141			

Heterogeneity: Chi² = 0.01, df = 1 (P = 0.93); I² = 0%
Test for overall effect: Z = 0.70 (P = 0.48)

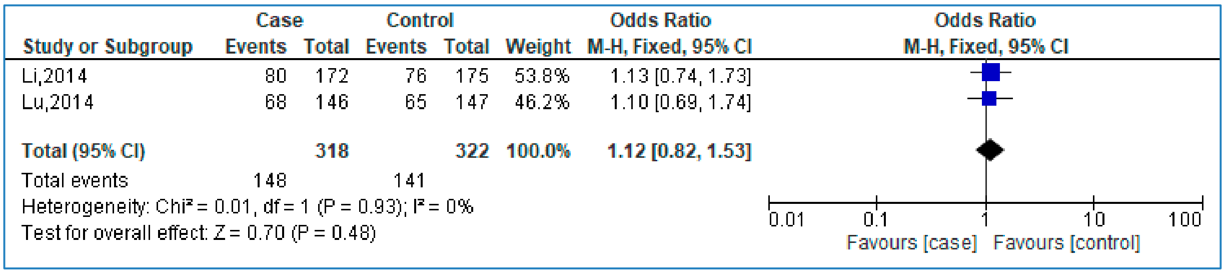

Figure S43: Forest plot of association between *rs4150441* polymorphism and the risk of head and neck cancer in heterozygous model

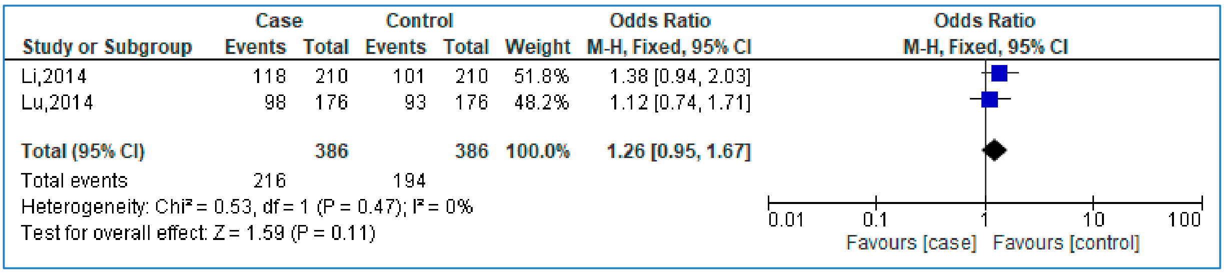

Figure S44: Forest plot of association between *rs4150441* polymorphism and the risk of head and neck cancer in dominant model

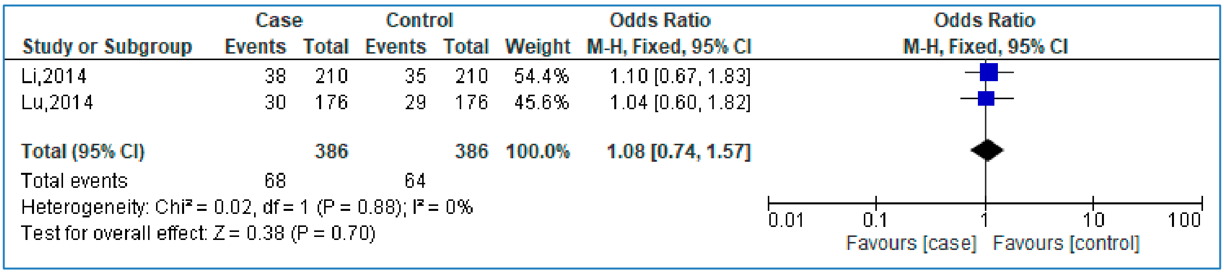

Figure S45: Forest plot of association between *rs4150441* polymorphism and the risk of head and neck cancer in recessive model

Study or Subgroup	Case Events	Case Total	Control Events	Control Total	Weight	Odds Ratio M-H, Fixed, 95% CI
Abbasi,2009	169	492	425	1288	11.3%	1.06 [0.85, 1.32]
An,2007	580	1658	602	1708	28.2%	0.99 [0.86, 1.14]
Bau,2007	160	308	111	210	4.6%	0.96 [0.68, 1.37]
Hall, 2007	397	1194	571	834	32.8%	0.23 [0.19, 0.28]
Jelonek,2010	41	132	95	226	3.5%	0.62 [0.39, 0.98]
Li,2014	171	420	157	420	6.8%	1.15 [0.87, 1.52]
Lu,2014	141	352	134	352	5.9%	1.09 [0.80, 1.47]
Sugimura, 2006	111	244	253	482	6.8%	0.76 [0.55, 1.03]
**Total (95% CI)**		**4800**		**5520**	**100.0%**	**0.73 [0.68, 0.80]**
Total events	1770		2348			

Heterogeneity: Chi² = 192.29, df = 7 (P < 0.00001); I² = 96%
Test for overall effect: Z = 7.46 (P < 0.00001)

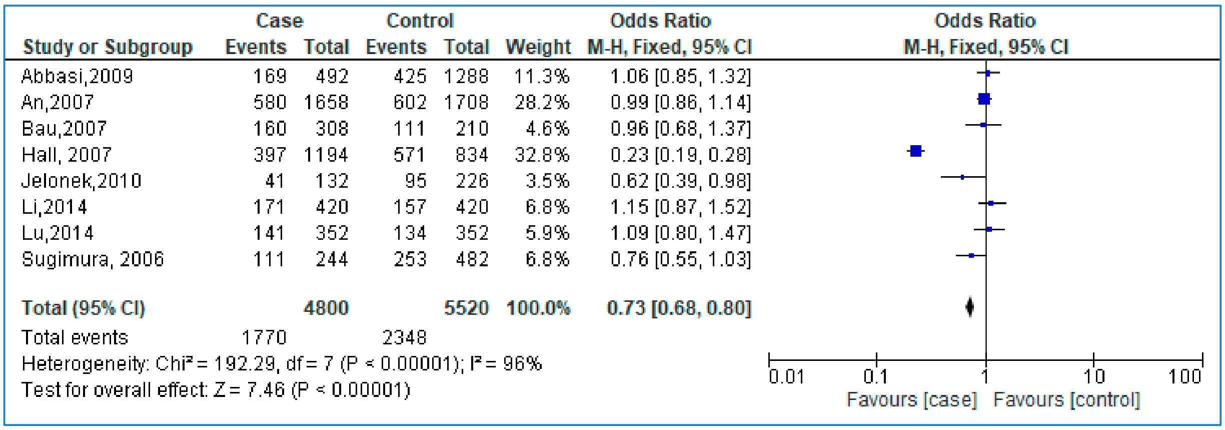

Figure S46: Forest plot of association between *rs1800975* polymorphism and the risk of head and neck cancer in allelic model

Study or Subgroup	Case Events	Case Total	Control Events	Control Total	Weight	Odds Ratio M-H, Fixed, 95% CI
Abbasi,2009	30	137	72	363	10.1%	1.13 [0.70, 1.83]
An,2007	110	469	128	508	30.9%	0.91 [0.68, 1.22]
Bau,2007	38	70	29	52	5.0%	0.94 [0.46, 1.94]
Hall, 2007	75	350	98	395	23.8%	0.83 [0.59, 1.16]
Jelonek,2010	4	33	15	48	3.5%	0.30 [0.09, 1.02]
Li,2014	52	143	47	147	9.7%	1.22 [0.75, 1.98]
Lu,2014	43	121	40	122	8.4%	1.13 [0.66, 1.92]
Sugimura, 2006	23	57	74	136	8.6%	0.57 [0.30, 1.06]
**Total (95% CI)**		**1380**		**1771**	**100.0%**	**0.91 [0.77, 1.07]**
Total events	375		503			

Heterogeneity: Chi² = 8.46, df = 7 (P = 0.29); I² = 17%
Test for overall effect: Z = 1.11 (P = 0.27)

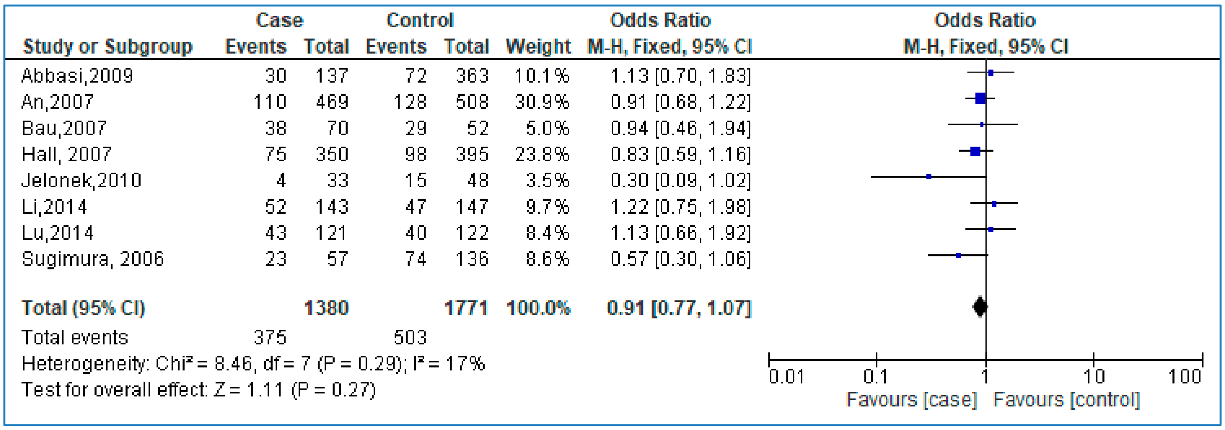

Figure S47: Forest plot of association between *rs1800975* polymorphism and the risk of head and neck cancer in homozygous model

Study or Subgroup	Case Events	Case Total	Control Events	Control Total	Weight	Odds Ratio M-H, Fixed, 95% CI
Abbasi,2009	109	216	281	572	13.9%	1.05 [0.77, 1.44]
An,2007	360	719	346	726	31.4%	1.10 [0.90, 1.35]
Bau,2007	84	116	53	76	3.2%	1.14 [0.60, 2.15]
Hall, 2007	247	522	375	672	31.5%	0.71 [0.57, 0.89]
Jelonek,2010	33	62	65	98	4.3%	0.58 [0.30, 1.11]
Li,2014	67	158	63	163	6.5%	1.17 [0.75, 1.83]
Lu,2014	55	133	54	136	5.7%	1.07 [0.66, 1.74]
Sugimura, 2006	65	88	105	167	3.5%	1.67 [0.94, 2.95]
**Total (95% CI)**		**2014**		**2610**	**100.0%**	**0.97 [0.86, 1.10]**
Total events	1020		1342			

Heterogeneity: $Chi^2 = 15.73$, df = 7 (P = 0.03); $I^2 = 55\%$
Test for overall effect: Z = 0.45 (P = 0.65)

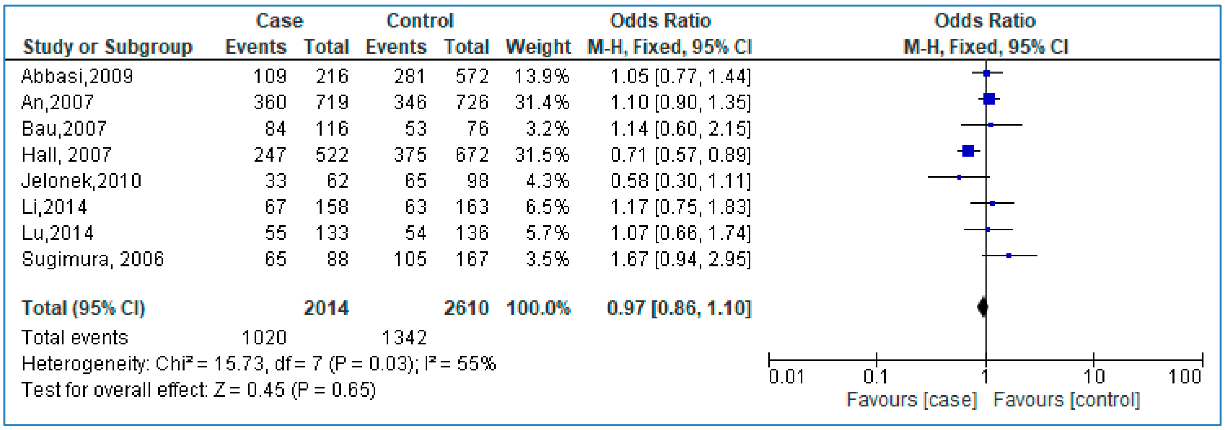

Figure S48: Forest plot of association between *rs1800975* polymorphism and the risk of head and neck cancer in heterozygous model

Study or Subgroup	Case Events	Case Total	Control Events	Control Total	Weight	Odds Ratio M-H, Fixed, 95% CI
Abbasi,2009	139	246	353	644	13.1%	1.07 [0.80, 1.44]
An,2007	470	829	474	854	31.3%	1.05 [0.87, 1.27]
Bau,2007	122	154	82	105	3.1%	1.07 [0.58, 1.96]
Hall, 2007	322	597	473	770	29.4%	0.74 [0.59, 0.91]
Jelonek,2010	37	66	80	113	4.0%	0.53 [0.28, 0.99]
Li,2014	119	210	110	210	7.4%	1.19 [0.81, 1.75]
Lu,2014	98	176	94	176	6.4%	1.10 [0.72, 1.67]
Sugimura, 2006	88	122	179	241	5.2%	0.90 [0.55, 1.46]
**Total (95% CI)**		**2400**		**3113**	**100.0%**	**0.94 [0.85, 1.06]**
Total events	1395		1845			

Heterogeneity: $Chi^2 = 12.32$, df = 7 (P = 0.09); $I^2 = 43\%$
Test for overall effect: Z = 1.01 (P = 0.31)

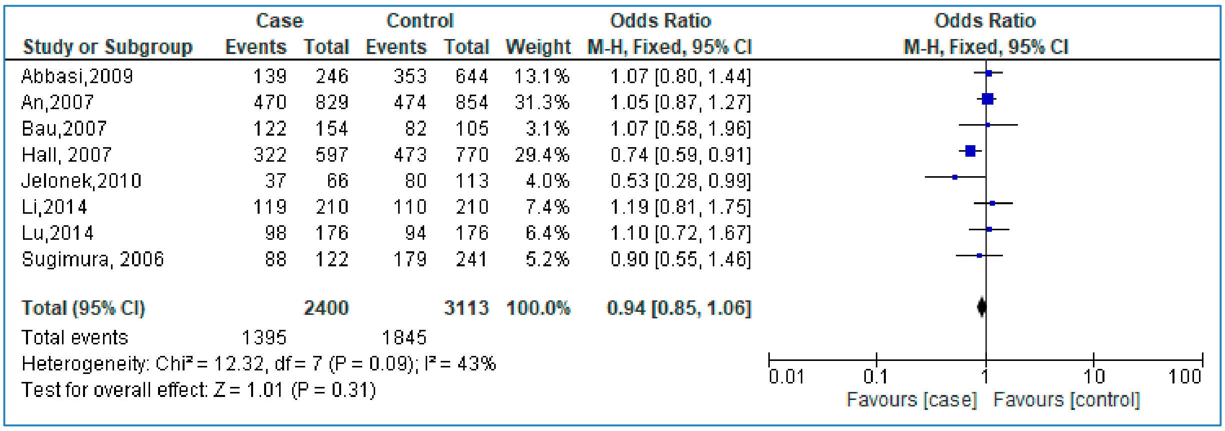

Figure S49: Forest plot of association between *rs1800975* polymorphism and the risk of head and neck cancer in dominant model

Study or Subgroup	Case Events	Case Total	Control Events	Control Total	Weight	Odds Ratio M-H, Fixed, 95% CI
Abbasi,2009	30	246	72	644	6.1%	1.10 [0.70, 1.74]
An,2007	110	829	128	854	19.1%	0.87 [0.66, 1.14]
Bau,2007	38	154	29	105	4.5%	0.86 [0.49, 1.51]
Hall, 2007	75	597	375	770	50.0%	0.15 [0.11, 0.20]
Jelonek,2010	4	66	15	113	1.8%	0.42 [0.13, 1.33]
Li,2014	52	210	47	210	6.2%	1.14 [0.73, 1.79]
Lu,2014	43	176	40	176	5.3%	1.10 [0.67, 1.80]
Sugimura, 2006	23	122	74	241	7.0%	0.52 [0.31, 0.89]
**Total (95% CI)**		**2400**		**3113**	**100.0%**	**0.52 [0.45, 0.60]**
Total events	375		780			

Heterogeneity: $Chi^2 = 122.09$, df = 7 (P < 0.00001); $I^2 = 94\%$
Test for overall effect: Z = 9.24 (P < 0.00001)

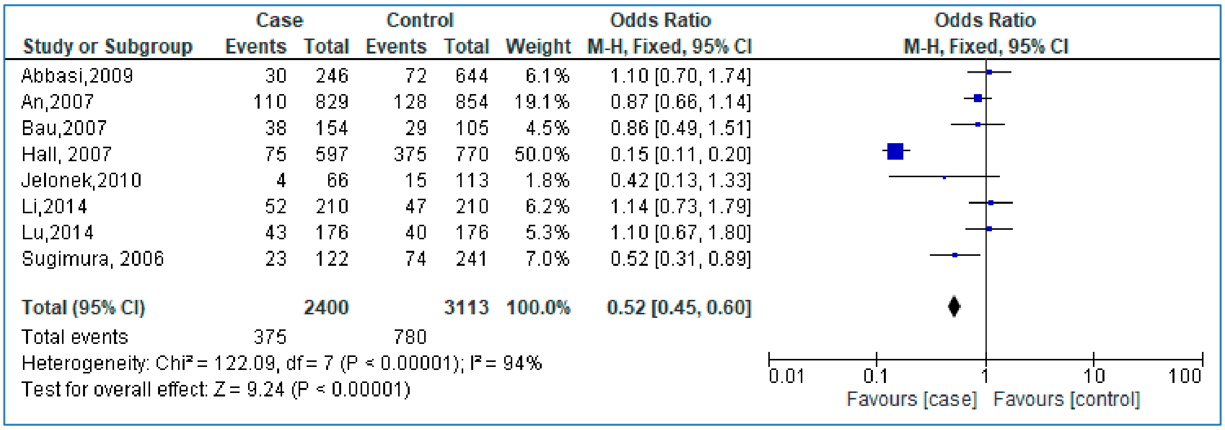

Figure S50: Forest plot of association between *rs1800975* polymorphism and the risk of head and neck cancer in recessive model
