# Supplementary material for: Relationship between XPA, XPB/ERCC3, XPF/ERCC4, and XPG/ERCC5 Polymorphisms and the Susceptibility to Head and Neck Carcinoma: A Systematic Review, Meta-Analysis, and Trial Sequential Analysis
Source: Medicina (Kaunas). 2024 Mar 14;60(3):478. doi: 10.3390/medicina60030478 (PMC10972270; doi:10.3390/medicina60030478)
Supplement: Supplementary file 1 [file medicina-60-00478-s001.zip › Supplementary File S2.pdf]

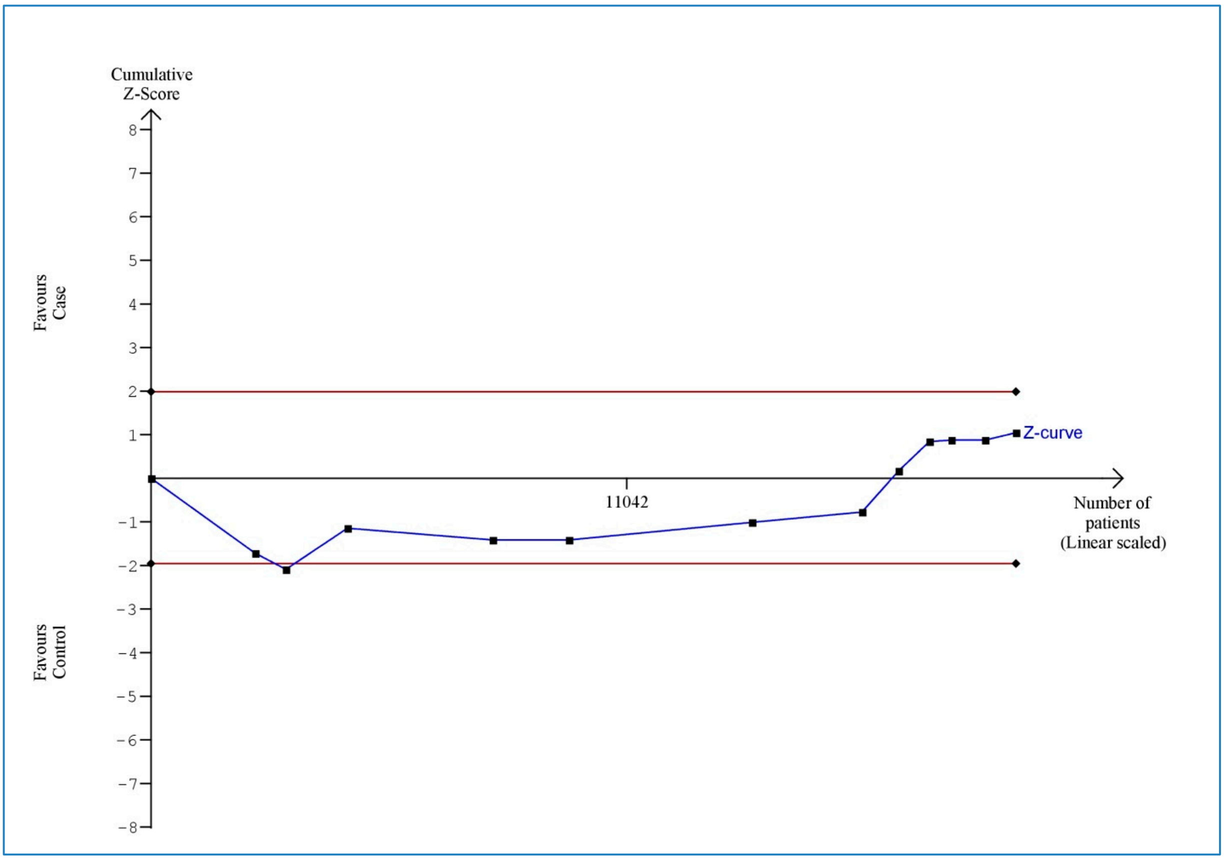

Figure S1: Trial sequential analysis of association between *rs17655* polymorphism and the risk of head and neck cancer in allelic model (D^2 = 61%).

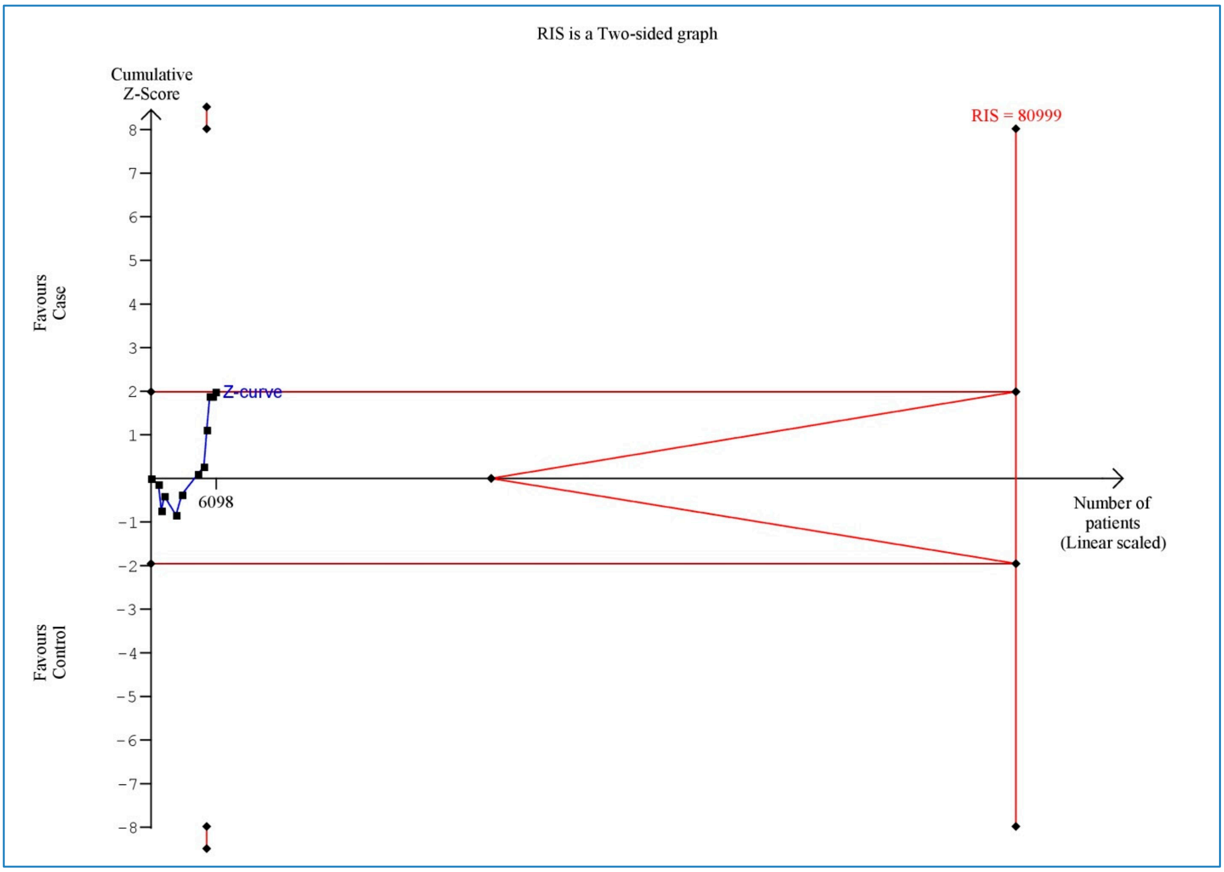

Figure S2: Trial sequential analysis of association between *rs17655* polymorphism and the risk of head and neck cancer in homozygous model ($D^2 = 39\%$).

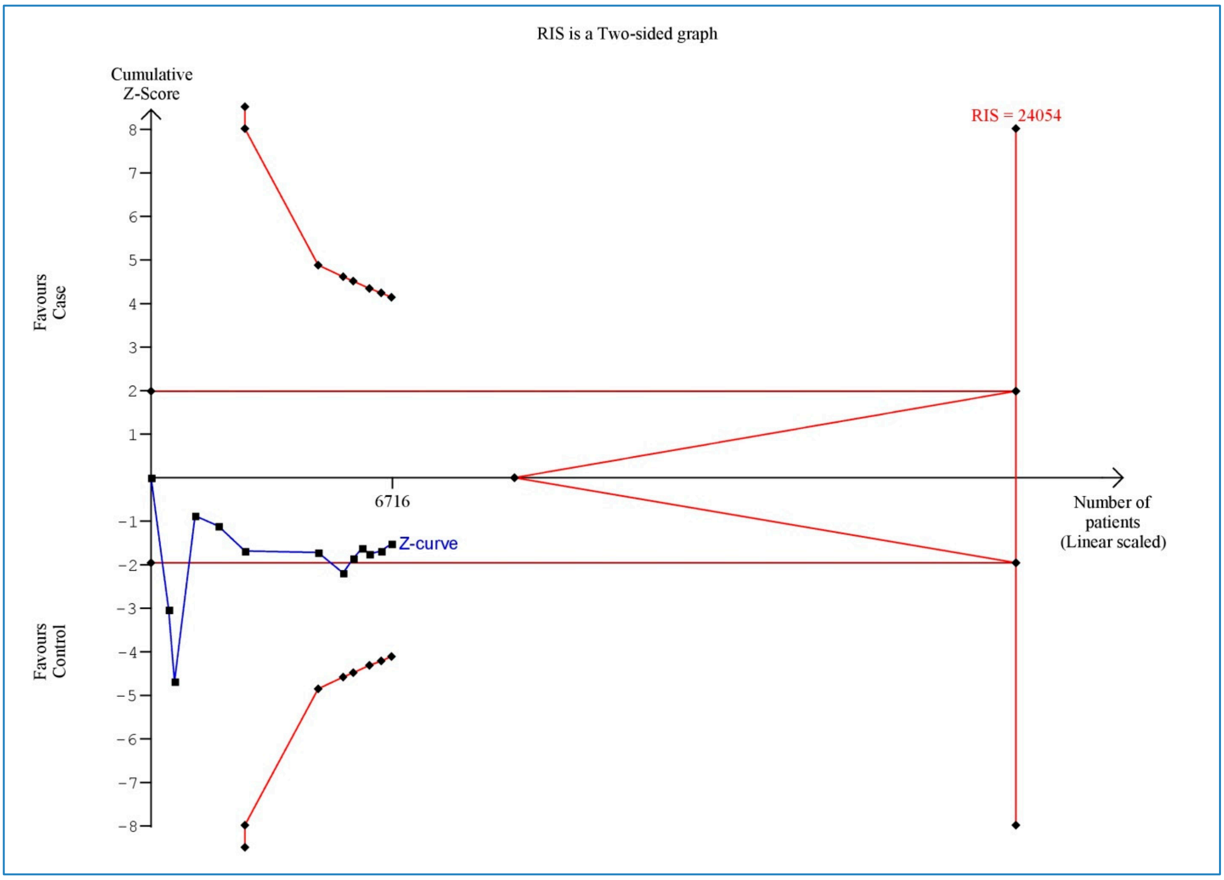

Figure S3: Trial sequential analysis of association between *rs17655* polymorphism and the risk of head and neck cancer in heterozygous model (D^2 = 87%).

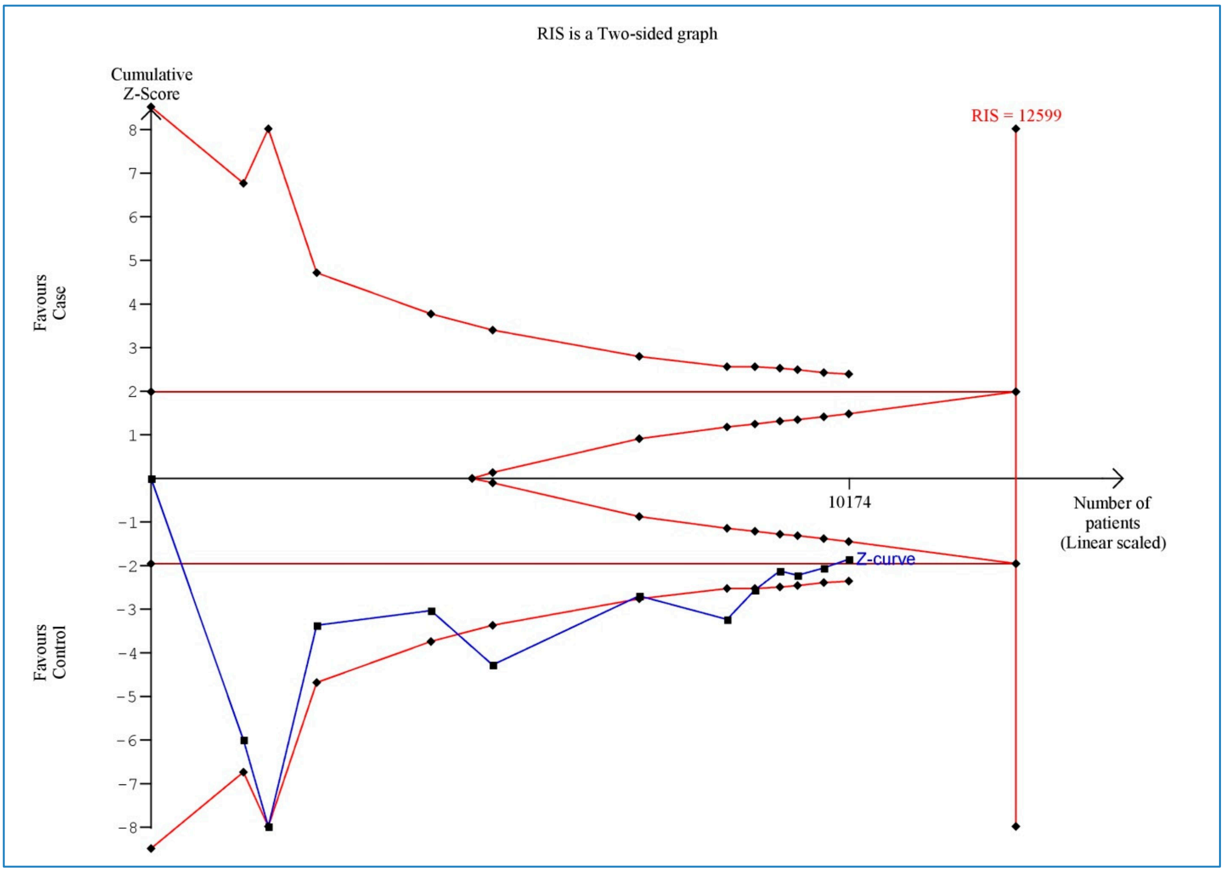

Figure S4: Trial sequential analysis of association between *rs17655* polymorphism and the risk of head and neck cancer in dominant model ($D^2 = 94\%$).

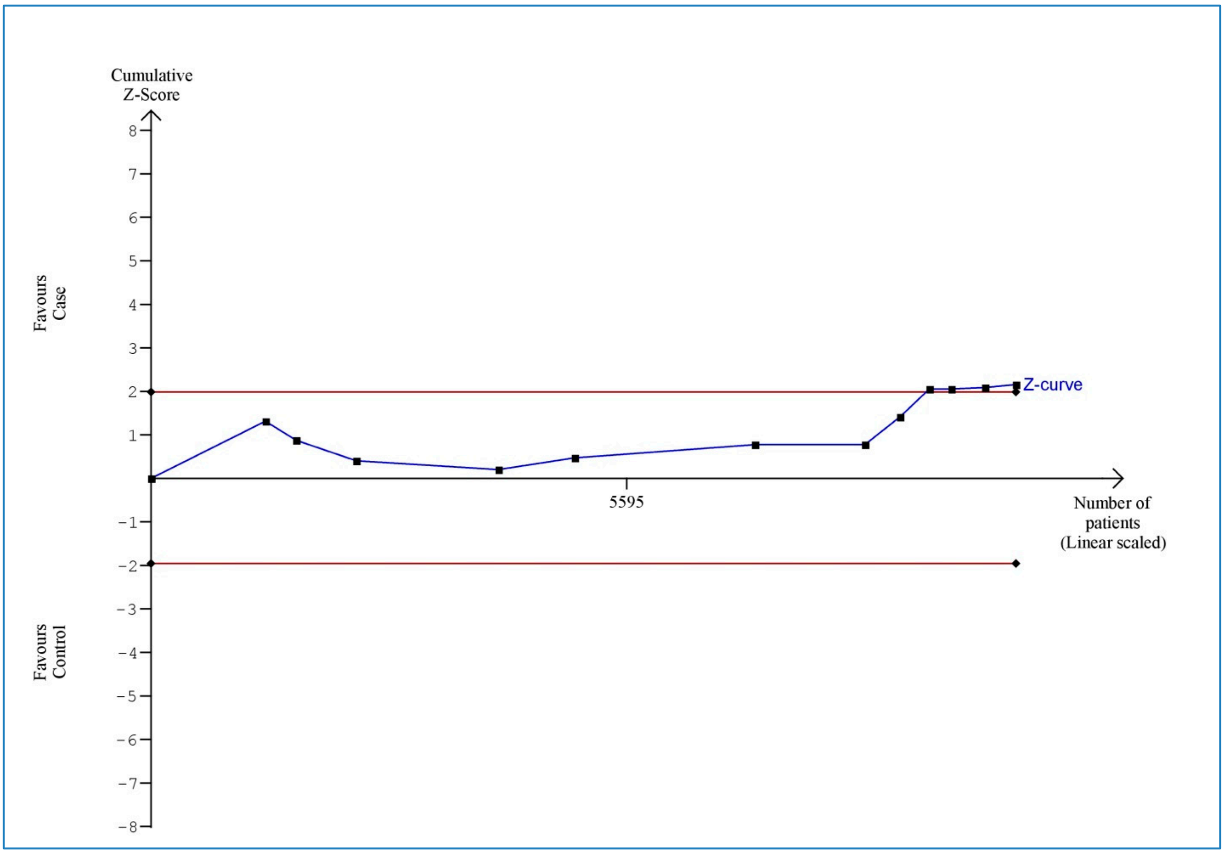

Figure S5: Trial sequential analysis of association between *rs17655* polymorphism and the risk of head and neck cancer in recessive model (D^2 = 95%).

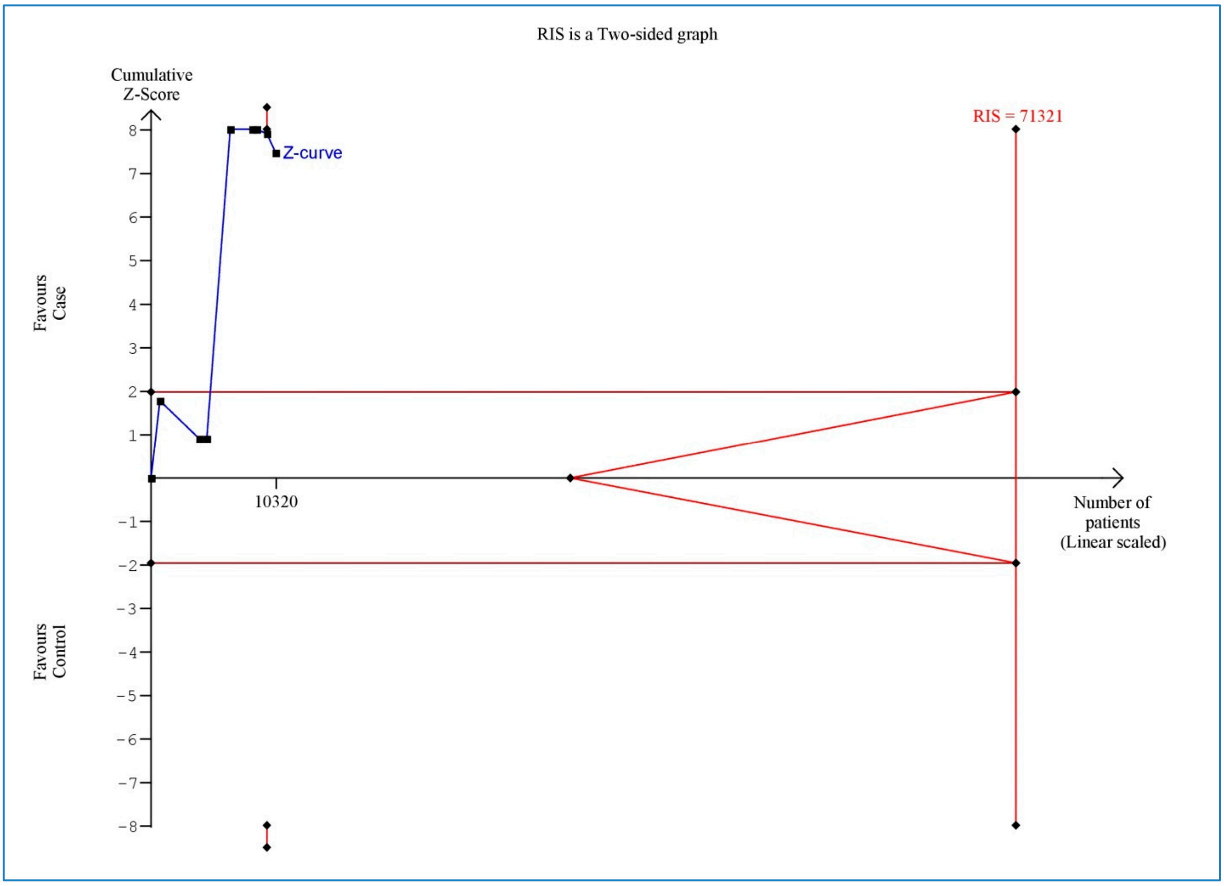

Figure S6: Trial sequential analysis of association between *rs1800975* polymorphism and the risk of head and neck cancer in allelic model (D^2 = 97%).

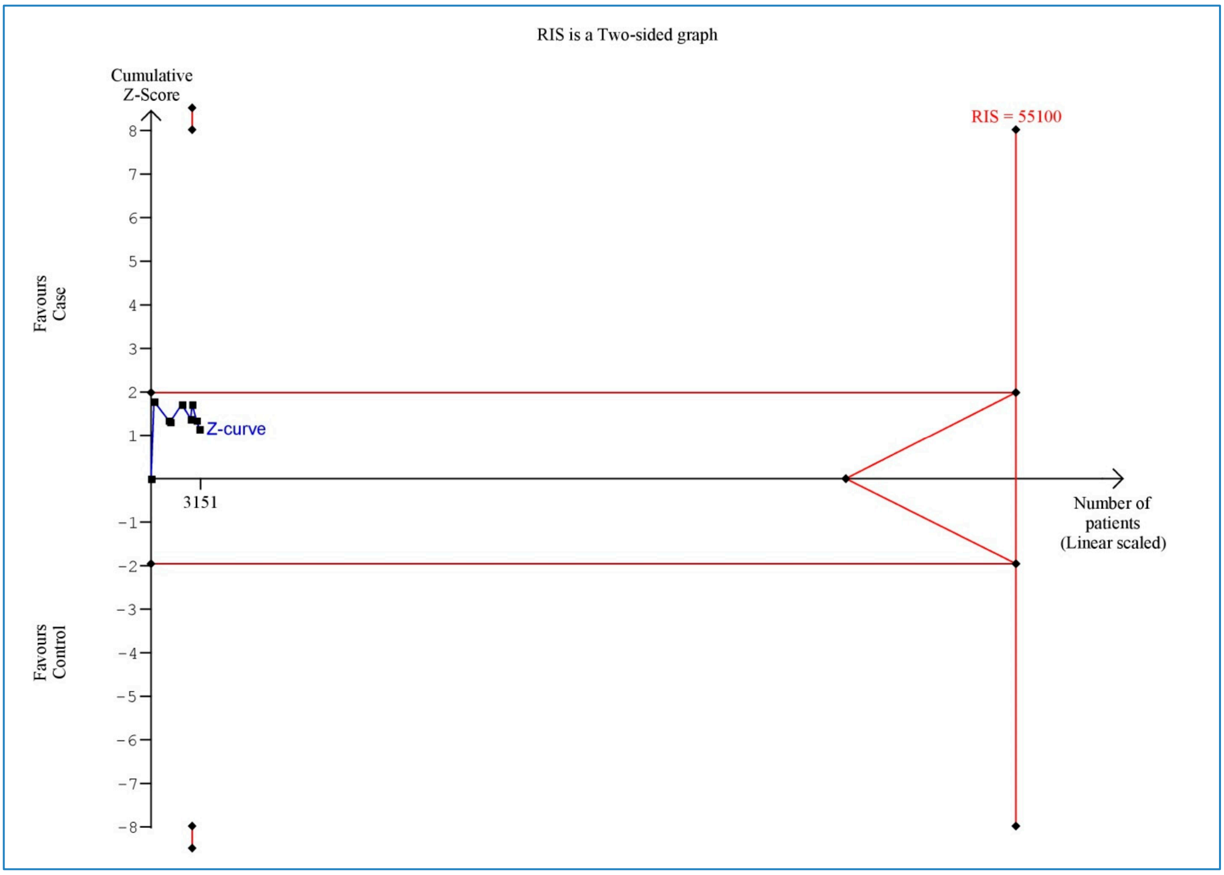

Figure S7: Trial sequential analysis of association between *rs1800975* polymorphism and the risk of head and neck cancer in homozygous model (D^2 = 24%).

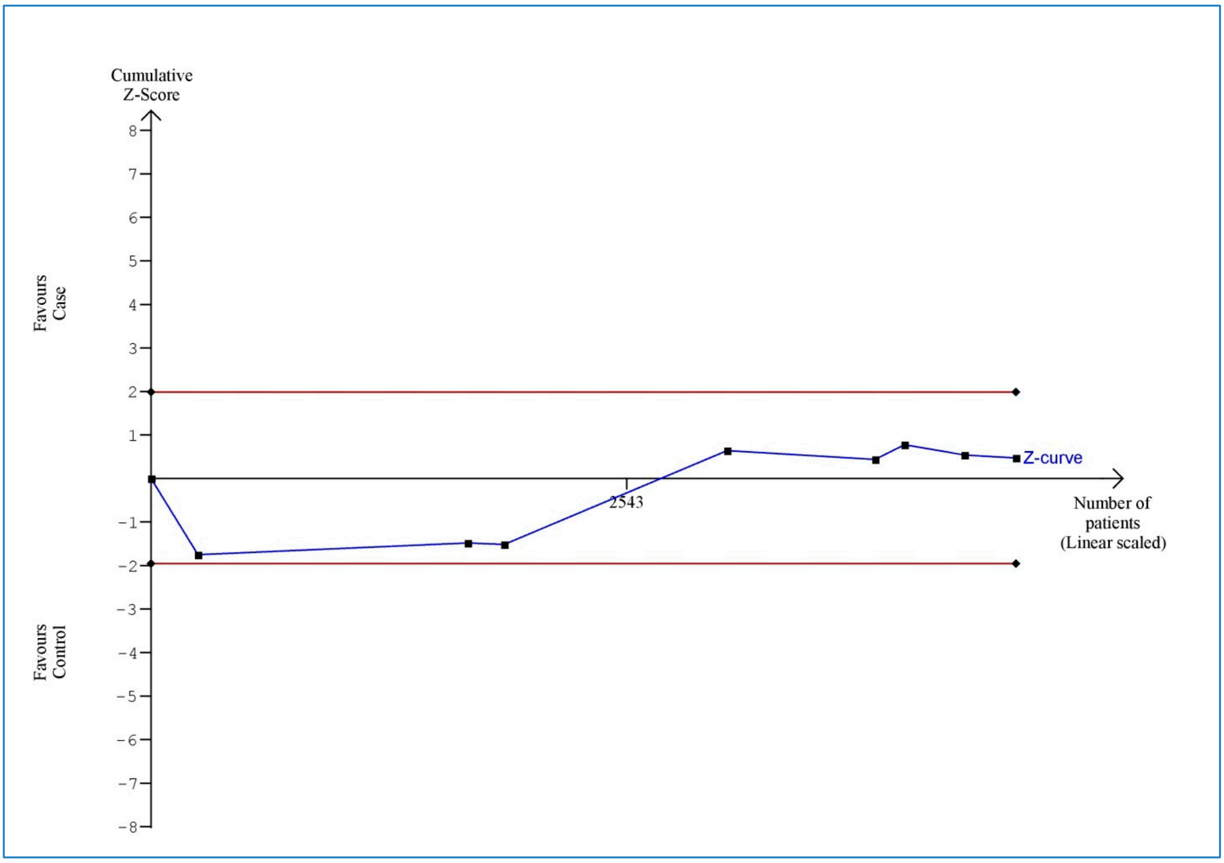

Figure S8: Trial sequential analysis of association between *rs1800975* polymorphism and the risk of head and neck cancer in heterozygous model (D^2 = 65%).

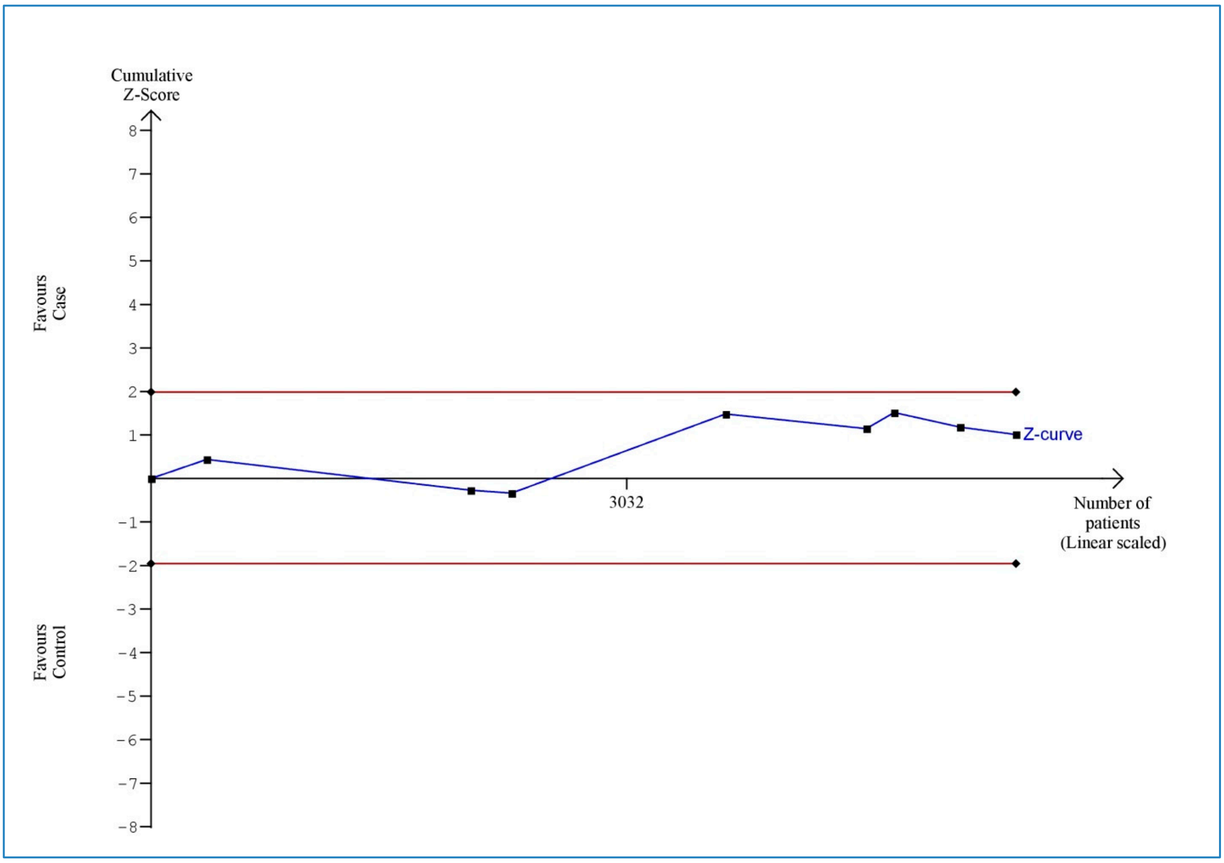

Figure S9: Trial sequential analysis of association between *rs1800975* polymorphism and the risk of head and neck cancer in dominant model (D^2 = 54%).

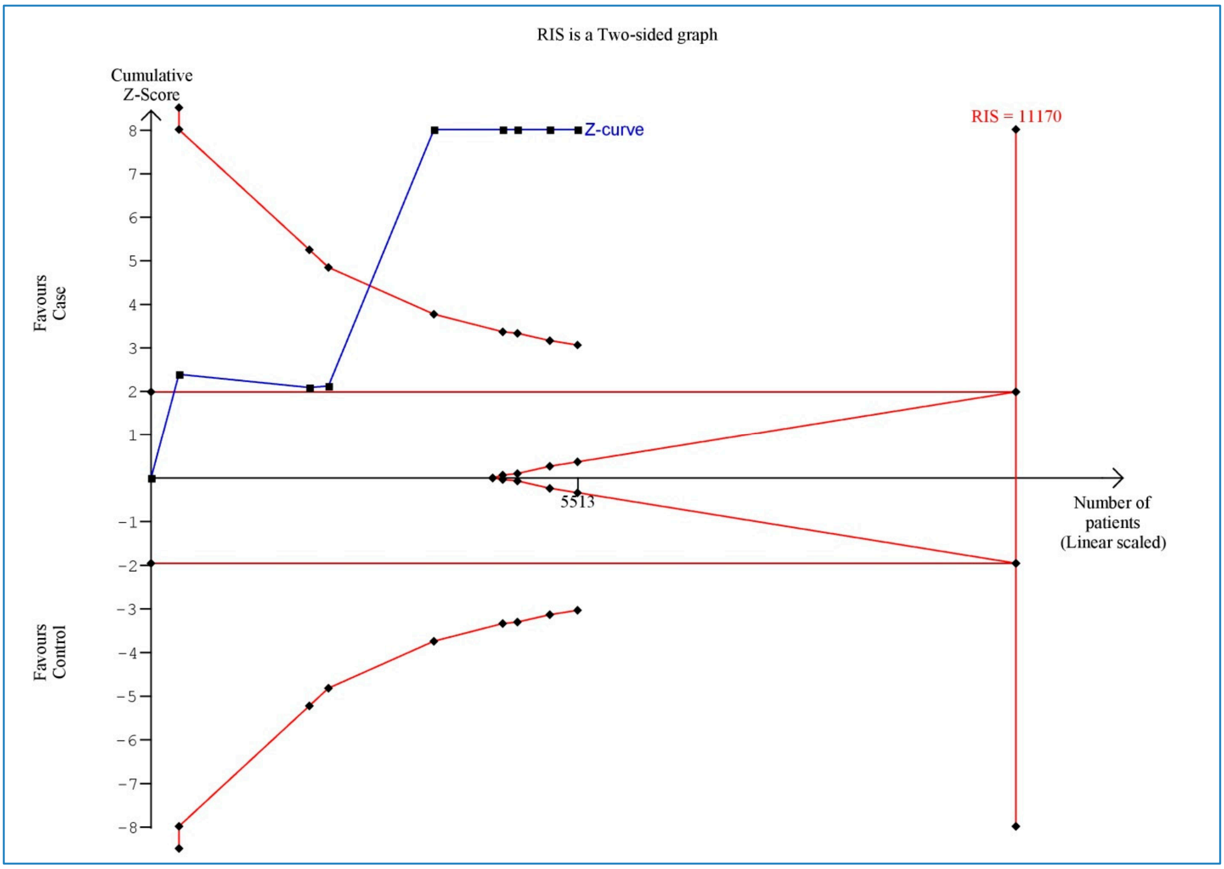

Figure S10: Trial sequential analysis of association between *rs1800975* polymorphism and the risk of head and neck cancer in recessive model (D^2 = 95%).
