# Supplementary material for: Relationship between XPA, XPB/ERCC3, XPF/ERCC4, and XPG/ERCC5 Polymorphisms and the Susceptibility to Head and Neck Carcinoma: A Systematic Review, Meta-Analysis, and Trial Sequential Analysis
Source: Medicina (Kaunas). 2024 Mar 14;60(3):478. doi: 10.3390/medicina60030478 (PMC10972270; doi:10.3390/medicina60030478)
Supplement: Supplementary file 1 [file medicina-60-00478-s001.zip › Supplementary File S3.pdf]

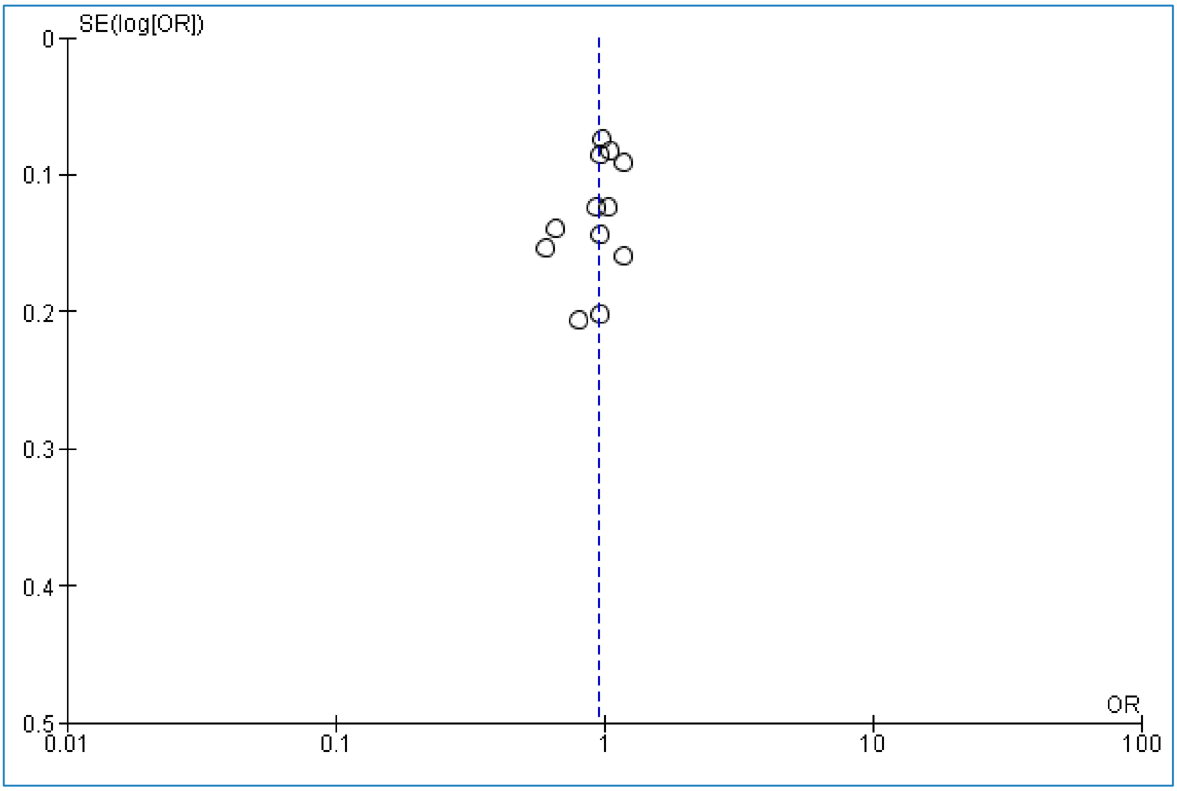


**Figure S1**: Funnel plot of association between *rs17655* polymorphism and the risk of head and neck cancer in allelic model

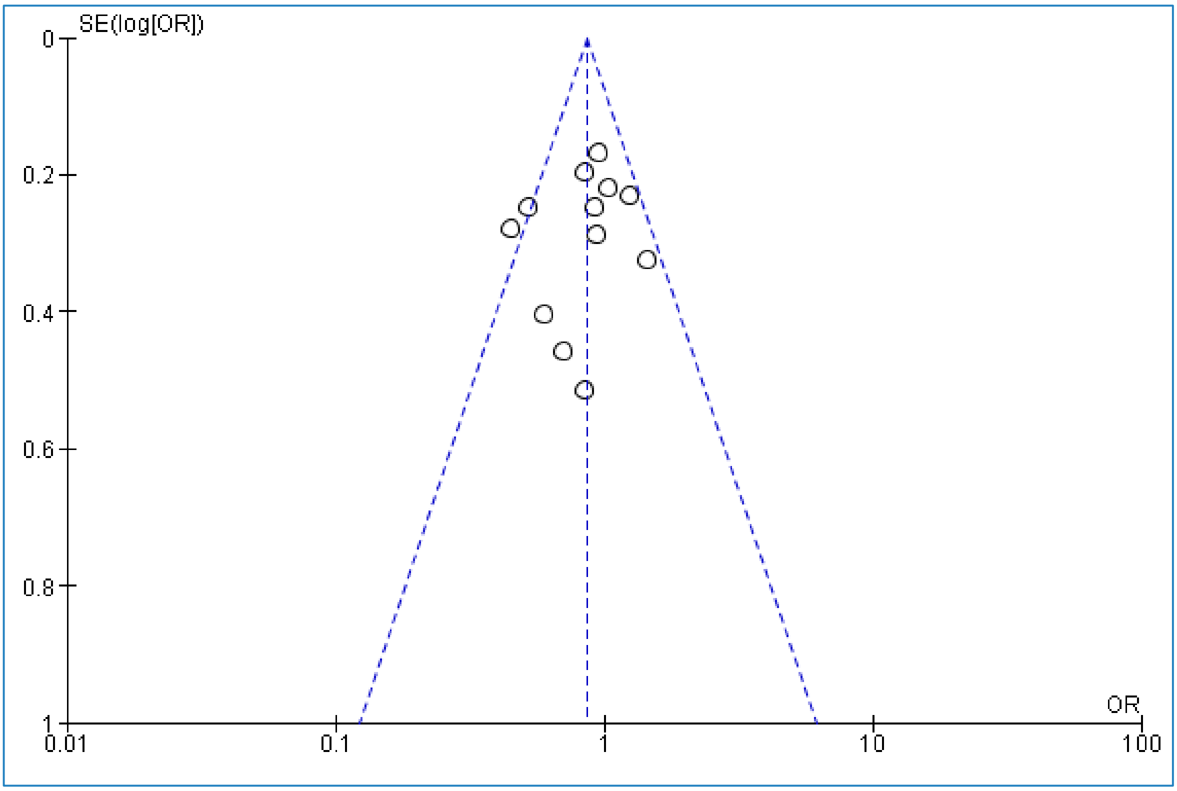


**Figure S2**: Funnel plot of association between *rs17655* polymorphism and the risk of head and neck cancer in homozygous model

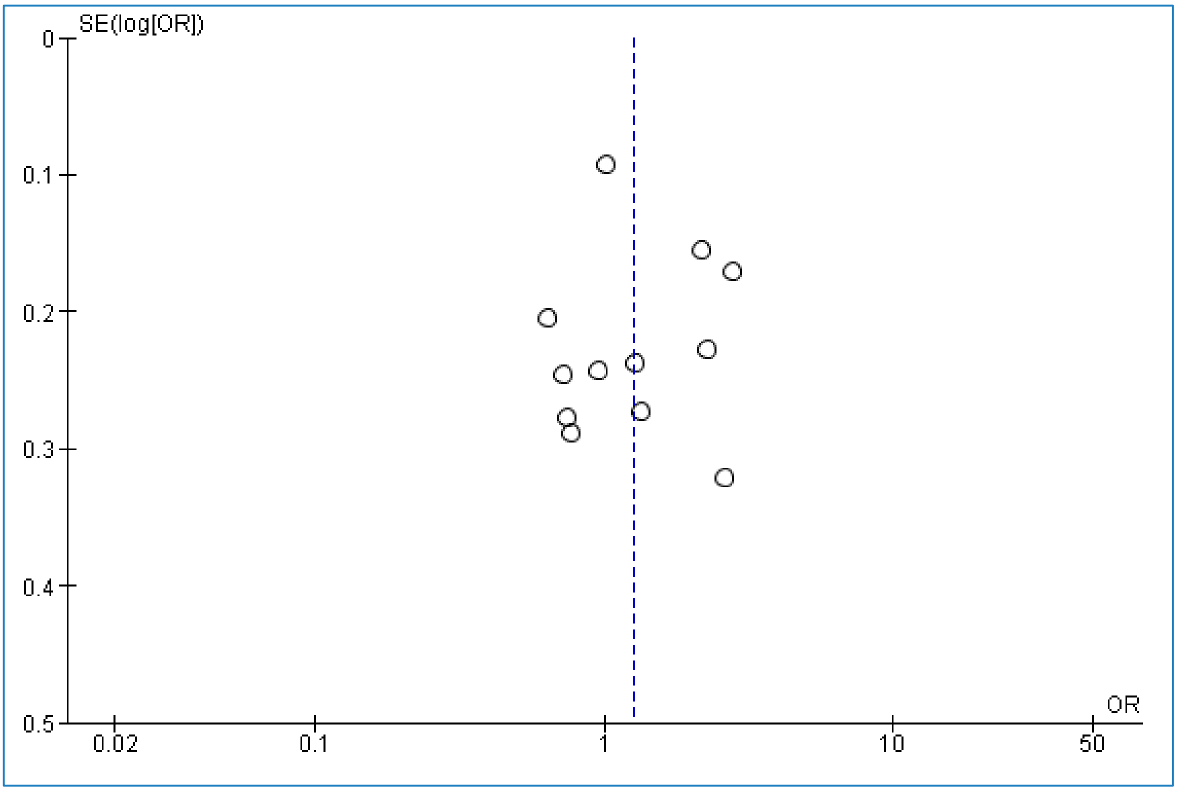


**Figure S3**: Funnel plot of association between *rs17655* polymorphism and the risk of head and neck cancer in heterozygous model

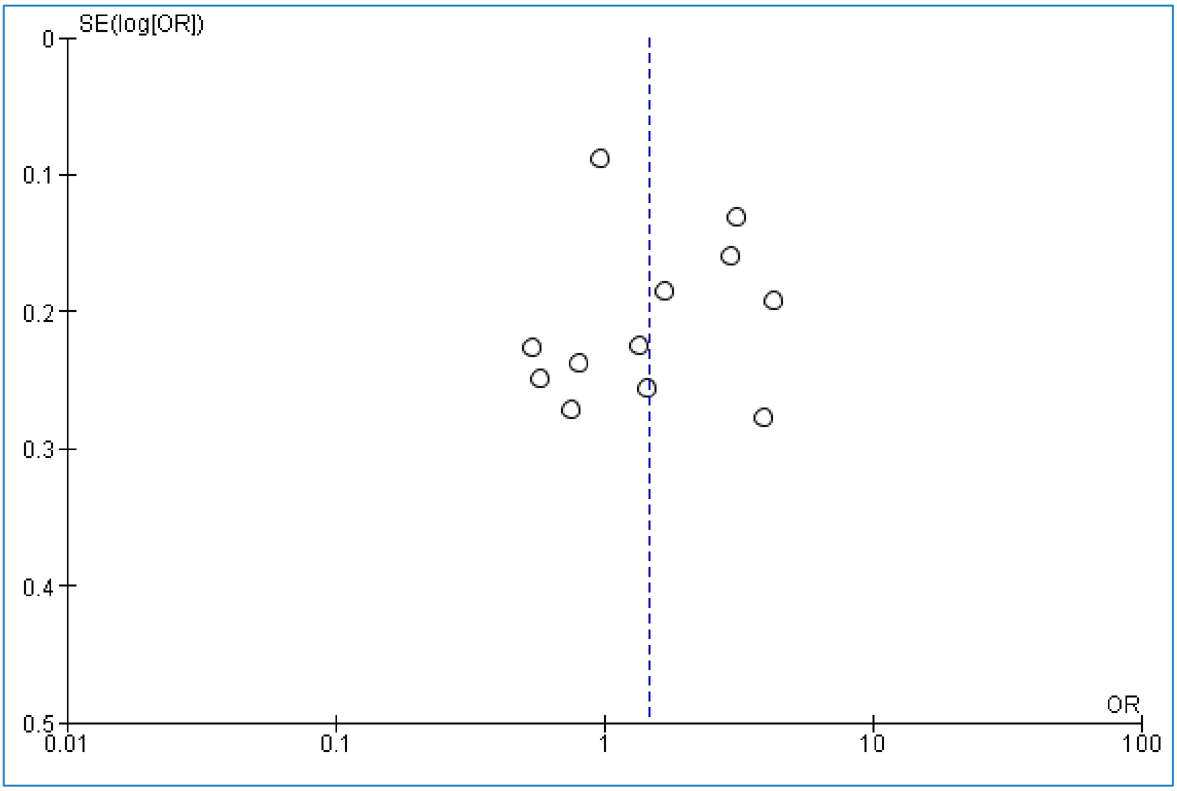


**Figure S4**: Funnel plot of association between *rs17655* polymorphism and the risk of head and neck cancer in dominant model

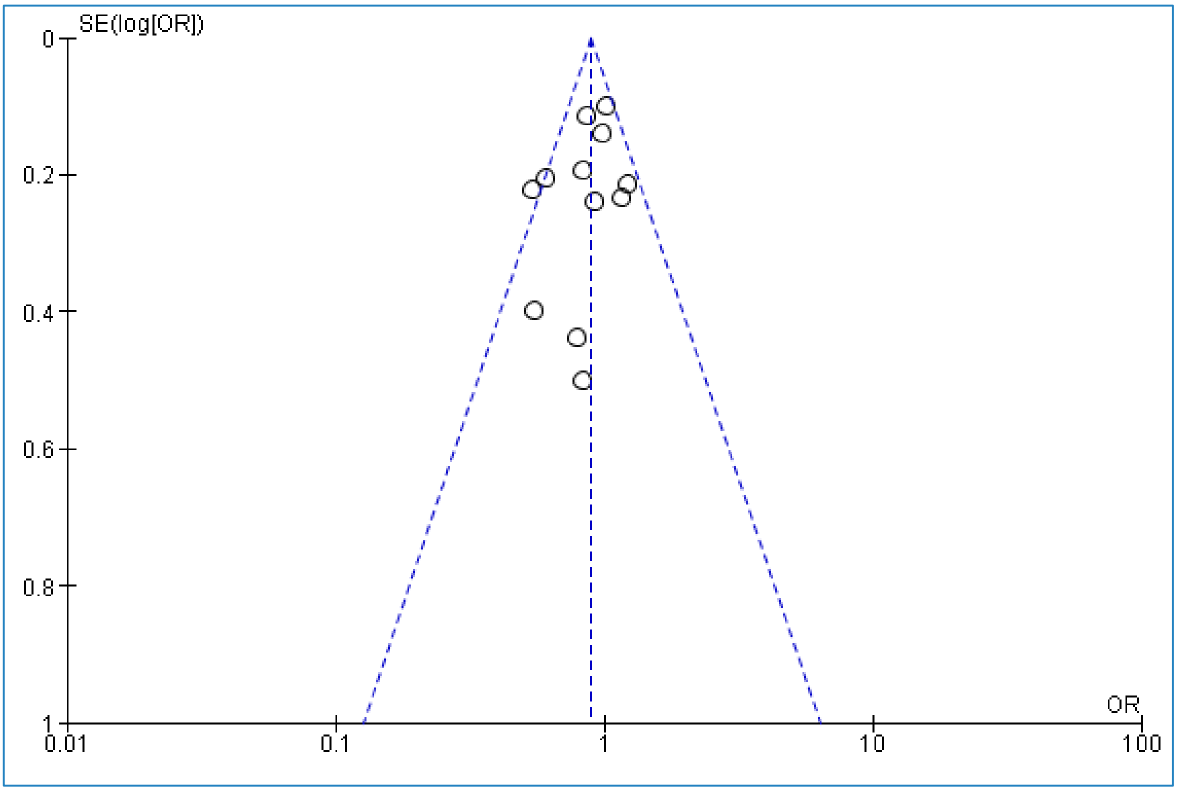


**Figure S5**: Funnel plot of association between *rs17655* polymorphism and the risk of head and neck cancer in recessive model

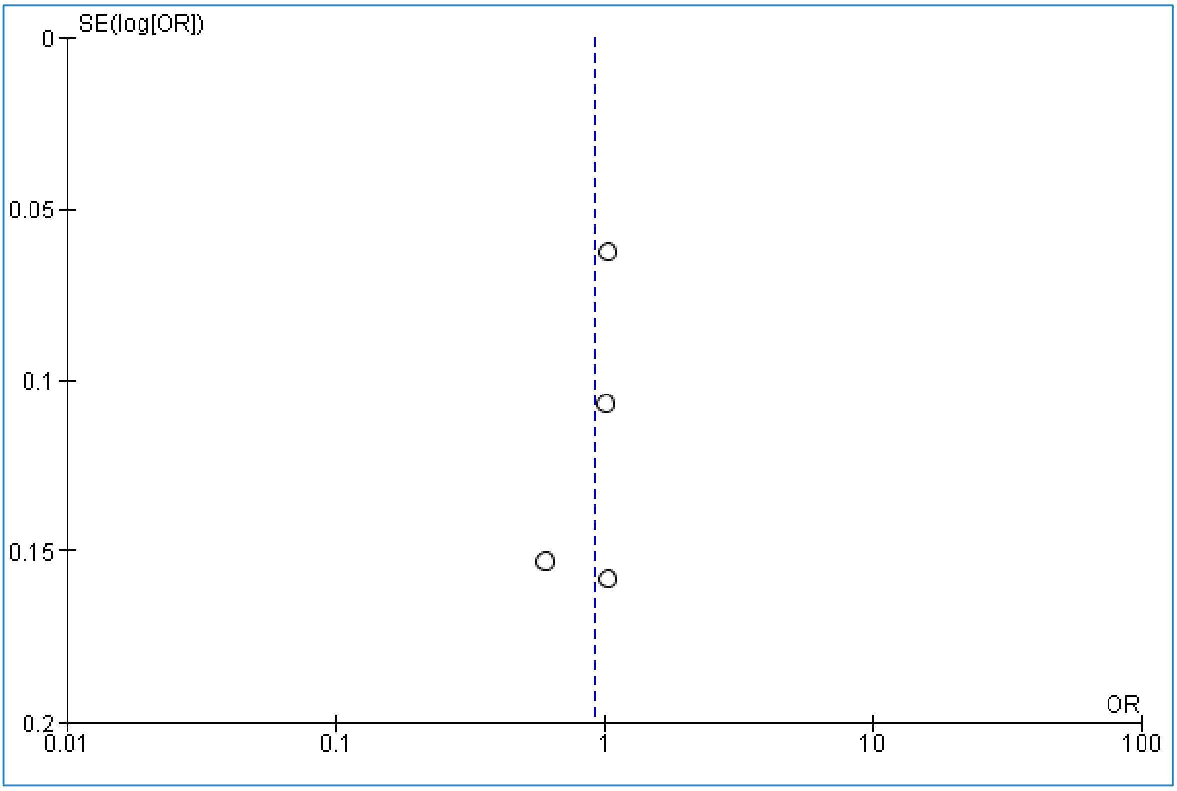


**Figure S6**: Funnel plot of association between *rs1047768* polymorphism and the risk of head and neck cancer in allelic model

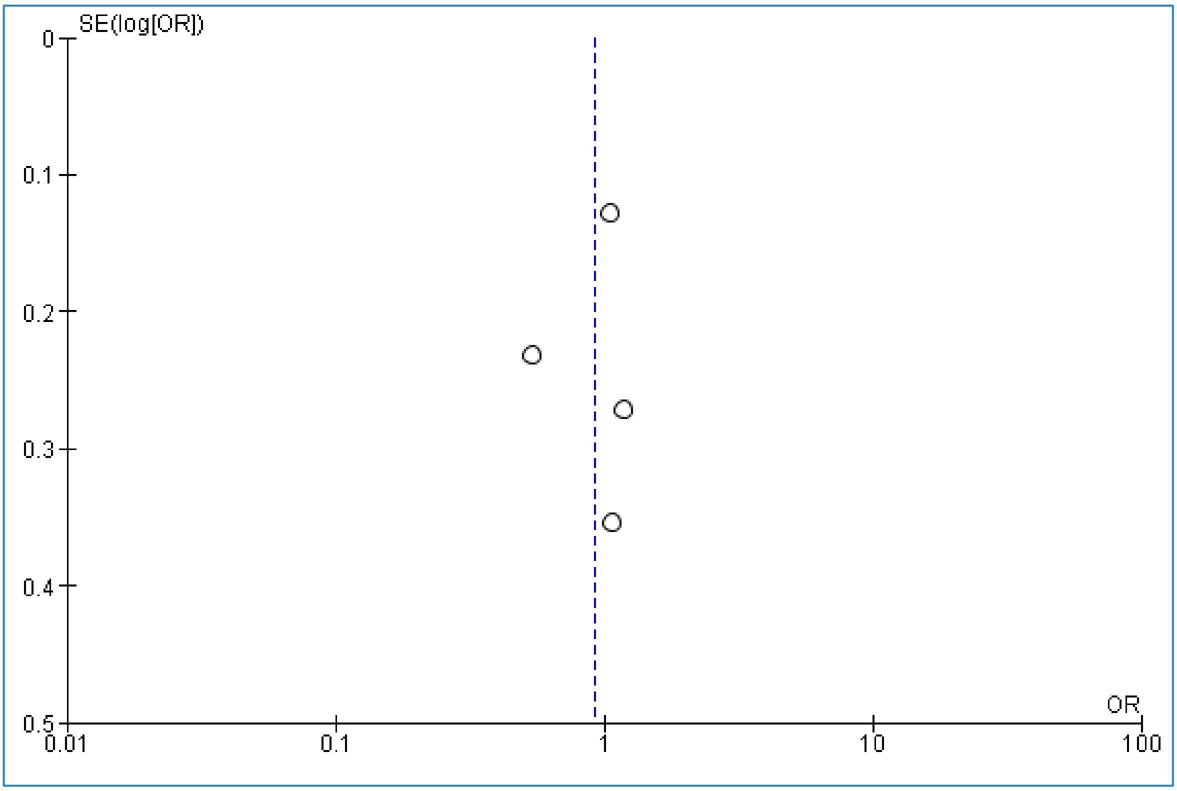


**Figure S7**: Funnel plot of association between *rs1047768* polymorphism and the risk of head and neck cancer in homozygous model

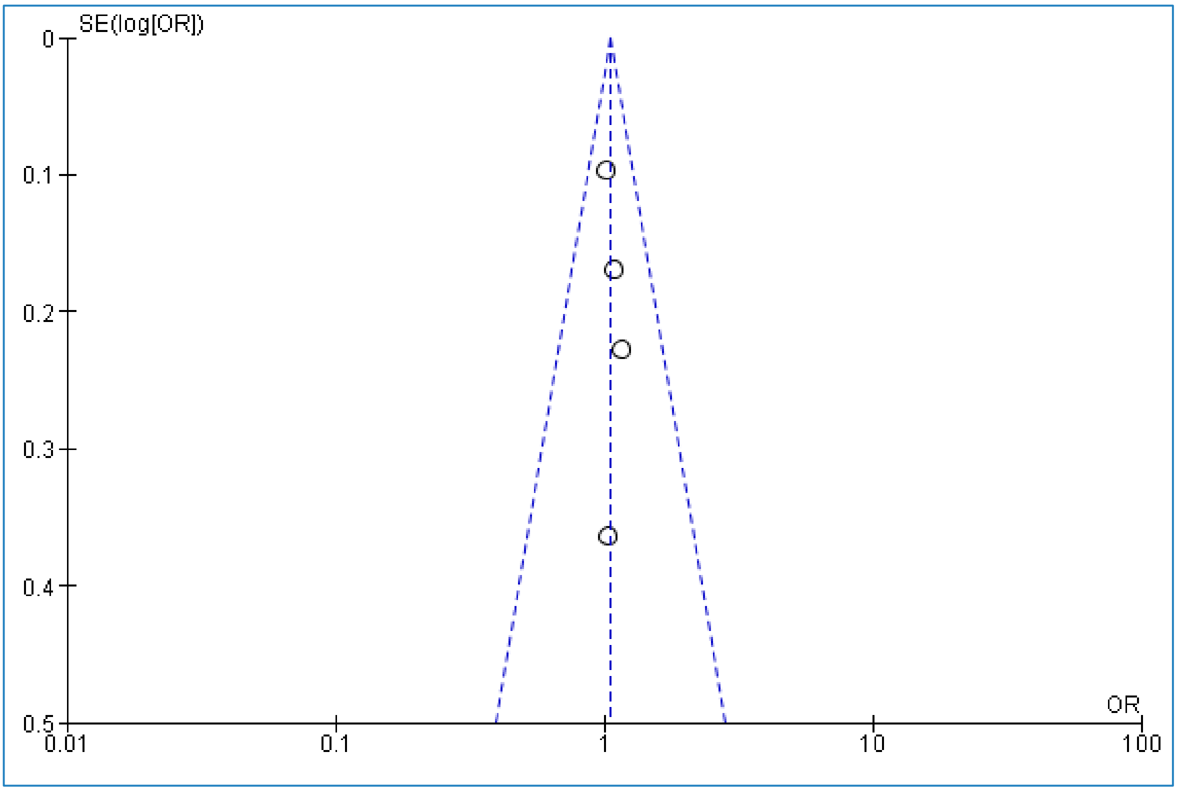


**Figure S8**: Funnel plot of association between *rs1047768* polymorphism and the risk of head and neck cancer in heterozygous model

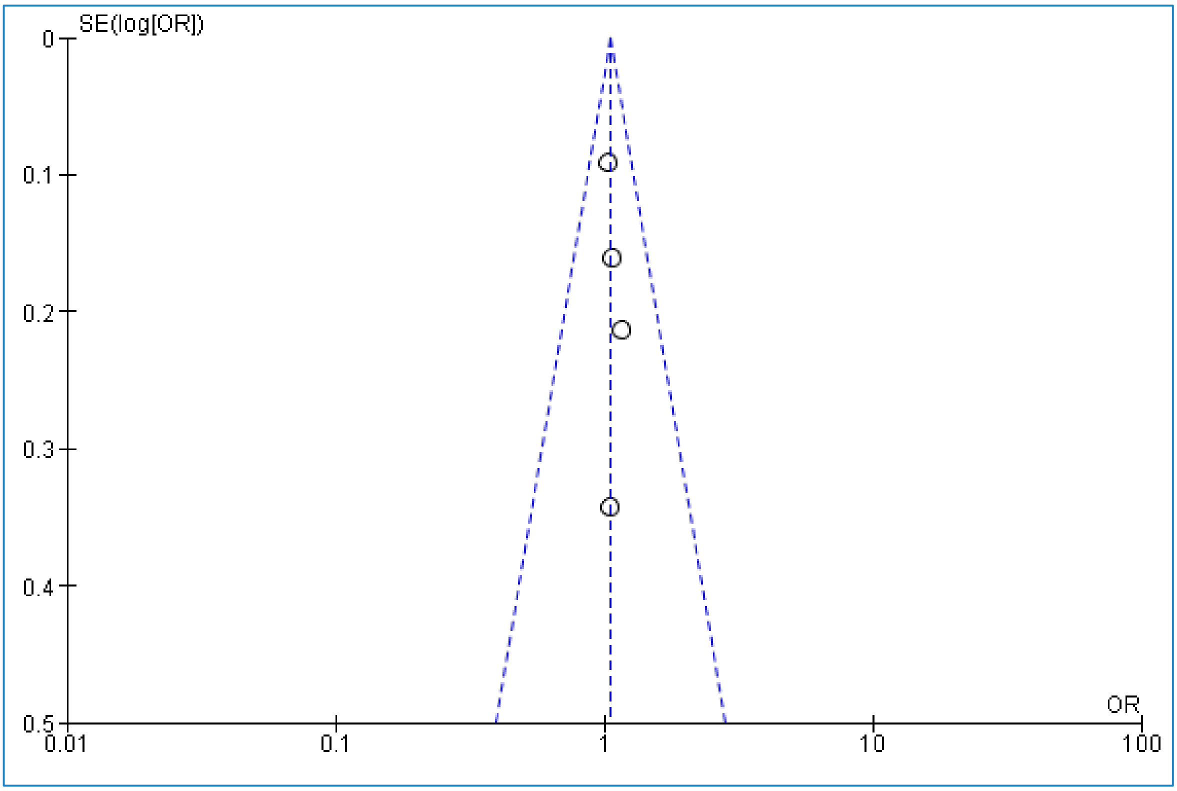


**Figure S9**: Funnel plot of association between *rs1047768* polymorphism and the risk of head and neck cancer in dominant model

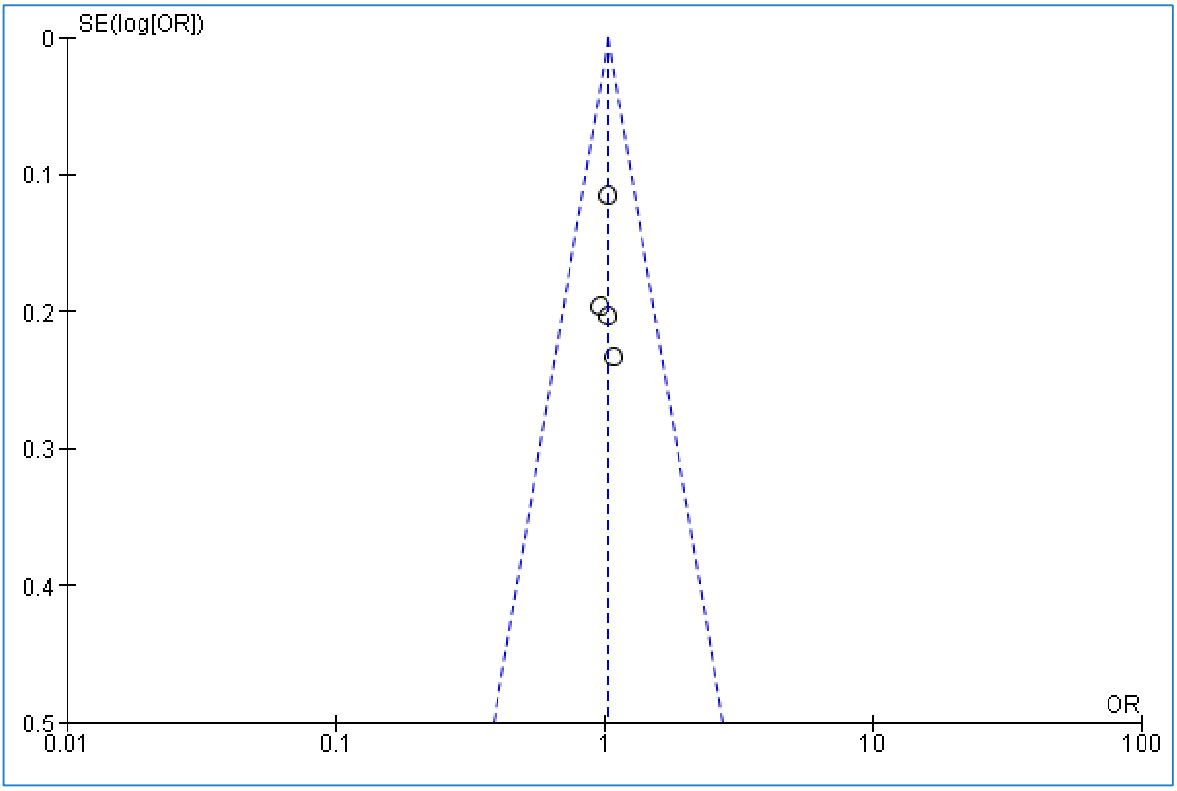


**Figure S10**: Funnel plot of association between *rs1047768* polymorphism and the risk of head and neck cancer in recessive model

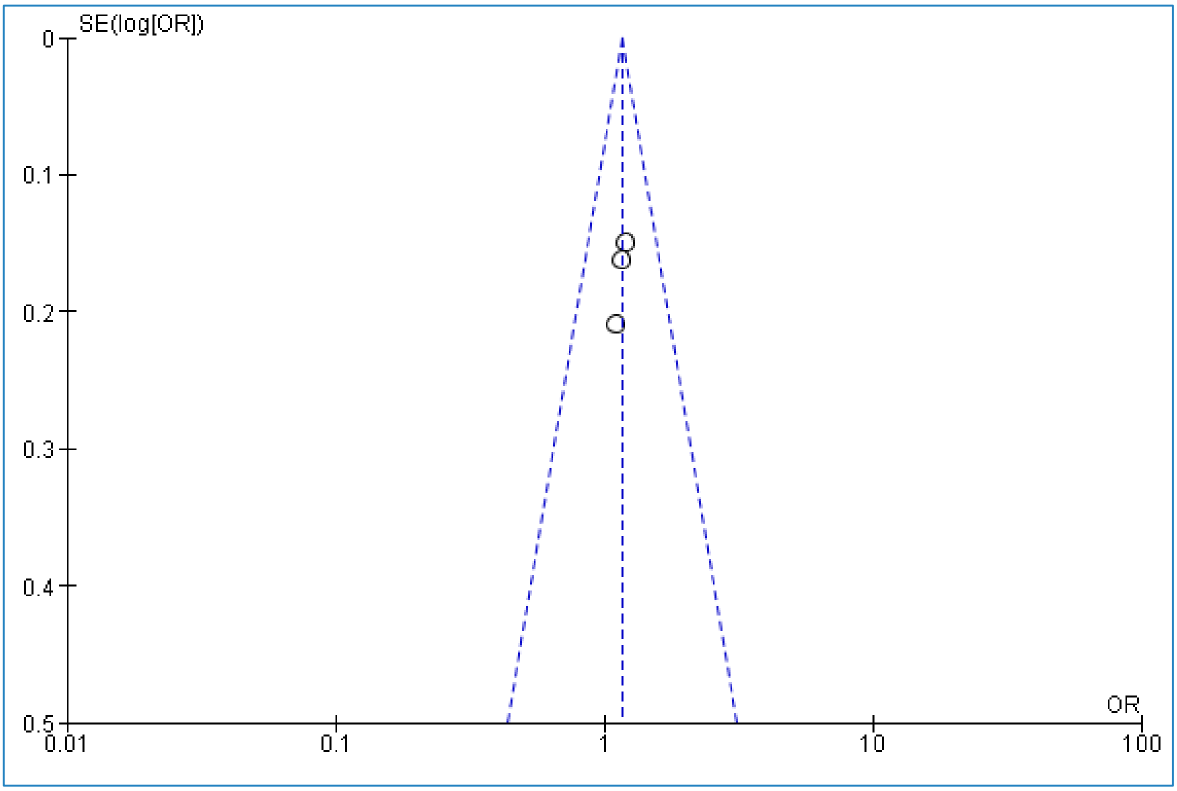


**Figure S11**: Funnel plot of association between *rs6498486* polymorphism and the risk of head and neck cancer in allelic model

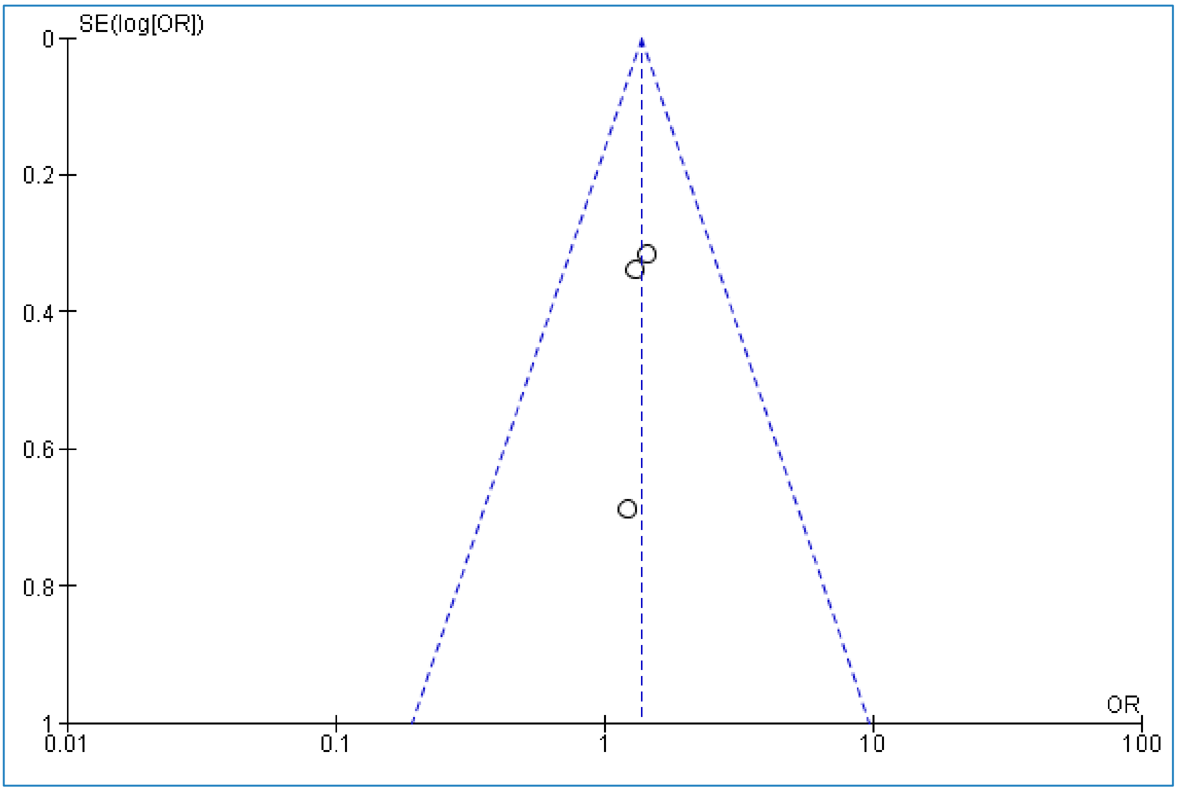


**Figure S12**: Funnel plot of association between *rs6498486* polymorphism and the risk of head and neck cancer in homozygous model

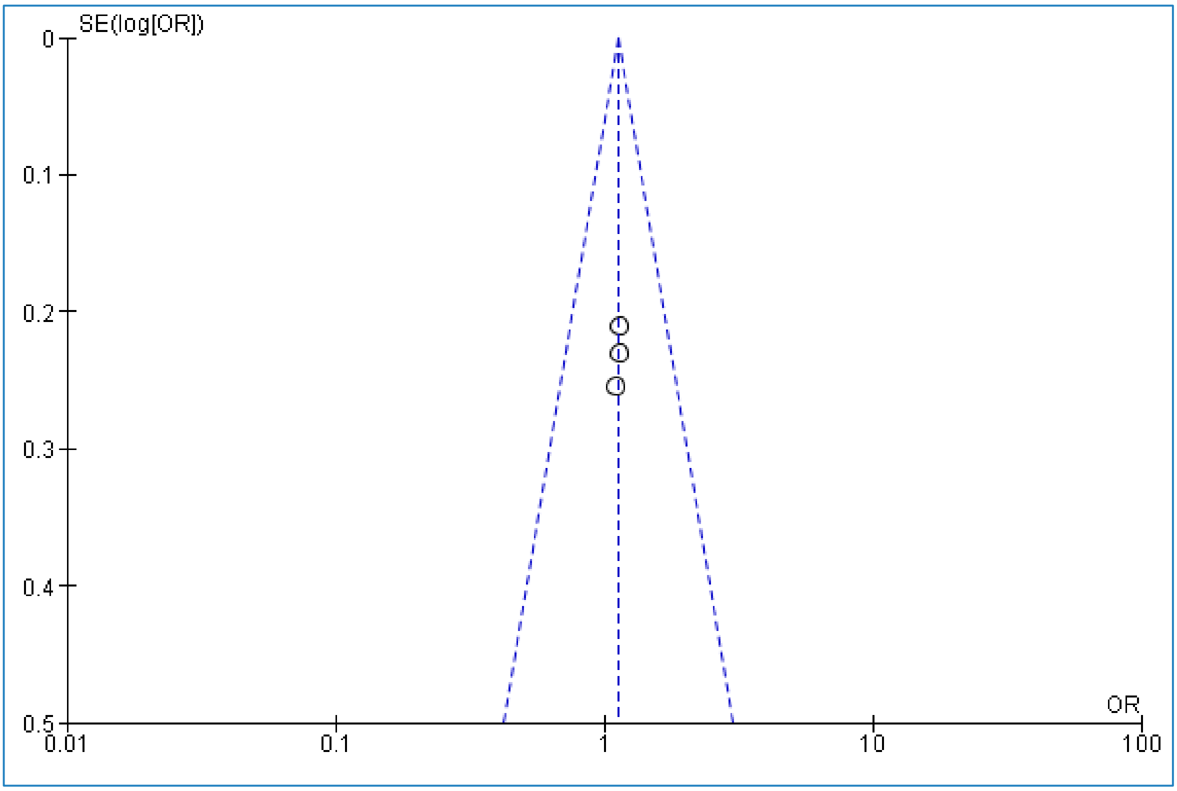


**Figure S13**: Funnel plot of association between *rs6498486* polymorphism and the risk of head and neck cancer in heterozygous model

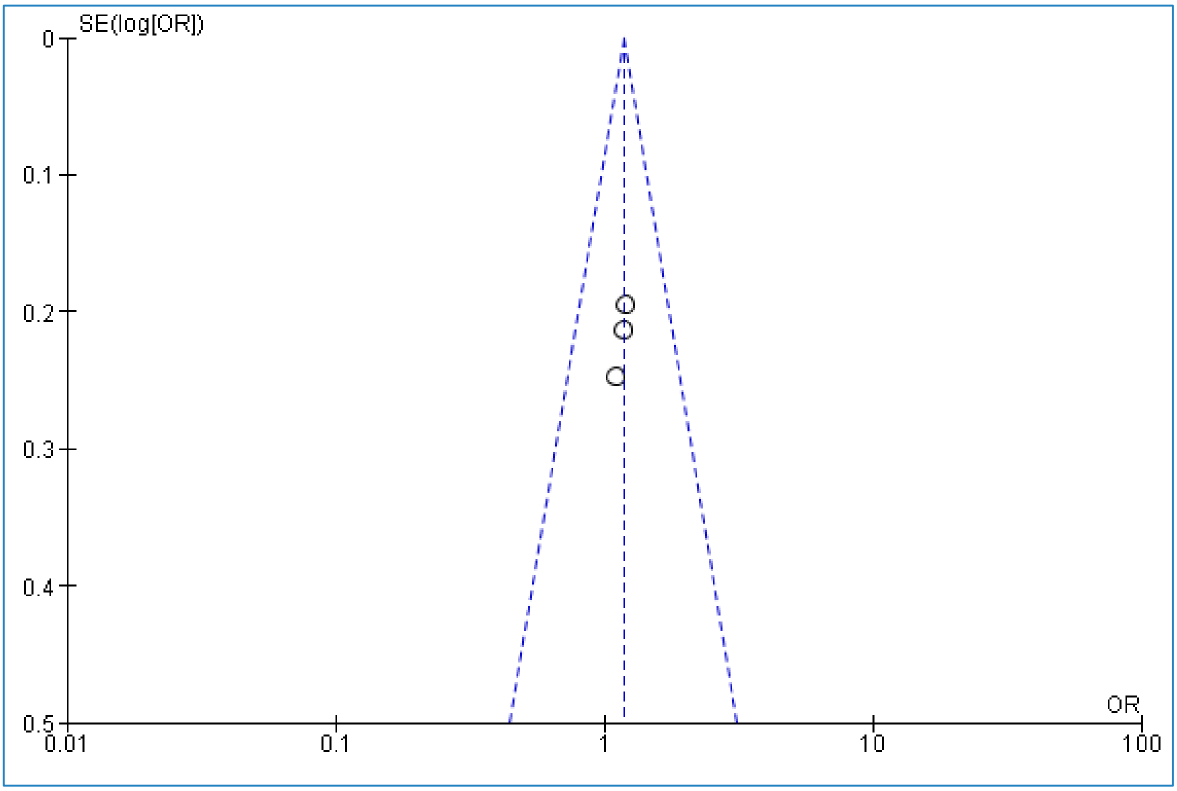


**Figure S14**: Funnel plot of association between *rs6498486* polymorphism and the risk of head and neck cancer in dominant model

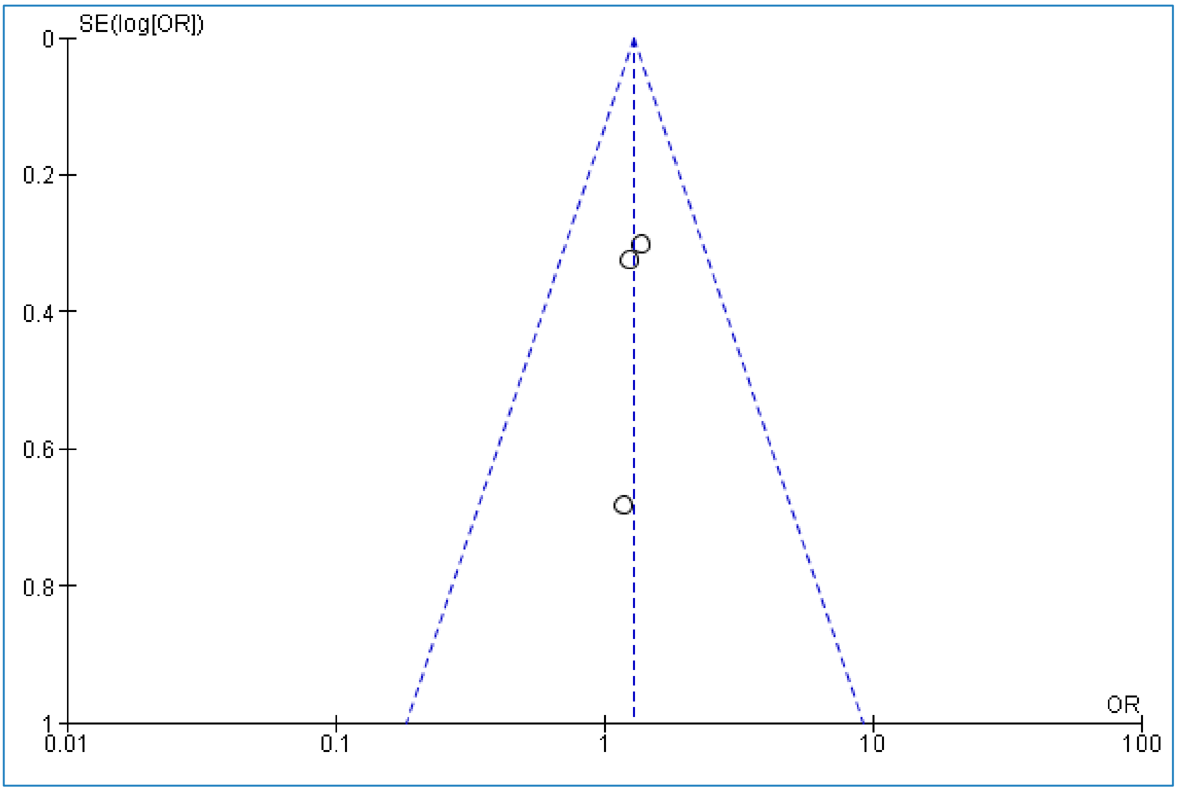


**Figure S15**: Funnel plot of association between *rs6498486* polymorphism and the risk of head and neck cancer in recessive model

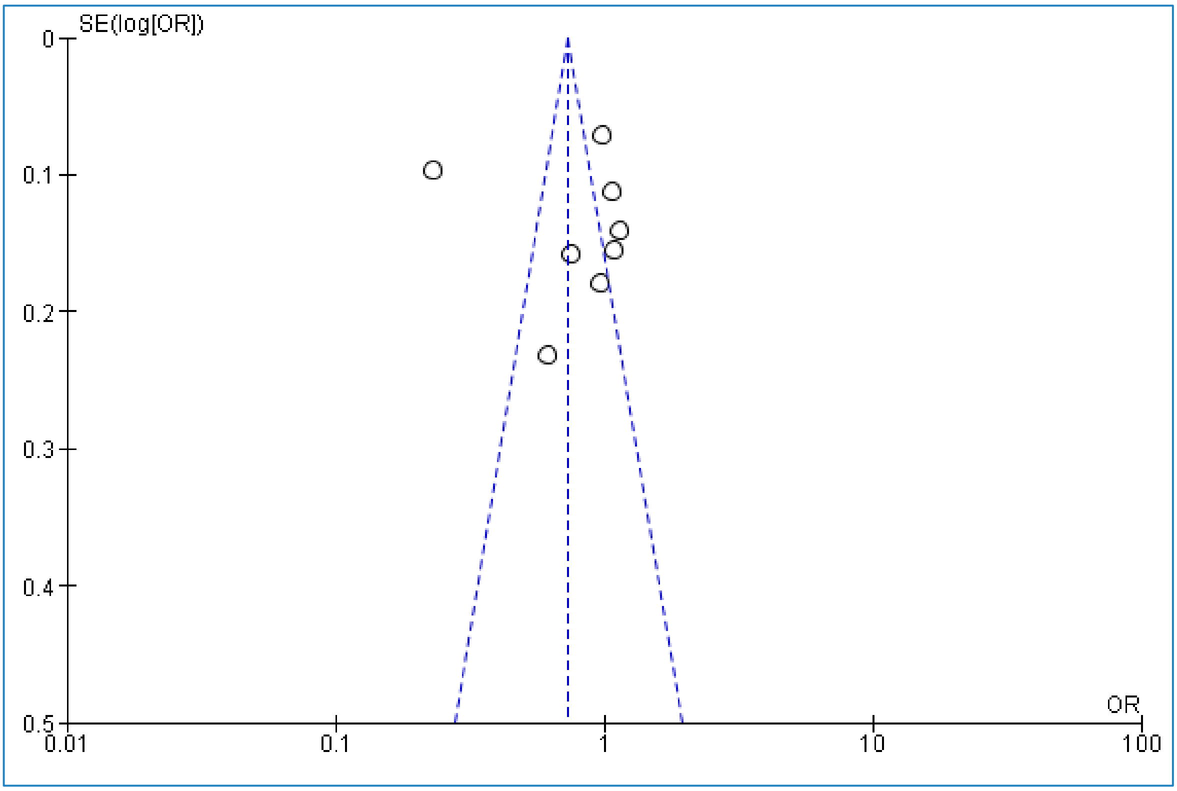


**Figure S16**: Funnel plot of association between *rs1800975* polymorphism and the risk of head and neck cancer in allelic model

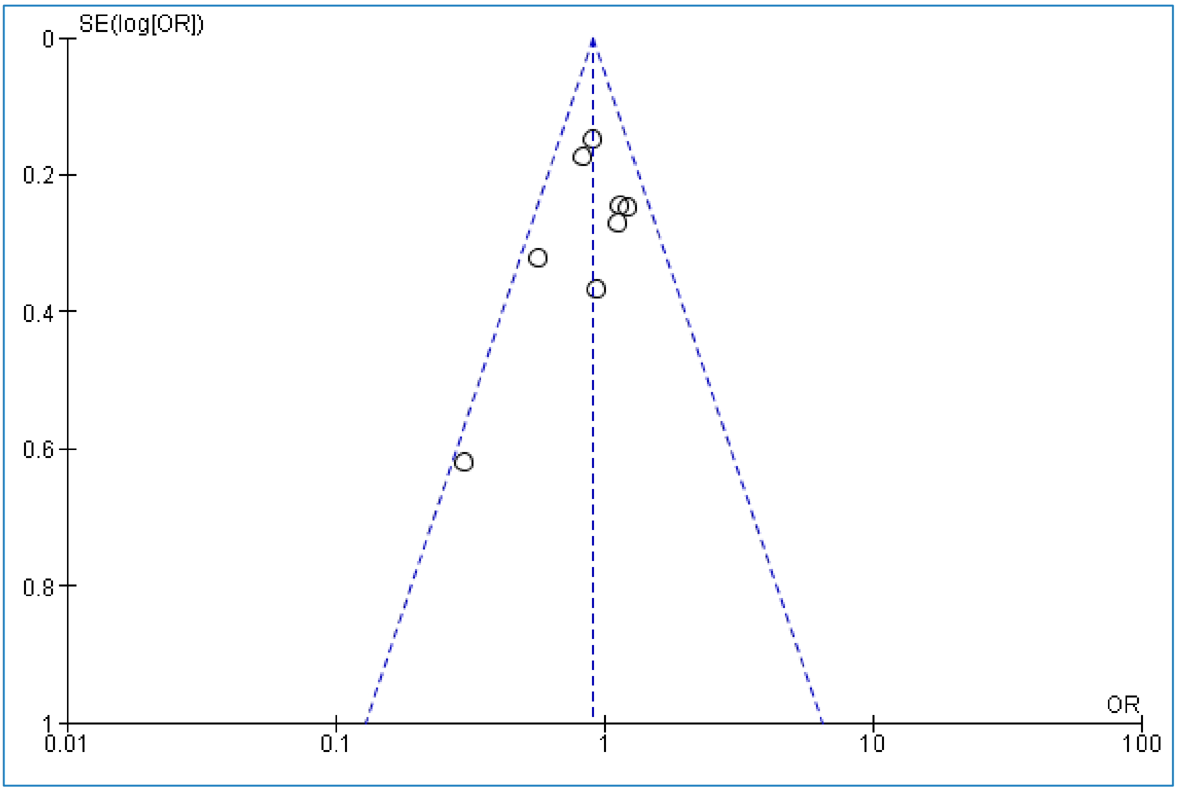


**Figure S17**: Funnel plot of association between *rs1800975* polymorphism and the risk of head and neck cancer in homozygous model

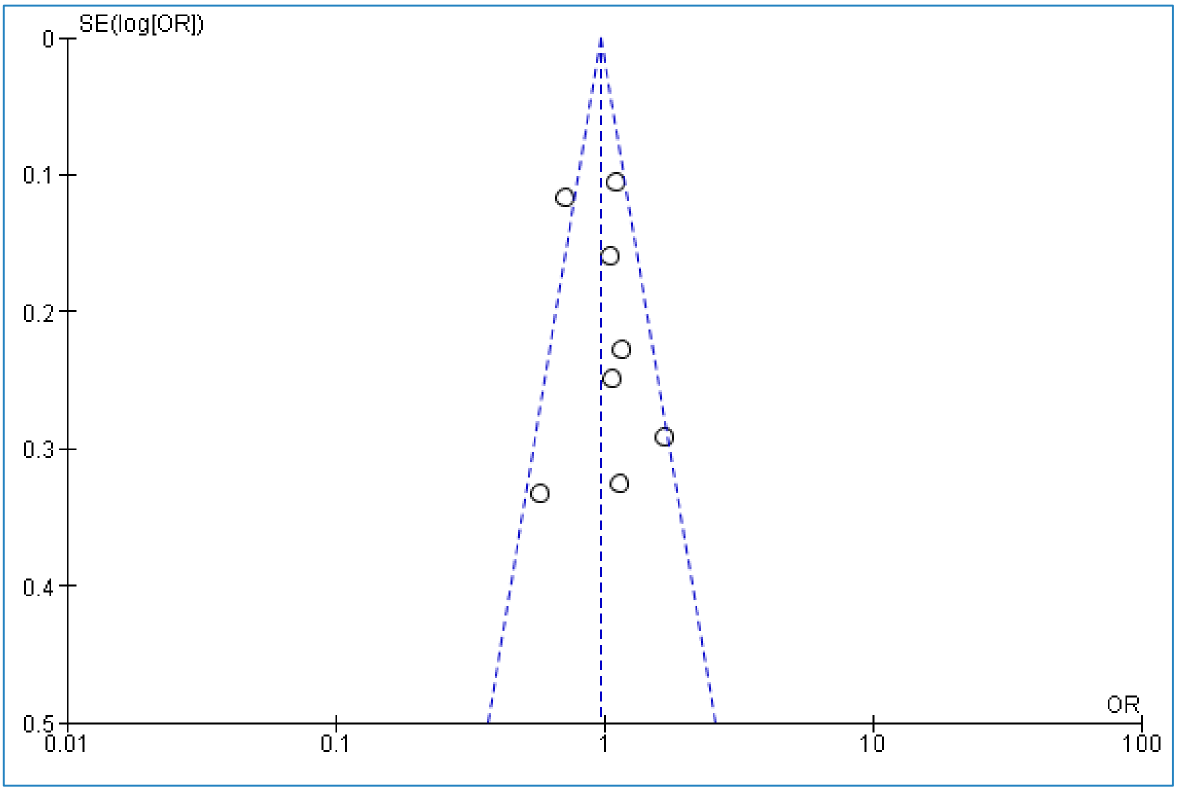


**Figure S1**8: Funnel plot of association between *rs1800975* polymorphism and the risk of head and neck cancer in heterozygous model

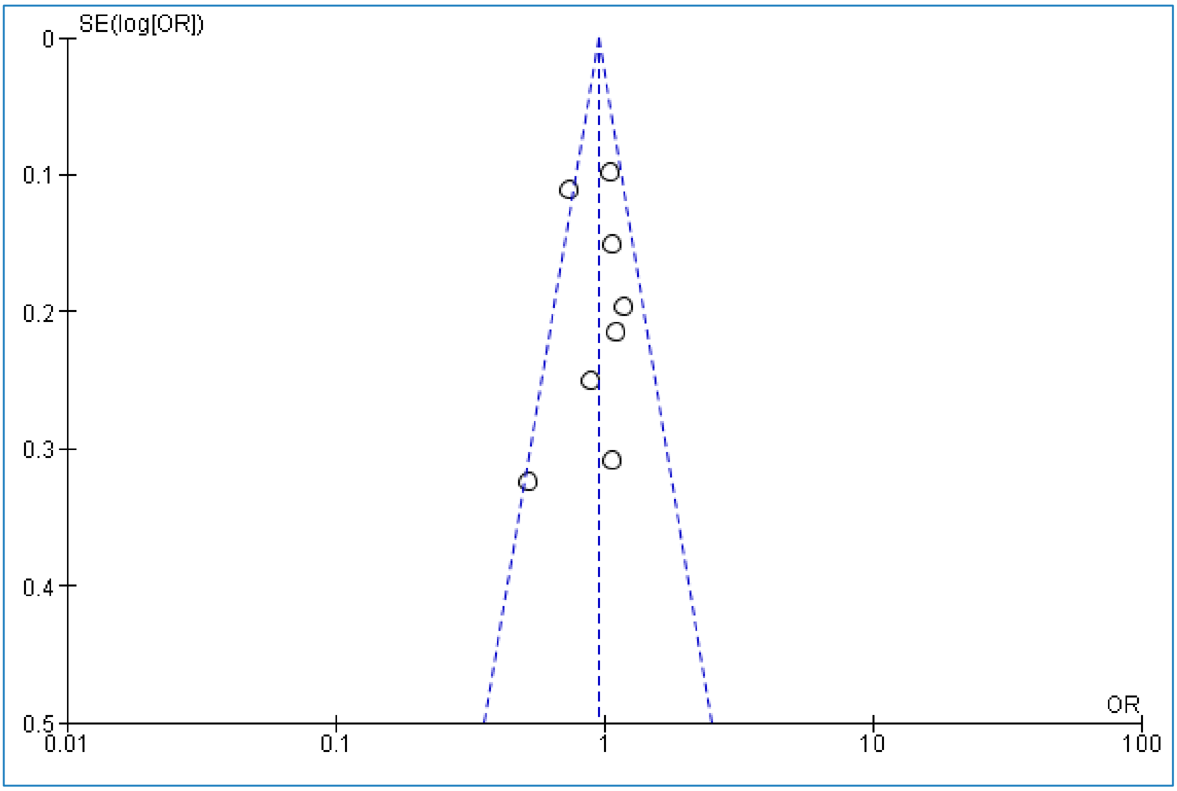


**Figure S19**: Funnel plot of association between *rs1800975* polymorphism and the risk of head and neck cancer in dominant model

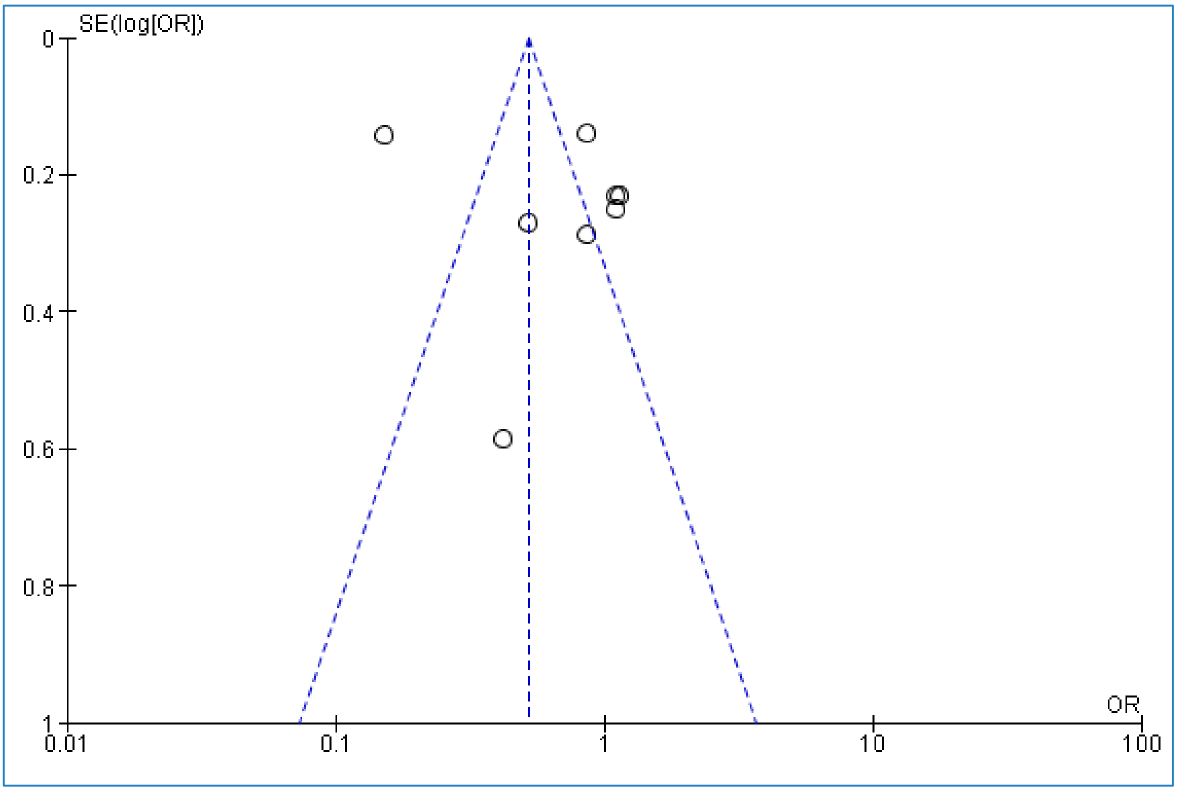


**Figure S20**: Funnel plot of association between *rs1800975* polymorphism and the risk of head and neck cancer in recessive model
